# CSF1R regulates monocyte subset differentiation and intracellular metabolism

**DOI:** 10.1038/s41467-026-75263-7

**Published:** 2026-07-04

**Authors:** Alexandre Gallerand, Johanna Merlin, Zakariya Caillot, Chloé Delaby, Elisa Bord, Jichang Han, Bastien Dolfi, Michael D. Chang, Guilhem R. Thierry, Alexia Castiglione, Sacha Grenet, Eloïse Goës, Maxime Franceschini, Gisèle Jarretou, Fairouz N. Zair, Evy Boré, Florian Tuffin, David Dombrowicz, Giulia Chinetti, Rodolphe R. Guinamard, Maxim N. Artyomov, Abdelilah Wakkach, Didier F. Pisani, Gwendalyn J. Randolph, Adeline Bertola, Patrick Auberger, Arnaud Jacquel, David A. Hume, Jesse W. Williams, Marc Bajénoff, Jaap G. Neels, Stoyan Ivanov

**Affiliations:** 1 Université Côte d’Azur, CNRS, LP2M, Nice, France; 2 Centre d’Immunologie Marseille-Luminy, Aix Marseille Univ UM 2, CNRS UMR7280, INSERM U1104, Marseille, France; 3 Université Côte d’Azur, INSERM, C3M, Nice, France; 4 Department of Pathology and Immunology, Washington University School of Medicine, St. Louis, MO USA; 5 Center for Immunology, University of Minnesota, Minneapolis, MN USA; 6 Department of Integrative Biology and Physiology, University of Minnesota, Minneapolis, MN USA; 7 Université de Lille, Inserm, CHU Lille, Institut Pasteur de Lille, U1011-EGID, Lille, France; 8 Division of Immunobiology, Department of Pathology and Immunology, Washington University, School of Medicine, St. Louis, MO USA; 9 Mater Research Institute-University of Queensland, Translational Research Institute, Brisbane, QLD Australia

**Keywords:** Monocytes and macrophages, Myelopoiesis, Enzyme mechanisms, Phosphorylation

## Abstract

Monocytes are key circulating effectors of vascular homeostasis, innate immunity and inflammation. Following their generation in mouse bone marrow, classical (Ly6C^high^) monocytes are mobilized into the blood circulation where they mature into non-classical (Ly6C^low^) patrolling monocytes or are recruited into peripheral tissues where they differentiate into tissue resident or inflammatory macrophages. Monocytes and macrophages express CSF1R (CD115), the receptor for lineage-specific growth factors CSF1 and IL-34. Here, we report that acute CSF1R blockade or genetic deletion negatively interferes with monocyte intracellular metabolism and reduces blood Ly6C^low^ monocytes in part by blunting differentiation of Ly6C^high^ monocytes. Based upon lineage-specific deletion of Glutamine-Fructose-6-Phosphate Transaminase 1 (GFPT1), the hexosamine biosynthetic pathway (HBP) is identified as an important regulator of CSF1R expression and monocyte subsets. Inhibition of receptor tyrosine kinases CSF1R and FLT3 rewires and partially impairs metabolic activity in human monocytes. Our findings provide insights into the link between CSF1R signaling, metabolic regulation, and monocyte survival and differentiation.

## Abstract (web summary)

CSF1R (CD115) is an important growth factor receptor for monocytes and macrophages. Here the authors show in mouse models of genomic deletion and by chemical inhibition in human monocytes that metabolic regulation is an integrant part of CSF1R downstream signalling and is itself regulated metabolically via the hexosamine biosynthetic pathway.

## Introduction

Monocytes are innate immune cells generated in bone marrow by a tightly and highly regulated process named myelopoiesis^1^. Multiple consecutive steps lead to monocyte generation from hematopoietic stem cells (HSC) through several intermediate committed myeloid progenitors^1^. Among those, granulocyte/macrophage progenitor (GMP) give rise to the majority of blood circulating monocytes^2^. In mouse blood, two major monocyte subsets have been identified. Ly6C^high^ (classical monocytes) that express the chemokine receptor CCR2, infiltrate into peripheral tissues and contribute to tissue macrophage pool size at steady-state and upon inflammation^3–6^. Less is known about the generation and functions of Ly6C^low^ (non-classical) monocytes, previously shown to patrol blood endothelial cells and provide growth factors^7,8^. These populations are conserved in humans and correspond to CD14^+^CD16^-^ and CD14^-^CD16^+^ monocytes, respectively^9^. While most blood monocytes originate from GMPs, recent evidence indicates that monocyte/dendritic progenitors (MDP) also generate a defined subset of blood monocytes^2,10,11^.

Hematopoietic stem cell commitment to the monocyte-macrophage lineage is associated with inducible expression of *Csf1r*, encoding the receptor for macrophage colony-stimulating factor (CSF1) and a second ligand, IL-34^12^. CSF1R protein is a lineage-restricted marker, expressed by all committed myeloid progenitors, blood monocytes and tissue-resident macrophages^9,13,14^. CSF1R (CD115) is a class III receptor tyrosine kinase (RTK) structurally related to FLT3 (CD135) and KIT (CD117), which are also expressed by myeloid progenitors. CSF1 promotes the proliferation and differentiation of mouse bone marrow cells or human monocytes in vitro to produce bone marrow-derived macrophages, or monocyte-derived macrophages, two widely-used models for the study of macrophage biology^15^. In vivo, administration of recombinant CSF1, or a CSF1-Fc fusion protein to mice, causes a profound and selective monocytosis and proliferative expansion of tissue-resident macrophage populations^16–18^. Reciprocally, mutations in the *Csf1* and *Csf1r* genes in mice and rats lead to severe osteopetrosis (due to osteoclast deficiency) and the loss of many tissue-resident macrophage populations^19,20^. Notwithstanding these observations, multiple lines of evidence indicate that CSF1R signaling is not absolutely required for monocytopoiesis. Instead, treatment of mice with blocking anti-CSF1R or anti-CSF1 antibodies leads to selective loss of Ly6C^low^ non-classical monocytes^21–23^. Similarly, inducible genetic deletion of *Csf1r* or endothelial *Csf1* affected Ly6C^low^ but not Ly6C^high^ monocytes^24,25^. A *Csf1r* enhancer mutation that abolishes CSF1R expression and CSF1 responsiveness in bone marrow progenitors had no effect on blood monocyte numbers and subset diversity^26^.

In macrophages, intracellular metabolism and dedicated functions are closely linked. CSF1 binding to CSF1R triggers Glut1-dependent glucose uptake and favors glucose-dependent intracellular metabolism^27^. Our recent work demonstrated that glucose metabolism sustains monocyte CSF1R expression, and inhibiting glucose uptake was associated with decreased bone marrow, blood, and spleen monocyte numbers^28^. How CSF1R signaling and glucose intracellular metabolism interconnect to regulate monocyte homeostasis remains unclear.

In the present study, we explore whether and how the inter-connection of CSF1R signaling and intracellular metabolism regulates monocyte survival and maturation. We report that Ly6C^high^ monocytes require permanent glucose-fueled de novo protein synthesis to sustain CSF1R expression and engage their differentiation into the Ly6C^low^ subset. Mechanistically, invalidation of the hexosamine biosynthetic pathway (HBP) enzyme Glutamine-Fructose-6-Phosphate Transaminase 1 (GFPT1) in myeloid cells reduces monocyte CSF1R expression, glucose uptake, and protein synthesis, and dysregulates monocyte subset proportions. Similar to murine Ly6C^high^ monocytes, human classical blood monocytes display a higher avidity for glucose compared to the non-classical subset, and inhibition of receptor tyrosine kinases CSF1R and FLT3 impairs their metabolic activity and the survival of CD14^+^ CD16^+^ cells. Our observations expand the understanding of the role of CSF1R signaling in murine and human monocytes and could inform on ways to manipulate these cells.

## Results

### CSF1R expression in monocytes requires constant protein synthesis and membrane export

**Fig. 1:**
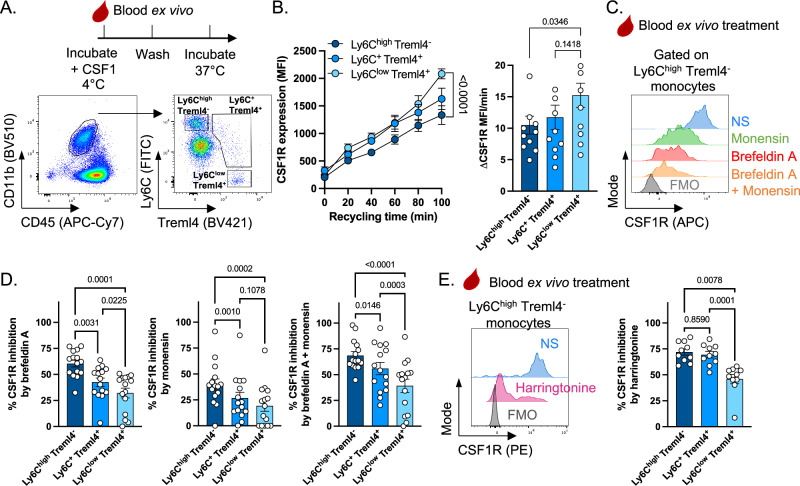
Differential maintenance of surface CSF1R expression by protein translation across monocyte subsets. **A** Experimental scheme used to measure CSF1R renewal after CSF1 binding (top) and representative gating strategy used to identify blood monocyte subsets (bottom). **B** Time-course analysis (left) and quantification (right) of surface CSF1R expression on blood monocyte subsets from wild-type mice (*n* = 4 mice recorded from 0 to 80 min, *n* = 7 mice recorded from 0 to 100 min) following incubation at 37 °C to allow receptor recycling. Quantification as MFI per minute (right plot) is shown for each individual mouse. Data pooled from three independent experiments. Representative histograms (**C**) and quantification (**D**) of CSF1R inhibition on blood monocyte subsets from wild-type mice (*n* = 15) after a 45-min incubation at 37 °C with brefeldin A, monensin or both inhibitors. Data pooled from four independent experiments. **E** Quantification of CSF1R inhibition on each monocyte subset from wild-type mice (*n* = 10) following a 45-min incubation at 37 °C with harringtonine. Data pooled from two independent experiments. Repeated-measures one-way ANOVA corrected for multiple comparisons using Dunnett’s multiple comparison tests were used for statistical analysis in panels (**B**) (right), **D** and **E**. A mixed-effects model corrected for multiple comparisons using Dunnett’s multiple comparisons test was used for statistical analysis in panel (**B**) (left). Data are presented as mean values ± SEM. See also Supplementary Fig. 1. Source data are provided as a Source Data file.

Previous studies documented that the administration of anti-CSF1R antibodies to mice robustly reduced blood monocyte counts, a phenotype sometimes observed on both Ly6C^high^ and Ly6C^low^ monocytes^29,30^ or solely on Ly6C^low^ monocytes^22–24,31^. We also observed that downregulation of CSF1R precedes loss of blood monocytes^28^. CSF1R is rapidly internalized following ligand binding, ubiquitinated, and degraded^19,32^. The response to CSF1 analyzed in bone marrow-derived macrophages suggests that macrophages require continuous synthesis of the receptor and its reappearance on the cell surface. However, whether this mechanism is conserved in monocyte subsets is not yet documented. The rate of receptor synthesis can be monitored by flow cytometry in conditions in which CSF1-repleted cells are washed free of ligand^33–35^. To determine CSF1R re-appearance rate in blood monocyte subsets, we incubated blood with recombinant CSF1 at 4 °C to mask surface epitopes and then washed the cells thoroughly to remove any potentially unbound CSF1 protein. Cells were then kept on ice (baseline) or incubated at 37 °C (without CSF1) for up to 100 min to allow the turnover of the receptor (Fig. 1A). Monocyte subsets were defined according to their expression of Ly6C and Treml4, allowing to identify three differentiation stages^36^. Surface CSF1R expression gradually reappeared over time in Ly6C^high^ Treml4^-^ (Ly6C^high^), Ly6C^+^ Treml4^+^ (Ly6C^int^) and Ly6C^low^ Treml4^+^ (Ly6C^low^) monocytes (Fig. 1B and Supplementary Fig. 1A). Importantly, CSF1R export rate was higher in Ly6C^low^ compared to Ly6C^high^ monocytes (Fig. 1B). This observation agrees with the higher dependence of these cells on CSF1R signaling for their survival.

To explore mechanisms regulating surface CSF1R expression on monocyte subsets, we inhibited Golgi-dependent trafficking with monensin and endoplasmic reticulum (ER) to Golgi transfer with brefeldin A. Monensin induced a rapid decrease in membrane CSF1R expression on Ly6C^high^, Ly6C^int^ and Ly6C^low^ monocytes (Fig. 1C and Supplementary Fig. 1B). Brefeldin A depleted CSF1R to a greater extent in all blood monocytes (Fig. 1C and Supplementary Fig. 1B). Of note, a combined treatment with monensin and brefeldin A did not completely abrogate surface CSF1R expression on blood monocyte subsets (Fig. 1C and Supplementary Fig. 1B). The depletion of CSF1R in response to monensin- and brefeldin was greater in Ly6C^high^ monocytes in comparison to Ly6C^int^ and Ly6C^low^ monocytes (Fig. 1D). Inhibition of protein translation with harringtonine also rapidly reduced CSF1R surface expression on all blood monocyte subsets (Fig. 1E). Harringtonine led to a greater inhibition of surface CSF1R expression in Ly6C^high^ and Ly6C^int^ monocytes in comparison to the Ly6C^low^ subset (Fig. 1E). Together, these results demonstrated that CSF1R expression in blood monocytes is foremost dependent on continuous protein translation and addressing to the cell membrane following maturation in the Golgi apparatus, rather than receptor endocytosis and recycling. Of note, Ly6C^low^ non-classical blood monocytes demonstrated higher receptor re-appearance rate and lower translation dependence. The mechanisms regulating membrane CSF1R expression thus appeared different across blood monocyte subsets, reflecting their singular reliance on this receptor.

### CSF1R regulates blood Treml4^+^ monocytes

**Fig. 2:**
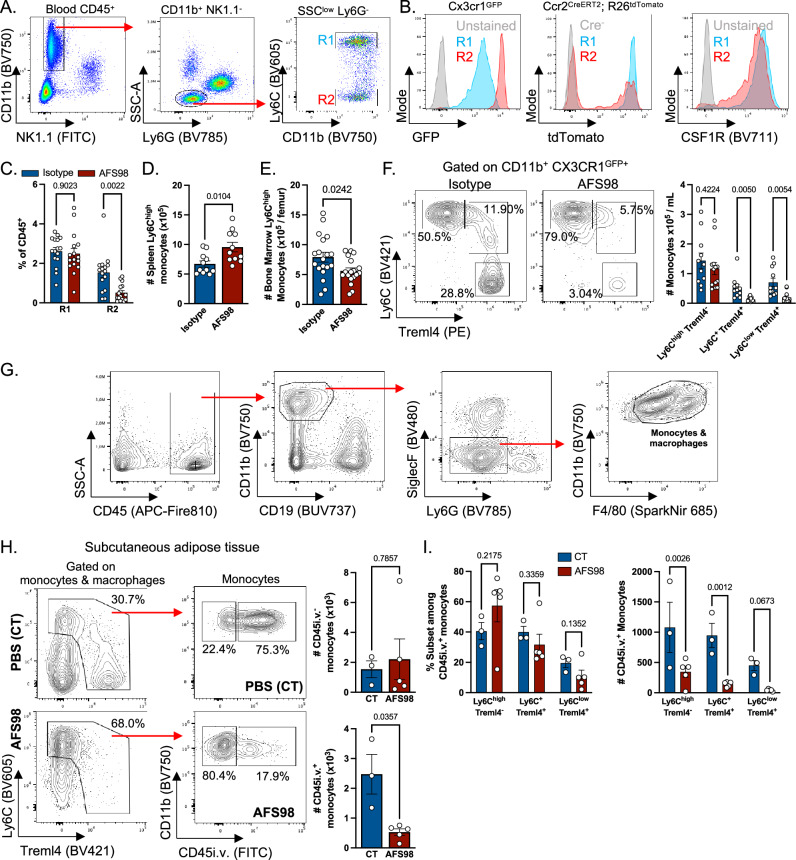
CSF1R blockade depletes Treml4^+^ monocytes. **A** Gating strategy used to identify Ly6C^high^ (R1) and Ly6C^low^ (R2) cells without including CSF1R. **B** Histograms representing expression of GFP in Cx3cr1^GFP^ mice, tdTomato in CCR2^CreERT2^; R26^tdTomato^ mice, and CSF1R by R1 and R2 cells. tdTomato expression was assessed in CCR2^CreERT2^; R26^tdTomato^ mice 2 days after tamoxifen administration. **C** Proportions of R1 and R2 cells among total immune cells following isotype (*n* = 16) or AFS98 (*n* = 16) treatment. Data pooled from four independent experiments. **D** Quantification of spleen Ly6C^high^ monocytes identified as CD45^+^ CD11b^+^ Ly6C^high^ CX3CR1^GFP+^ cells in CX3CR1^GFP/+^ mice that received isotype (*n* = 11) or AFS98 (n = 11) treatment. Data pooled from two independent experiments. **E** Quantification of bone marrow Ly6C^high^ monocytes identified as CD11b^+^ Ly6G^-^ Ly6C^high^ cells in mice that received isotype (*n* = 19) or AFS98 (*n* = 18) treatment. Data pooled from four independent experiments. (**F**) Quantification of blood monocyte subsets in mice that received isotype (*n* = 11) or AFS98 (*n* = 13) treatment. **G** Gating strategy used to identify monocyte subsets in subcutaneous adipose tissue. Representative flow cytometry plots and quantifications of total monocytes (**H**) and their subsets (**I**) in subcutaneous adipose tissue from mice treated with PBS (CT, *n* = 3) or AFS98 (*n* = 5) and injected intravenously with 1 μg anti-CD45-FITC 5 min before culling. A two-way repeated measures ANOVA was used in panel (**C**), without correction for multiple comparisons (uncorrected Fisher’s LSD). Two-way repeated measures ANOVA test, corrected for multiple comparisons using Tukey’s multiple comparison tests, were used in panels (**F** and **I**). Two-sided unpaired Mann–Whitney tests were used for statistical analysis in panels (**D**, **E** and **H**). Data are presented as mean values ± SEM. See also Supplementary Fig. 2. Source data are provided as a Source Data file.

We next sought to explore the role of CSF1R on all blood monocyte subsets. Since this precludes the use of CD115 as a monocyte identification marker, we devised an alternative gating strategy. Monocytes were defined as CD45^+^ CD11b^+^ NK1.1^-^ Ly6G^-^ SSC^low^ cells then further divided into Ly6C^high^ (R1) and Ly6C^low^ (R2) subsets (Fig. 2A). Using Cx3cr1^GFP^ mice^37^ we observed a robust expression of CX3CR1 on both R1 and R2 cells, with the latter showing higher and homogeneous expression of the reporter in comparison to R1 cells (Fig. 2B). In contrast, using CCR2^CreERT2^; R26^tdTomato^ mice^38^, we detected higher tdTomato expression in the R1 population compared to R2 (Fig. 2B). Both R1 and R2 cells were CD115-positive in untreated control mice (Fig. 2B).

Using this gating strategy, we analyzed the impact of CSF1R blockade on monocyte subsets. Wild-type C57BL/6 J mice were injected intraperitoneally with CSF1R blocking Ab (AFS98) or isotype control twice 48 h apart and were analyzed 24 h after the second injection to quantify R1 and R2 cells in blood. As reported by others^22^, AFS98 administration had no effect on blood Ly6C^high^ monocytes but selectively reduced Ly6C^low^ monocytes (Fig. 2C). These data demonstrated that Ly6C^low^ monocytes, but not Ly6C^high^ monocytes, are sensitive to CSF1R blockade. We performed a similar experiment in Cx3cr1^GFP/+^ mice and also observed that Ly6C^low^ monocyte numbers were diminished by 50% upon AFS98 administration (Supplementary Fig. 2A). We used Uniform Manifold Approximation and Projection (UMAP) dimensional reduction of spectral flow cytometry data to obtain a clearer picture of monocyte phenotype following AFS98 Ab administration (Supplementary Fig. 2B). General UMAP distribution of CD11b^+^ CX3CR1^gfp+^ cells from isotype-treated and AFS98-treated mice was comparable (Supplementary Fig. 2C). Ly6C^high^ and Ly6C^low^ cells were both detectable with the latter expressing canonical patrolling monocyte markers CD43 and Treml4, as well as CD11c^9,13,36^ and being depleted in AFS98-injected mice (Supplementary Fig. 2C). We detected an accumulation of Ly6C^high^ monocytes in the spleen of AFS98-treated mice (Fig. 2D). Since CSF1R is expressed on bone marrow and spleen monocyte progenitors^39^, we aimed to determine whether these phenotypes could be due to altered monocyte generation or mobilization (Supplementary Fig. 2D). Bone marrow monocyte numbers were diminished (Fig. 2E), while neutrophils and myeloid progenitors were unaffected (Supplementary Fig. 2E, F).

Following prolonged treatment with an alternative anti-CSF1R blocking antibody, the loss of Ly6C^low^ monocytes was balanced by an increase in Ly6C^high^ monocytes suggesting inhibition of differentiation as a potential mechanism^23^. We tested this possibility in detail using the acute treatment regime with AFS98, using Treml4 as a marker of Ly6C^high^ monocyte commitment to the Ly6C^low^ subset^36^. Ly6C^high^ Treml4^-^ monocytes were not affected by AFS98 administration (Fig. 2F). However, the numbers of Ly6C^+^ Treml4^+^ monocytes were reduced, ultimately culminating in decreased counts of Ly6C^low^ Treml4^+^ monocytes (Fig. 2F). Together, these data provide further evidence that monocytopoiesis is CSF1R-independent^21^ whereas monocyte differentiation into the Ly6C^low^ subset could be affected by CSF1R blockade.

The decrease of blood Ly6C^+^ Treml4^+^ and Ly6C^low^ Treml4^+^ monocytes might also be resulting from an increased adherence to endothelial cells. The integrin CD11a plays a pivotal role in the interaction between circulating monocytes and blood vessels^8^. Treatment with a CD11a-blocking Ab increased the numbers of Ly6C^high^ and Ly6C^low^ monocytes detected in blood (Supplementary Fig. 2G, left panel). CSF1R expression on both monocyte subsets was decreased after CD11a blockade (Supplementary Fig. 2G, right panel). To investigate whether CSF1R blockade could facilitate endothelial cell adherence, we treated mice with AFS98 Ab (as described above) and injected a CD11a blocking Ab intravenously 5 min before culling. CD11a blockade in AFS98-treated mice did not restore the levels of blood Ly6C^+^ Treml4^+^ and Ly6C^low^ Treml4^+^ monocytes in comparison to control animals (Supplementary Fig. 2H). Indeed, blood monocyte subset diversity was comparable in mice treated solely with AFS98 or a combination of AFS98 and CD11a (Supplementary Fig. 2H). Similarly, to exclude the possibility that AFS98 affected extravasation, we performed a similar analysis in subcutaneous adipose tissue (scAT), a tissue in which we previously observed a robust monocyte contribution to the pool of tissue resident macrophages at steady-state. Because monocytes reside in peripheral tissues outside of blood vessels^40^, we injected control and AFS98-treated mice with an anti-CD45 FITC-conjugated Ab intravenously 5 min before culling to discriminate between blood (CD45i.v.^+^) and extravasated (CD45i.v.^-^) monocytes. In scAT, we observed the presence of blood- and tissue-residing monocytes (Fig. 2G, H). CSF1R blockade using AFS98 Ab decreased selectively the numbers of CD45i.v.^+^ monocytes (Fig. 2H). Of note, among CD45i.v^+^ monocytes, AFS98 treatment reduced the numbers of all monocyte subsets (Fig. 2I). While the numbers of scAT CD45i.v.^-^ monocytes were not affected by CSF1R blockade, the Ly6C^low^ Treml4^+^ subset was decreased (Supplementary Fig. 2I). Furthermore, AFS98 administration induced lower expression of CD206 on scAT CD45 i.v.^-^ Ly6C^high^ monocytes and a similar trend was observed regarding their F4/80 expression (Supplementary Fig. 2J). Since both CD206 and F4/80 are markers associated with the progressive acquisition of tissue residency by monocytes following their recruitment, these data suggested that CSF1R-signaling supported this process.

Taken together, these data revealed that CSF1R blockade reduced the numbers of blood Ly6C^+^ Treml4^+^ and Ly6C^low^ Treml4^+^ monocytes without affecting endothelial cell adherence or extravasation into peripheral tissues. Therefore, we sought to define whether the disappearance of Ly6C^low^ Treml4^+^ monocytes was due to a decreased survival rate or impaired generation by conversion of Ly6C^high^ cells.

### CSF1R regulates commitment of Ly6C^+^Treml4^+^ into Ly6C^low^ Treml4^+^ monocytes and survival of both subsets

**Fig. 3:**
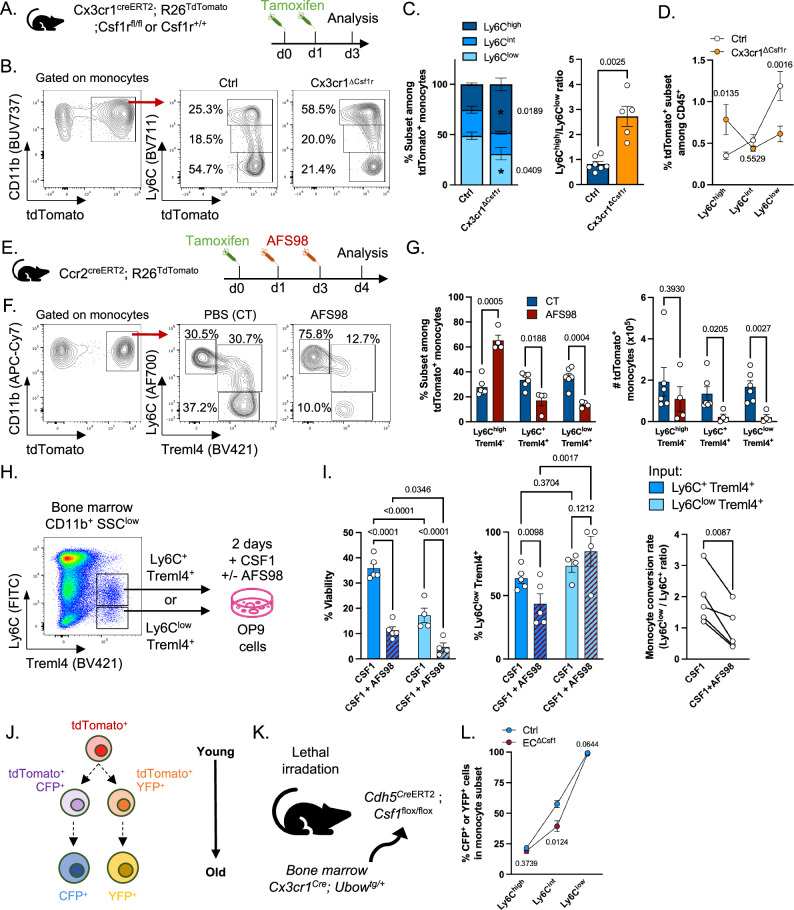
CSF1R blockade impairs Ly6C^high^ to Ly6C^low^ monocyte conversion. **A** Experimental scheme used to induce *Csf1r* deletion and tdTomato expression in Cx3cr1^CreERT2^ mice. Representative flow cytometry plots (**B**) and proportions of monocyte subsets among tdTomato^+^ monocytes and ratio between Ly6C^high^ tdTomato^+^ and Ly6C^low^ tdTomato^+^ monocytes (**C**) in Cx3cr1^CreERT2^; Csf1r^fl/fl^; R26^tdTomato^ (Cx3cr1^ΔCsf1r^) mice (*n* = 5) and Cx3cr1^CreERT2^; Csf1r^+/+^; R26^tdTomato^ controls (*n* = 7). Total monocytes were identified as in Fig. 2A. **D** Proportions of tdTomato^+^ monocyte subsets among total leukocytes from Cx3cr1^CreERT2^; Csf1r^fl/fl^; R26^tdTomato^ (Cx3cr1^ΔCsf1r^) mice (*n* = 5) and Cx3cr1^CreERT2^; Csf1r^+/+^; R26^tdTomato^ controls (*n* = 7). **E** Experimental scheme used to induce tdTomato expression and administer AFS98 in Ccr2^CreERT2^; R26^tdTomato^ mice. Representative flow cytometry plots (**F**) and quantification (**G**) of tdTomato+ monocytes in Ccr2^CreERT2^; R26^tdTomato^ mice following treatment with PBS (CT, *n* = 6) or AFS98 (*n* = 4). **H** Experimental scheme used to test monocyte survival and differentiation in vitro. **I** Viability (left), quantification (middle), and conversion rate (right) of monocyte subsets following in vitro culture with CSF1 and AFS98 and depending on whether Ly6C^+^ Treml4^+^ or Ly6C^low^ Treml4^+^ monocytes were cell-sorted from the bone marrow of *n* = 4 mice. **J** Schematic representation of labeling pattern in Ubow^tg/+^ mice following Cre recombinase-mediated recombination of the Ubow reporter. **K** Experimental scheme used to assess monocyte differentiation using Cx3cr1^Cre^; Ubow^tg/+^ bone marrow transplantation into control (WT) or Cdh5^CreERT2^; Csf1^flox/flox^ (EC^ΔCsf1^) recipient mice. **L** Proportions of cells expressing YFP or CFP within each monocyte subset in blood of Ctrl (*n* = 5) or EC^ΔCsf1^ (*n* = 5) chimeric mice. A two-way repeated measures ANOVA test was used in panel I (left and middle), without correction for multiple comparisons (uncorrected Fisher’s LSD). Two-way repeated measures ANOVA tests, corrected for multiple comparisons using Tukey’s multiple comparison tests, were used for statistical analysis in panels (**C**, **D**, **G**, and **L**). A two-sided unpaired Mann-Whitney test was used for statistical analysis in panel (**C**) (right). A two-sided paired *t* test was used for statistical analysis in panel (**I**) (right). Data are presented as mean values ± SEM. See also Supplementary Fig. 3. Source data are provided as a Source Data file.

The role of CSF1R in blood monocyte differentiation was explored further using genetic models. We generated a conditional deletion model (Cx3cr1^creERT2^; R26^tdTomato^; Csf1r^flox/flox^ (Cx3cr1^ΔCsf1r^)) (Fig. 3A and Supplementary Fig. 3A). Following tamoxifen (TAM) treatment, the proportions of Ly6C^high^ cells increased while the proportions of Ly6C^low^ cells decreased among tdTomato-labeled blood monocytes (Fig. 3B, C). In line, monocyte CSF1R-deficiency increased Ly6C^high^:Ly6C^low^ blood monocyte ratio (Fig. 3C). This approach validates our observations using AFS98 Ab, as after TAM treatment and consequent CSF1R deletion the proportions of Ly6C^low^ tdTomato^+^ monocytes diminished among total blood leukocytes in comparison to control animals (Fig. 3D).

To explore whether CSF1R signaling is involved in the conversion of Ly6C^high^ Treml4^-^ into Ly6C^+^ Treml4^+^ and Ly6C^low^ Treml4^+^ monocytes, we used Ccr2^CreERT2^; R26^tdTomato^ (Ccr2^tdTomato^) mice to inducibly label CCR2^+^ monocytes^38^ and track their differentiation into Ly6C^low^ cells in blood over time (Supplementary Fig. 3B). Ccr2^tdTomato^ mice were gavaged with TAM to induce tdTomato expression in Ly6C^high^ monocytes and we followed their fate following AFS98 treatment by focusing on tdTomato^+^ monocytes (Fig. 3E-F). Ly6C^high^ Treml4^-^ cells were the main subset among tdTomato^+^ monocytes from AFS98-treated mice, while they were the minor subset in control mice (Fig. 3F, G, left panel). The numbers of both Ly6C^+^ Treml4^+^ tdTomato^+^ and Ly6C^low^ Treml4^+^ tdTomato^+^ monocytes, but not Ly6C^+^ Treml4^-^ tdTomato^+^ cells, were diminished after AFS98 administration (Fig. 3G, right panel). This result indicated that CSF1R blockade could restrain the differentiation of Ly6C^high^ monocytes into Ly6C^+^ Treml4^+^ or affect the survival of Ly6C^+^ Treml4^+^ and Ly6C^low^ Treml4^+^ cells. We developed a complementary approach to investigate monocyte conversion in vivo. For this purpose, mice were intravenously injected with clodronate to deplete all blood monocytes. Clodronate-injected animals were administrated with AFS98 48 h later, a timepoint at which only Ly6C^high^ monocytes are detected in the circulation^41^ (Supplementary Fig. 3C). We investigated the appearance of Ly6C^+^ Treml4^+^ and Ly6C^low^ Treml4^+^ monocytes 48 h after AFS98 injection (Supplementary Fig. 3C). In agreement with the data presented in Fig. 3F, G, we found a decreased frequency of both Ly6C^+^ Treml4^+^ and Ly6C^low^ Treml4^+^ monocytes in AFS98 treated mice (Supplementary Fig. 3C). Quantitatively, the numbers of both Ly6C^+^ Treml4^+^ and Ly6C^low^ Treml4^+^ monocytes, but not Ly6C^high^ Treml4^-^ cells, were diminished after AFS98 administration (Supplementary Fig. 3D).

To decipher the relative contribution of Ly6C^+^ Treml4^+^ monocyte conversion and survival to the generation of Ly6C^low^ Treml4^+^ cells, we cell sorted both subsets and cultured them on OP9 feeder cells in the presence of recombinant CSF1 or a combination of CSF1 and AFS98 (Fig. 3H). AFS98 addition to cell culture affected the viability of both Ly6C^+^ Treml4^+^ and Ly6C^low^ Treml4^+^ monocytes (Fig. 3I, left panel) and decreased the conversion of Ly6C^+^ Treml4^+^ into Ly6C^low^ Treml4^+^ monocytes (Fig. 3I, middle panel), resulting in a decreased Ly6C^low^:Ly6C^+^ monocyte ratio (Fig. 3I, right panel). Thus, CSF1R controls both the survival of Ly6C^low^ monocytes and the differentiation of Ly6C^+^ Treml4^+^ into Ly6C^low^ Treml4^+^ cells.

To assess whether monocyte subsets half-life is affected by CSF1R signaling, we took advantage of Ubow^tg/+^ mice. In Ubow^tg/+^ mice, all cells initially express the fluorescent protein tdTomato, while YFP and CFP are not expressed. When a cell expresses Cre recombinase, it undergoes a single and irreversible recombination event. This recombination leads to the progressive and permanent loss of tdTomato expression and the random activation of either YFP or CFP, thereby allowing individual cells to be permanently labeled with one of these two fluorescent markers. Even if Cre recombinase continues to be expressed, no additional recombination events take place. Consequently, the cell retains the same fluorescent label throughout its entire lifespan (Fig. 3J)^42^. To target monocytes, we used Cx3cr1^Cre^; Ubow^tg/+^ mice. Bone marrow of these mice was extracted and transferred into lethally irradiated control mice (Ctrl) and Cdh5^CreERT2^; Csf1^fl/fl^ (EC^ΔCsf1^) allowing conditional deletion of *Csf1* in endothelial cells (Fig. 3K). A recent report demonstrated that EC^ΔCsf1^ mice almost entirely lack Ly6C^low^ blood monocytes after TAM administration^25^. The frequency of cells expressing YFP or CFP among Ly6C^int^ monocytes was lower in EC^ΔCsf1^ chimeric mice compared to control animals (Fig. 3L), suggesting that CSF1R stimulation by endothelial cell-derived CSF1 controls monocyte lifespan, and in particular that of the Ly6C^+^ Treml4^+^ subset.

Taken together, these observations demonstrated that CSF1R signaling affects both the differentiation of Ly6C^high^ Treml4^-^ into Ly6C^low^ Treml4^+^ monocytes and the survival of the latter subset.

### CSF1R sustains Ly6C^low^ monocytes in Csf1r^ΔFIRE^ mice

**Fig. 4:**
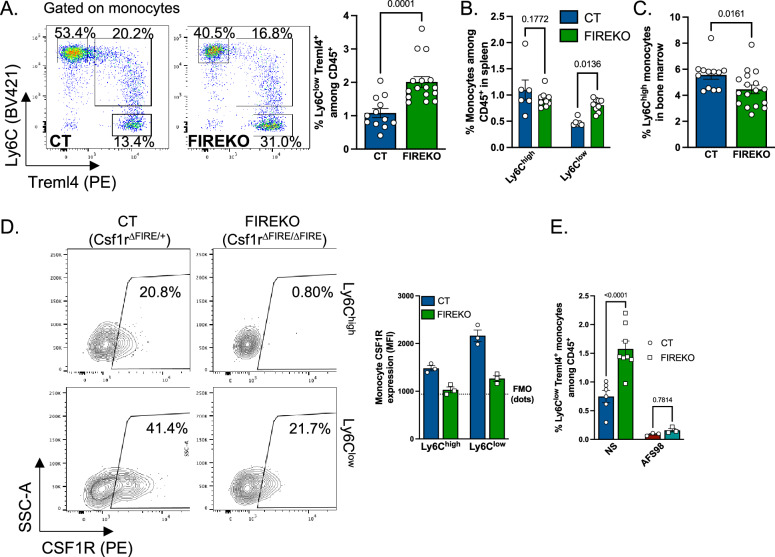
Ly6C^low^ monocytes rely on CSF1R signaling in FIREKO mice. **A** Proportions of Ly6C^low^ Treml4^+^ monocytes in blood from Csf1r^ΔFIRE/ΔFIRE^ (FIREKO, *n* = 16) mice or their littermate Csf1r^ΔFIRE/+^ controls (CT, *n* = 12). Data pooled from three independent experiments. **B** Proportions of Ly6C^high^ and Ly6C^low^ monocytes in spleen from FIREKO (*n* = 9) and CT (*n* = 6) mice. Data pooled from two independent experiments. **C** Proportions of Ly6C^high^ monocytes in bone marrow from FIREKO (*n* = 17) and CT (*n* = 12) mice. Data pooled from three independent experiments. **D** Representative flow cytometry plots (left) and quantification (right) or CSF1R expression by blood Ly6C^high^ and Ly6C^low^ monocytes from FIREKO (*n* = 3) and littermate CT (*n* = 3) mice. **E** Proportions of blood Ly6C^low^ monocytes in FIREKO and littermate CT mice at steady state (NS, *n* = 6 CT and *n* = 8 FIREKO) or following treatment with AFS98 (*n* = 3 CT and *n* = 3 FIREKO). Two-way repeated measures ANOVA tests were used in panels (**B** and **E**), without correction for multiple comparisons (uncorrected Fisher’s LSD). Two-sided unpaired Mann–Whitney tests were used for statistical analysis in panels (**A** and **C**). Data are presented as mean values ± SEM. Source data are provided as a Source Data file.

We conducted similar analyses in Csf1r^ΔFIRE/ΔFIRE^ (FIREKO) mice, which were shown to lack CSF1R expression in bone marrow progenitors and are unresponsive to CSF1 due to deletion of an enhancer within the *Csf1r* locus^26^. Csf1r^ΔFIRE/+^ mice, in which the mutation is not dosage compensated leading to 50% reduced CSF1R expression^26^, were used as controls (CT). Ly6C^low^ Treml4^+^ monocytes were enriched among total leukocytes in the blood of FIREKO compared to littermate CT mice, while Ly6C^high^ monocytes tended to decrease (Fig. 4A). A similar phenotype was observed in spleen from FIREKO mice (Fig. 4B), while their bone marrow monocytes were reduced (Fig. 4C). In the original description of these mice^26^, CSF1R expression was detectable on Ly6C^low^ monocytes, while no signal was detected on Ly6C^high^ monocytes. This finding is confirmed in Fig. 4D. To explore whether the remaining CSF1R expression on Ly6C^low^ monocytes could be functionally important, AFS98 Ab was injected in FIREKO and littermate CT mice. In both groups AFS98 Ab treatment completely depleted blood Ly6C^low^ monocytes, demonstrating the role of CSF1R signaling in this model as well (Fig. 4E).

### GMP- and MDP-derived Ly6C^low^ monocytes are differentially affected by CSF1R blockade

**Fig. 5:**
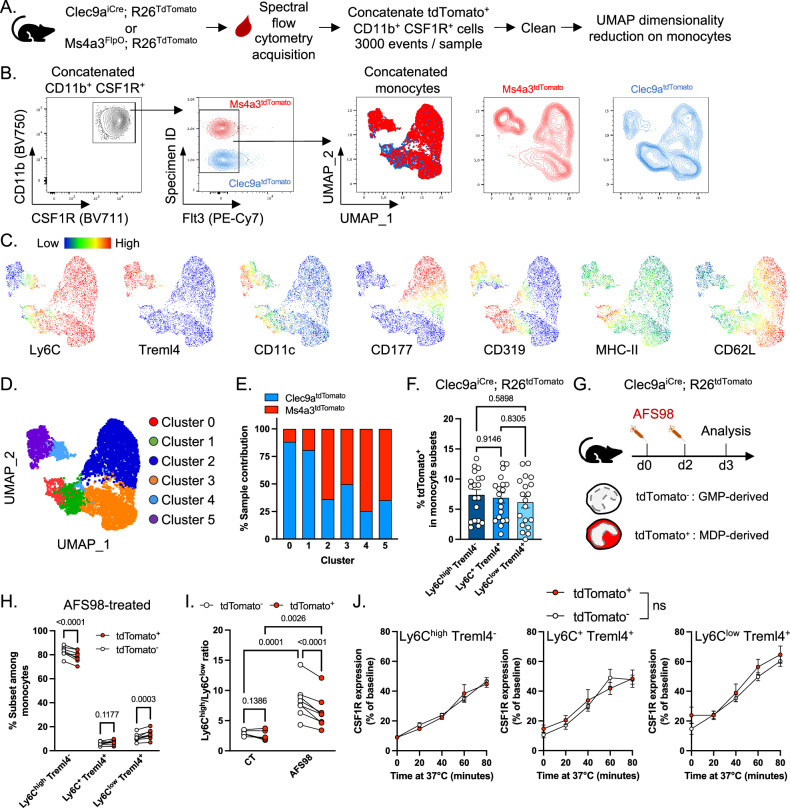
AFS98 treatment differentially affects GMP- and MDP-derived monocytes. Experimental scheme (**A**) and corresponding representative flow cytometry plots (**B**) used to compare the phenotype of tdTomato^+^ monocytes from Clec9a^iCre^; R26^LSL-tdTomato^ (Clec9a^tdTomato^, *n* = 9) and Ms4a3^FlpO^; R26^FSF-tdTomato^ (Ms4a3^tdTomato^, *n* = 4) mice. **C** UMAP representing of Ly6C, Treml4, CD11c, CD177, CD319, MHC-II, and CD62L expression by blood CD11b^+^ CD115^+^ tdTomato^+^ cells from Clec9a^tdTomato^ and Ms4a3^tdTomato^ mice analyzed by spectral flow cytometry. Unsupervised clustering of flow cytometry data using FlowSOM, generating 6 clusters (**D**) and quantification of sample contribution to these clusters (**E**). **F** Proportions of tdTomato^+^ cells among blood monocyte subsets from *n* = 19 Clec9a^tdTomato^ mice. **G** Experimental scheme used to test the impact of CSF1R blockade on MDP- and GMP-derived monocytes using Clec9a^tdTomato^ mice. **H** Proportions of monocyte subsets among tdTomato^+^ and tdTomato^-^ monocytes from AFS98-treated Clec9a^tdTomato^ mice (*n* = 8). **I** Ratio of blood Ly6C^high^/Ly6C^low^ monocytes among tdTomato^+^ and tdTomato^-^ cells from Clec9a^tdTomato^ mice administered PBS (CT, *n* = 6) or AFS98 (*n* = 8). **J** Analysis of CSF1R turnover in blood monocytes from Clec9a^iCre^; R26^tdTomato^ mice. *n* = 9 mice for Ly6C^high^ Treml4^-^ and Ly6C^low^ Treml4^+^ cells, and *n* = 8 mice for Ly6C^+^ Treml4^+^ cells. tdTomato^+^ and tdTomato^-^ cells are matched. Data pooled from three experiments. A repeated-measures one-way ANOVA with Tukey’s multiple comparison test was used for statistical analysis in panel (**F**). A two-way repeated measures ANOVA test was used in panel I, without correction for multiple comparisons (uncorrected Fisher’s LSD). Two-way repeated measures ANOVA tests, corrected for multiple comparisons using Tukey’s multiple comparison tests, were used for statistical analysis in panels (**H** and **J**). Data are presented as mean values ± SEM. See also Supplementary Fig. 4. Source data are provided as a Source Data file.

The vast majority of blood monocytes are generated from GMPs^2^, but a subpopulation derived from MDPs can be distinguished by surface markers^43^. We identified and validated Clec9a^iCre^ mice as a tool to target and analyze MDP-derived monocytes^44^. We used Clec9a^iCre^; R26^LSL-tdTomato^ (Clec9a^tdTomato^) mice to label MDP-derived monocytes and Ms4a3^FlpO^; R26^FSF-tdTomato^ ^45^ (Ms4a3^tdTomato^) mice to tag GMP-derived monocytes. We compared the phenotype of tdTomato^+^ monocytes from each strain using spectral flow cytometry analysis and investigated the expression of multiple markers associated with monocyte subset diversity or a specific ontogeny to validate these models (Fig. 5A). CD11b^+^ CD115^+^ tdTomato^+^ monocytes from Ms4a3^tdTomato^ and Clec9a^tdTomato^ mice were concatenated, Flt3^+^ cells were excluded to avoid contamination by dendritic cell precursors, and Uniform Manifold Approximation and Projection (UMAP) was used to perform dimensionality reduction on the remaining cells (Fig. 5B). Monocytes from Ms4a3^tdTomato^ and Clec9a^tdTomato^ mice showed a distinct distribution on the UMAP projection (Fig. 5B). We investigated the expression of markers recently proposed to be enriched in GMP-derived (CD177, CD62L) or MDP-derived (CD319, MHC-II) monocytes^43^ and validated that these were positively associated with tdTomato^+^ monocytes from Ms4a3^tdTomato^ and Clec9a^tdTomato^ mice, respectively (Fig. 5C). We next performed unsupervised clustering using FlowSOM and visualized 6 major clusters (labeled from 0 to 5) (Fig. 5D). Clusters 0 and 1, corresponding to CD319^+^ MHC-II^+^ and CD319^+^ MHC-II^-^ cells (Fig. 5C), derived almost entirely from the Clec9a^tdTomato^ samples (Fig. 5E). In contrast, cells from the Ms4a3^tdTomato^ samples were enriched in clusters 2, 4 and 5 (Fig. 5E). Of note, cluster 2 cells highly expressed CD177 (Fig. 5E). Since clusters 4 and 5 respectively corresponded to Ly6C^+^ Treml4^+^ and Ly6C^low^ Treml4^+^ monocytes, we sought to quantify tdTomato labeling in monocytes from Clec9a^tdTomato^ mice using a conventional approach. We found that on average 6% of monocytes were tdTomato^+^ in all three monocyte populations, suggesting that MDP-derived Ly6C^high^ Treml4^-^ monocytes could generate Ly6C^low^ Treml4^+^ cells similarly to tdTomato^-^ GMP-derived cells (Fig. 5F), as suggested previously using surface markers^43^.

We then administered AFS98 Ab to Clec9a^tdTomato^ mice to test whether MDP-derived monocytes also relied on CSF1R (Fig. 5G). CSF1R blockade had no significant impact on the proportions of MDP-derived cells among bone marrow and spleen Ly6C^high^ monocytes (Supplementary Fig. 4A). In blood, we observed a partial depletion of Ly6C^+^ Treml4^+^ and Ly6C^low^ Treml4^+^ monocytes among both the tdTomato^+^ (MDP-derived) and the tdTomato^-^ (GMP-derived) monocyte fractions upon AFS98 administration (Supplementary Fig. 4B). However, when comparing tdTomato^+^ to tdTomato^-^ monocyte subsets of AFS98-treated animals, we found a higher abundance of Ly6C^low^ Treml4^+^ monocytes among the tdTomato^+^ fraction (Fig. 5H). Accordingly, while CSF1R blockade led to an increased Ly6C^high^/Ly6C^low^ ratio among both tdTomato^+^ and tdTomato^-^ monocytes, this ratio remained lower in AFS98-treated tdTomato^+^ monocytes compared to their tdTomato^-^ counterpart (Fig. 5I). Finally, the relative resistance of MDP-derived monocytes to AFS98 treatment was not a direct consequence of a differential CSF1R turnover as CSF1R reappearance at the cell surface was comparable between tdTomato^-^ GMP-derived and tdTomato^+^ MDP-derived monocytes (Fig. 5J).

Overall, our results indicate that while CSF1R signaling regulates both GMP- and MDP-derived monocytes, the latter are more resilient to CSF1R blockade.

### CSF1R signaling and glucose metabolism interconnect through the hexosamine biosynthetic pathway

**Fig. 6:**
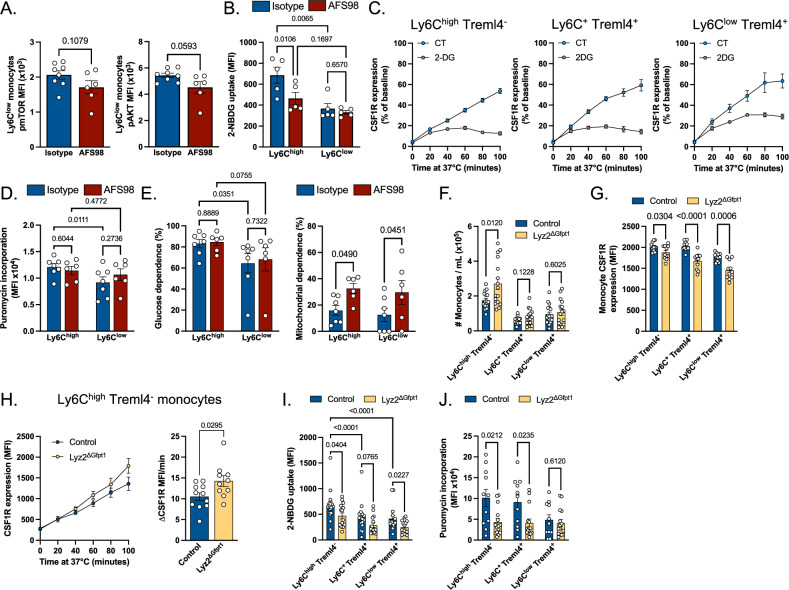
Inter-regulations of CSF1R and glucose metabolism control monocyte homeostasis. **A** Quantification of mTOR and AKT phosphorylation in blood Ly6C^low^ monocytes from mice treated with AFS98 (*n* = 6) or isotype control (*n* = 8). **B** 2-NBDG incorporation in blood Ly6C^high^ and Ly6C^low^ monocytes following CSF1R blockade or isotype control administration (*n* = 5 mice per group). **C** Time-course analysis of surface CSF1R expression on blood monocyte subsets from wild-type mice following incubation at 37 °C to allow receptor recycling, in the presence (*n* = 7) or absence (*n* = 11) of 2-DG. Data pooled from two independent experiments. **D** Quantification of puromycin incorporation in blood Ly6C^high^ and Ly6C^low^ monocytes from mice treated with AFS98 (*n* = 6) or isotype control (*n* = 7). Data pooled from two independent experiments. **E** Metabolic profile of blood monocytes from mice treated with AFS98 (*n* = 7) or isotype control (*n* = 6), measured using SCENITH. Quantification of blood monocyte subsets (**F**) and their CSF1R expression (**G**) in control (*n* = 17) and Lyz2^ΔGfpt1^ (*n* = 16) mice. Data pooled from five independent experiments. **H** Time-course analysis of surface CSF1R expression on blood Ly6C^high^ Treml4^-^ monocytes from control (*n* = 11) and Lyz2^ΔGfpt1^ (*n* = 10) mice following incubation at 37 °C to allow receptor recycling. Quantification as MFI per minute (right plot) is shown for each individual mouse. Data pooled from three independent experiments. **I** Analysis of 2-NBDG uptake by blood monocyte subsets from control (*n* = 16) and Lyz2^ΔGfpt1^ (*n* = 15) mice. Data pooled from four independent experiments. **J** Quantification of puromycin incorporation by blood monocyte subsets from control (*n* = 11) and Lyz2^ΔGfpt1^ (*n* = 15) mice. Data pooled from three experiments. Two-way repeated measures ANOVA tests were used in panels (**B**, **D**, and **E**), without correction for multiple comparisons (uncorrected Fisher’s LSD). Two-way repeated measures ANOVA tests, corrected for multiple comparisons using Tukey’s multiple comparison tests, were used in panels (**F**, **G**, **I** and **J**). A two-sided unpaired Mann-Whitney test was used for statistical analysis in panel A. A two-sided unpaired Mann–Whitney was used for statistical analysis in panel (**H**). Data are presented as mean values ± SEM. See also Supplementary Fig. 5. Source data are provided as a Source Data file.

In comparison to closely related myeloid cells such as macrophages and dendritic cells, data on the metabolic configuration of blood monocyte subsets in mice and humans are scarce^46^. In monocyte-derived macrophages, CSF1 binding to CSF1R supports metabolic reprogramming towards glycolysis^27,47^. Therefore, we investigated intracellular metabolism pathways in blood monocytes after AFS98 Ab treatment. CSF1R blockade tended to inhibit AKT and mTOR phosphorylation in blood Ly6C^low^ but not Ly6C^high^ monocytes, reflecting an altered metabolic state (Fig. 6A and Supplementary Fig. 5A). While glucose uptake (quantified by uptake of the fluorescent glucose analog 2-NBDG) was lower in Ly6C^low^ compared to Ly6C^high^ monocytes in isotype-treated mice, CSF1R blockade decreased glucose uptake in Ly6C^high^ monocytes (Fig. 6B). On the other hand, inhibiting glucose metabolism with 2-deoxyglucose (2-DG) led to reduced surface CSF1R levels in our re-expression assay (Fig. 6C). These findings are consistent with our previous report that Glut-1 inhibition leads to a rapid downregulation of CSF1R followed by disappearance of monocytes in vivo^28^. Furthermore, they highlight inter-regulations between glucose metabolism and CSF1R expression that regulate monocyte subsets differentiation and survival.

We next applied the recently described SCENITH approach to define monocyte metabolic configuration upon CSF1R blockade^48^. Although presenting several limitations, this approach monitors intracellular metabolism at a single-cell level resolution. Ly6C^high^ monocytes incorporated more puromycin than Ly6C^low^ cells (Fig. 6D), reflecting higher metabolic and ribosomal activities. Puromycin incorporation was highly dependent on glucose intracellular metabolism^28^ independently of isotype or AFS98 treatment (Fig. 6E and Supplementary Fig. 5B). Ly6C^high^ monocytes showed a higher glucose dependence compared to the Ly6C^low^ subset (Fig. 6E). However, CSF1R blockade sensitized monocytes to oligomycin treatment, reflecting a higher mitochondrial dependence compared to control cells (Fig. 6E and Supplementary Fig. 5B). Consistent with evidence that CSF1R signaling is active in monocytes in FIREKO mice, despite low receptor expression, monocyte metabolism was not significantly affected by the homozygous mutation (Supplementary Fig. 5C). CSF1R signaling thus appears to modulate monocyte intracellular metabolism and dependency on glucose metabolism or mitochondrial oxidative phosphorylation.

Glucose intracellular metabolism integrates three pathways: glycolysis, the pentose phosphate pathway (PPP), and the HBP. Our previous data using genetic models suggested that glycolysis and PPP are not essential for CSF1R regulation^28^. However, such a tool was not available to analyze the role of HBP in CSF1R expression and studies regarding this pathway heavily relied on pharmacological inhibitors, the most applied being tunicamycin. GFPT1 is the first and rate-limiting enzyme in HBP. Recently, GFPT1^fl/fl^ mice were generated and the deletion of this enzyme affected T cell thymic generation^49^. To investigate the role of HBP in monocyte biology, with a particular focus on CSF1R expression, we generated Lyz2^Cre^; Gfpt1^fl/fl^ mice (named Lyz2^ΔGfpt1^) (Supplementary Fig. 6A). Recombination efficiency in ear biopsies was confirmed by PCR on genomic DNA, permitting distinction of the Gfpt1^flox^ allele before (tm1c) and after (tm1d) recombination (Supplementary Fig. 6A-B). Western blot analysis demonstrated the complete loss of GFPT1 protein in Lyz2^ΔGfpt1^ bone marrow-derived macrophages (BMDM) in comparison to control cells (Supplementary Fig. 6C). Because Lyz2^cre^ efficiency is higher in macrophages in comparison to monocytes, we next investigated whether we could detect similar deletion in monocytes. We cell-sorted splenic Ly6C^high^ Treml4^-^, Ly6C^+^ Treml4^+^ and Ly6C^low^ Treml4^+^ monocytes and performed a PCR analysis as described above (Supplementary Fig. 6D). Our data confirmed that Lyz2^cre^ efficiently ablated GFPT1 expression in all monocyte subsets (Supplementary Fig. 6D). The deletion efficiency varied from nearly 60% in Ly6C^high^ Treml4^-^ monocytes to 70% in Ly6C^+^ Treml4^+^ and Ly6C^low^ Treml4^+^ cells (Supplementary Fig. 6D). These data are in agreement with previous reports demonstrating higher, but yet incomplete, Lyz2^Cre^ efficiency in Ly6C^low^ monocytes in comparison to Ly6C^high^ cells^50^.

Among blood immune cells, Lyz2^cre^ targets mainly neutrophils and monocytes. Blood neutrophil and total monocyte numbers were not affected by GFPT1 deletion (Supplementary Fig. 6E-F). When analysing monocyte sub-populations, we observed increased numbers of Ly6C^high^ Treml4^-^ monocytes in Lyz2^ΔGfpt1^ mice (Fig. 6F). CSF1R expression was decreased in all GFPT1-deficient monocyte subsets (Fig. 6G). We next aimed to define whether the reduced cell surface CSF1R expression in GFPT1-deficient monocytes was due to altered intracellular cycling and membrane export. To our surprise, we found faster CSF1R recycling in GFPT1-deficient monocytes in comparison to control cells (Fig. 6H and Supplementary Fig. 6G-H), suggesting that compensatory mechanisms could be at play.

We next investigated whether GFPT1-deficiency affected intracellular metabolism. Seahorse analysis of BMDM indicated marginally increased basal respiration in GFPT1-deficient compared to control BMDM (Supplementary Fig. 6I). Ex vivo, we found that monocyte uptake of 2-NBDG was diminished in cells lacking GFPT1 (Fig. 6I). Consequently, we observed that puromycin incorporation was also diminished in GFPT1-deficient Ly6C^high^ Treml4^-^ and Ly6C^+^ Treml4^+^ monocytes, reflecting on a reduced ribosomal activity (Fig. 6J). Overall, these findings suggest that GFPT1 deficiency leads to reduced metabolic activity and activation of compensatory yet less efficient CSF1R expression mechanisms, resulting in an imbalance among blood monocyte subsets.

These results indicate that the HBP pathway contributes to the glucose-dependent regulation of CSF1R expression and monocyte homeostasis.

### Receptor tyrosine kinases regulate human monocyte metabolism and survival

**Fig. 7:**
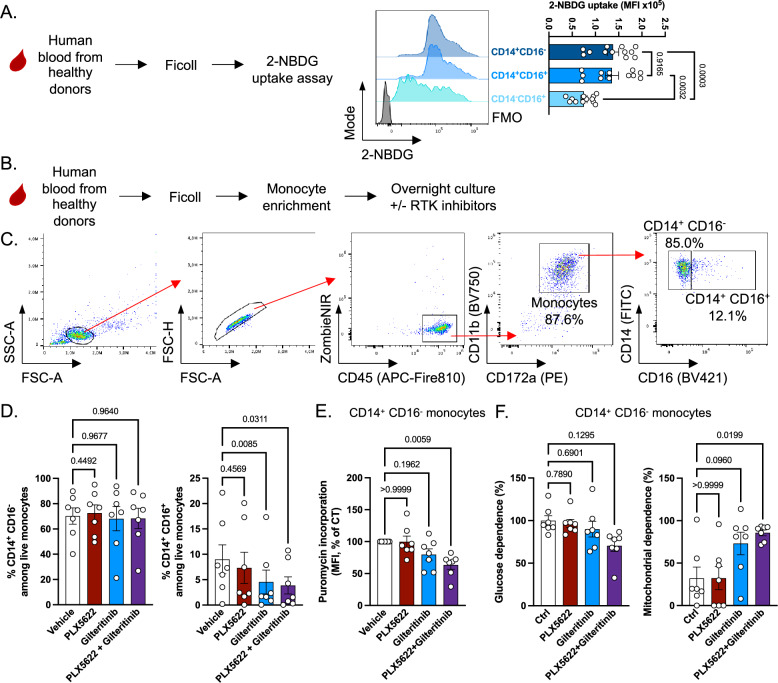
Receptor tyrosine kinases control monocyte metabolic activity in humans. **A** Quantification of 2-NBDG uptake by blood monocyte subsets from healthy donors (*n* = 12 donors). Experimental scheme (**B**) and gating strategy (**C**) used to analyze human blood monocytes following an overnight incubation with the RTK inhibitors PLX5622 and Gilteritinib. **D** Proportions of classical (left) and intermediate (right) monocytes among total live monocytes following overnight culture with vehicle, PLX5622, Gilteritinib or both inhibitors. Quantification of puromycin incorporation (**E**) and metabolic profile (**F**) of blood classical monocytes following overnight culture with vehicle, PLX5622, Gilteritinib or both inhibitors. Data in panels (**D**–**F**) are pooled from 7 independent experiments, each performed on monocytes enriched from a pool of healthy donors blood. Repeated measures one-way ANOVA with Tukey’s multiple comparison tests were used for statistical analysis. Data are presented as mean values ± SEM. Source data are provided as a Source Data file.

We next sought to test the relevance of our observations using human cells. We quantified glucose uptake by human monocytes from healthy donors using 2-NBDG and found that glucose transport was more active in CD14^+^ CD16^-^ and CD14^+^ CD16^+^ compared to CD14^-^CD16^+^ monocytes (Fig. 7A). SCENITH analyses suggested that human classical monocytes are highly glycolytic while mitochondrial metabolism is also active in non-classical monocytes^48^. We previously reported a similar role of glucose intracellular metabolism on CSF1R expression in murine and human monocytes^28^. Together, these results suggest that our observations in mice could be translated to human monocytes.

In humans, the CSF1R blocking Ab Axatilimab demonstrated promising results in chronic graft-versus-host disease patients^51^. Yet, in clinical practice receptor tyrosine kinase inhibitors targeting CSF1R or FLT3 are most often administered to patients due to the role of these two RTKs on human monocyte biology^20,52^. While Axatilimab and RTK inhibitors decrease the numbers of CD16^+^ non-classical monocytes^51^, their effects on CD14^+^ classical monocytes are not fully understood. Thus, we aimed to investigate whether CSF1R and FLT3 inhibitors could modulate human classical monocyte intracellular metabolism. Blood monocytes from healthy donors were enriched using negative selection and were incubated with PLX5622 (CSF1R inhibitor), Gilteritinib (FLT3 inhibitor) or a combination of both and monocyte intracellular metabolism was assessed using the SCENITH approach^48^ (Fig. 7B). Following overnight culture, CD14^+^ CD16^-^ monocytes were the main subset and CD14^+^ CD16^+^ monocytes were much less abundant (Fig. 7C, D). Moreover, treatment with Gilteritinib led to diminished abundance of CD14^+^CD16^+^ monocytes (Fig. 7D, right panel). While treatment with PLX5622 or Gilteritinib alone had no or little effect on puromycin incorporation, treatment with a combination of both inhibitors led to a clear reduction of ribosomal activity (Fig. 7E). PLX5622 had no detectable effect on glucose and mitochondrial intracellular metabolism in monocytes (Fig. 7F). Gilteritinib increased monocyte mitochondrial dependence and a combination of PLX5622 and Gilteritinib even further amplified this effect (Fig. 7F). Taken together these data indicated that blocking the activity of both CSF1R and FLT3 reprograms classical monocyte metabolic configuration from glucose to mitochondrial dependence.

## Discussion

The use of blocking Ab-based strategies and genetic models aiming at investigating the role of CSF1R on blood monocyte subsets survival and generation have generally favored the view that there is a selective impact on the non-classical subset^22–24,26,29–31^. Non-classical monocytes in rats, the dominant subset in this species, also rely on CSF1R for their differentiation^53–55^. A humanized anti-CSF1R antibody, axatilimab, has been approved for the treatment of chronic graft-versus-host disease^51^. Treatment involves intermittent administration every 2 weeks, which leads to cyclical loss and recovery of CD16^+^ non-classical monocytes. Patients with dominant CSF1R coding mutations have a selective loss of non-classical monocytes^56^. While the effect of CSF1R blockade on mouse Ly6C^low^ monocytes appears consistent and clear, the underlying molecular and cellular mechanisms leading to the decrease of these cells were not well-defined.

Our analysis indicates that CSF1R signaling is required for monocyte differentiation and the survival of Ly6C^int^ and Ly6C^low^ monocytes. The differentiation of mouse monocyte subsets involves complex interactions between several transcription factors. Monocyte development strictly requires Zeb2, which is regulated by C/EBP transcription factors^57^. The transcription factor C/EBPα is required for the generation of Ly6C^high^ monocytes, but surprisingly the effect C/EBPα loss is bypassed to give rise directly to Ly6C^low^ monocytes^58^. Ly6C^low^ monocyte development involves Notch signaling and depends on RBP-J, Nr4a1 (Nur77), C/EBPβ, BCL6, and IRF2^58–62^. Several of these transcription factors bind to multiple regulatory elements in the *Csf1r* locus, whilst exclusively IRF8 binds to FIRE^63^ and IRF8 regulates monocytopoiesis^64–66^.

A recent study demonstrated that endothelial cell derived CSF1 is required to sustain blood Ly6C^low^ monocytes in a CX3CR1-dependent manner^25^. Ly6C^low^ monocytes in Tie2^Cre^; Csf1^fl/fl^ mice are almost completely absent from the blood circulation^25^. We confirmed that monocyte conversion depends, at least partially, on CSF1 produced by endothelial cells. We observed decreased Ly6C^low^ monocyte frequencies in Cx3cr1^CreERT2^; R26^tdTomato^; Csf1r^flox/flox^ mice (Fig. 3A). In this model, we also found an increased proportion of Ly6C^high^ monocytes consistent with an impact on monocyte conversion. Importantly, the lower numbers of Ly6C^int^ and Ly6C^low^ blood monocytes in AFS98 Ab-treated mice were not due to an increased adhesion to endothelial cells (Supplementary Fig. 2H). Depletion or dysregulation of CSF1R-dependent macrophages in any organ could increase extravasation of Ly6C^high^ monocytes, reducing their effective half-life in blood and decreasing their differentiation into the Ly6C^low^ subset. This hypothesis could be tested, for example, by targeting intestinal macrophages, which are continuously replaced by monocytes and are CSF1-dependent^67^.

In view of the impact of CSF1R inhibition, the increased proportion of Ly6C^low^ monocytes in FIREKO mice compared to littermate controls was unexpected (Fig. 4). Previous evidence indicated CSF1R was almost undetectable in blood monocytes from these mice. However, we detected residual CSF1R expression on Ly6C^low^, but not Ly6C^high^ blood monocytes in FIREKO mice. This could be due to faster CSF1R recycling on Ly6C^low^ compared to Ly6C^high^ monocytes. The selective depletion of the Ly6C^low^ subset in FIREKO mice treated with AFS98 confirmed that despite their low CSF1R expression, the Ly6C^low^ subset remains CSF1/CSF1R-dependent. Ly6C^high^ monocyte depletion (by administration of the CCR2-depleting MC-21 Ab) was reported to increase the lifespan of Ly6C^low^ monocytes^34^. This was paralleled by decreased detection of CSF1R on the remaining Ly6C^low^ monocytes^34^. We suggest that a similar mechanism underlies regulation in the FIREKO mice; the absence of the receptor in Ly6C^high^ cells increases the availability of the ligand to the Ly6C^low^ cells and increases their half-life. This conclusion leaves open the question of precisely how *Csf1r* transcription is initiated to enable expression in the absence of FIRE. More generally, the results also call into question the proposed role of CSF1 in the generation and maintenance of monocytes in bone marrow^68^. Another regulatory element, Csf1r upstream regulatory element A (CUREA), was recently shown to regulate CD177^+^ GMP-derived monocytes without altering CSF1R expression in monocytes^69^. The ontogeny of the remaining monocytes in FIREKO mice remains to be established.

Our data revealed that CSF1R membrane export is quite similar in GMP- and MDP-derived monocytes (Fig. 5). However, GMP-derived Ly6C^low^ blood monocytes were more sensitive to CSF1R blockade. MDP-derived monocytes display higher membrane MHC-II expression. A seminal study proposed that CSF2 triggers MHC-II expression on myeloid cells^70^. Thus, one could expect that MDP-derived blood monocytes could be partially depending on CSF2, thus being less responsive to CSF1R ablation or blockade.

Previous studies demonstrated that *Flt3l*^–/–^ mice display a more severe immune cell phenotype in comparison to *Flt3*-deficient animals^71,72^. In *Flt3*^–/–^, but not in *Flt3l*-deficient mice, CSF1R signaling compensated for FLT3 loss, suggesting that in the absence of one RTK another one could functionally compensate and restore phosphorylation of downstream targets^71^. An additional report using *Flt3*^–/–^*Csf1r*^–/–^ mice established a crosstalk between tissue resident macrophages and DCs^73^. A recent report demonstrated that humans bearing loss-of-function *FLT3L*-mutations displayed monocytopenia while neutrophil numbers were only moderately decreased^52^. These data suggest that human monocytes display higher FLT3L-dependence in comparison to mouse monocytes^52^. The underlying molecular patterns leading to this observation remain to be defined and might explain crucial parts of monocyte biology that differ among species.

CSF1R signaling regulates glucose uptake and metabolism in monocytes (Fig. 6)^28^. We showed using genetic models and pharmacological inhibitors, that while PFKFB3-dependent glycolysis and the pentose phosphate pathway were not essential for monocyte CSF1R expression and numbers, blocking the hexosamine biosynthetic pathway with tunicamycin reduced CSF1R expression on blood monocytes^28^. In the current study, we generated and validated a genetic model for depletion of GFPT1 in monocytes and found that CSF1R expression was decreased in all blood monocyte subsets, leading to a modulation of blood monocyte subsets proportions. Of note the numbers of Ly6C^high^ blood monocytes selectively increased in Lyz2^ΔGfpt1^ mice in comparison to littermate controls (Fig. 6). This was paralleled by a faster CSF1R export to the cell membrane in those cells. Yet their basal membrane CSF1R expression was lower compared to control Ly6C^high^ blood monocytes. The glycosylation pattern of CSF1R might be altered on specific sites and this could impact on receptor intracellular trafficking, membrane stabilization and ligand binding.

Taken together, this and the previous studies suggest that downstream metabolism of glucose by any of the three alternative pathways does not explain the effect of glucose transport inhibition. An alternative possibility is that the generation of glucose-6-phosphate (G6P) per se is essential. 2-Deoxyglucose, which competitively inhibits hexokinase, induces ER stress and an unfolded protein response in human monocytes^74^. There is also a close regulatory relationship between G6P, glycolytic enzymes and the activity of the vacuolar ATPase^75^, which in turn has been implicated in the control of CSF1R trafficking from the Golgi^76^. These alternative explanations for the essential role of glucose uptake in monocyte differentiation warrant further investigation.

## Methods

The present research complies with all relevant ethical regulations. Human participants provided written informed consent through the Etablissement Français du Sang. All animal procedures were approved by local ethical committees as listed below.

### Experimental models

#### Mouse models

Wild-type C57BL/6 J mice were purchased from Charles River or Janvier Labs. Cx3cr1^GFP^ (B6.Cg-Ptprca Cx3cr1^tm1Litt/LittJ^, Jax #008451), Clec9a^iCre^ (B6J.B6N(Cg)-*Clec9a*^*tm2.1(icre)Crs*^/J, Jax #025523), Lyz2^Cre^ (B6.129P2-Lyz2tm1(cre)Ifo/J, Jax#004781) and R26^TdTomato^ (B6.Cg-Gt(ROSA)26Sor^tm9(CAG-tdTomato)Hze^/J, Jax #007909) mice used in this study were maintained under C57BL/6J background and originally purchased from the Jackson Laboratory. Ccr2^CreERT2^ (C57BL/6NTac-Ccr2^tm2982(T2A−Cre7ESR1-T2A-mKate2)^) mice^38^ were kindly provided by Dr. Burkhard Becher. Cx3cr1^CreERT2^ mice were reconstituted from cryopreserved sperm provided by Steffen Jung^34^ to Gwendalyn J. Randolph. Csf1r^ΔFIRE^ ^26^, Csf1^flox/flox^ ^77^ and Cdh5^CreERT2^ ^78^ mice were provided by Dr. David Hume, Dr. Sherry Abboud Werner and Dr. Ralf Adams (respectively) to Dr. Marc Bajénoff. Gfpt1 floxed mice (57BL/6N-Gfpt1<tm1c(EUCOMM)Wtsi > /H) were obtained from Mary Lyon Center (MRC, Harwell, UK). Ubow mice were previously described^42^. Cx3cr1^Cre^ (Tg(Cx3cr1-cre)MW126Gsat/Mmucd) mice were obtained from the GENSAT transgenic projet. Ms4a3^FlpO^,^45^ and R26^FSF-tdTomato^ (B6.Cg-Gt(ROSA)26Sortm65.2(CAG-tdTomato)Hze/J, Jax#032864) mice were kindly shared by Dr. Florent Ginhoux and Dr. Elvira Mass. Experimental and control animals were co-housed, and littermate controls were used as often as possible. Animals of mixed sex and similar age were used within each cohort. Animals were used between the ages of 8 and 16 weeks throughout the study. Since we did not observe any sex-specific phenotypes, we decided to group data from male and female mice together in experiments where mice of both sexes were used. Mice were bred and housed in the Mediterranean Center of Molecular Medicine facility (INSERM U1065, Université Côte d’Azur), the PEMED facility (Pasteur campus, Université Côte d’Azur), the Washington University in Saint Louis animal facility, the University of Minnesota Medical School Research Animal Resources facility, or the Center d’Immunologie de Marseille Luminy facility. An ambient temperature of ~20-23 °C was maintained, with a 12/12-h light/dark cycle and food available ad libitum. Animals were euthanized by cervical dislocation. Animal protocols required for experimentation other than organ collection were authorized by the French Ministry of Higher Education and Research upon approval of the local ethical committee (CIEPAL Azur) at Université Côte d’Azur, and by the Institutional Animal Care and Use Committee (IACUC) at Washington University in Saint Louis and University of Minnesota Medical School.

#### Human cells

Human peripheral blood mononuclear cells were obtained from healthy donors after informed consent through the Etablissement Français du Sang, the French national agency for blood collection (convention numbers 13-PP-11 and NQNA04). Biological sex and age were not considered in the study design as this information was not collected.

### Bone marrow chimeras

To generate regular bone marrow chimeras, mice were subjected to full-body irradiation (9 Gy) and reconstituted with at least ∼3 × 10^6^ total bone marrow cells prepared using standard procedures. Mice were analyzed 2 months following bone marrow transfer.

### Genotyping

DNA was extracted from ear biopsies by incubation with 25 mM NaOH/0.2 mM EDTA for 60 min at 105 °C, followed by addition of an equal volume of 40 mM Tris HCL (pH 5.5). DNA was amplified by PCR using DreamTaq Green PCR Master Mix (2X) (Thermo Scientific). PCR products were visualized on a 3% agarose gel. A list of primers used for genotyping is provided in Supplementary Table 1.

For determination of GFPT1 deficiency in Ly6C^high^ Treml4^-^, Ly6C^+^ Treml4^+^, and Ly6C^low^ Treml4^+^ monocytes, these different populations were FACS-sorted using a BD FACSAria II on spleen cell homogenates from GFPT1^ΔMye^ and control littermates prior to using the above-described genotyping protocol directly on the sorted cell populations.

### In vivo treatments

#### CSF1R blockade

Mice were injected intra-peritoneally with 500 μg anti-CD115 blocking antibody (AFS98, BioXCell cat#BE0213), isotype control (Rat IgG2a, BioXcell cat#BE0089) or PBS as indicated in the corresponding figure legends.

#### Clodronate treatment

To deplete blood monocytes, mice were injected intravenously with 100 μL clodronate liposomes (Liposoma).

#### CD11a blockade

To detach endothelium-associated leukocytes, mice were injected intravenously with 100 μg anti-CD11a blocking antibody (M17/4, BioXCell cat#BE0006), isotype control (Rat IgG2a, BioXcell cat#BE0089) diluted in 100 μL PBS or PBS only as indicated in the corresponding figure legends.

#### CD45i.v. labeling

To distinguish blood from tissue monocytes, mice were injected intravenously with 1 μg FITC-conjugated anti-CD45 diluted in 100 μL PBS. Blood and tissues were collected 5 min later.

#### Tamoxifen treatment

Cx3cr1^creERT2^ mice were treated with tamoxifen dissolved in corn oil (20 mg/mL, 100 µL/mouse/administration) by oral gavage as often as indicated in Fig. 3A. Ccr2^creERT2^ mice were treated with tamoxifen dissolved in corn oil (20 mg/mL, 200 µL/mouse/administration) by oral gavage as indicated in Fig. 3E.

Cdh5^creERT2^ and control mice were fed a tamoxifen diet (Safe, diet E8220 Version 0366) for 4 weeks following irradiation and reconstitution with Cx3cr1^Cre/+^; Ubow^tg/+^ bone marrow. They were then fed standard chow diet for another 4 weeks before their blood monocytes were analyzed.

### Ex vivo cell treatments

#### Murine cells

Blood was collected in heparinized tubes by submandibular bleeding. Whole blood was incubated at 37 °C for 45 min with Brefeldin A (5 μg/mL), Monensin (2 μM), or Harringtonine (2 μg/mL), then processed for flow cytometry analysis. For the CSF1R recycling assay, whole blood was incubated on ice with PBS containing 100 ng/mL recombinant CSF1 for 30 min. Blood cells were then centrifuged and resuspended using 1.5 mL PBS at least 5 times to wash away any unbound CSF1. To allow CSF1R recycling, blood cells were resuspended in RPMI with or without 2-Deoxy-D-Glucose (100 mM), and incubated at 37 °C for 20–100 min. Red blood cells were then lysed and white blood cells were analyzed by flow cytometry to quantify surface CSF1R expression. To measure glucose uptake, 50 μL whole blood was incubated at 37 °C for 45 min with 50 μM 2-NBDG (Thermofisher cat# N13195) and then directly put on ice. 2-NBDG uptake was assessed by flow cytometry. Cells that were not incubated with 2-NBDG served as negative (full minus one) control.

#### Human cells

Human blood samples were collected using ethylene diamine tetraacetic acid–containing tubes and mononucleated cells were first isolated using Ficoll Hypaque (Eurobio, CMSMSL0101). To measure glucose uptake, 1×10^6^ PBMCs were incubated at 37 °C for 45 min with 100 μM 2-NBDG (Thermofisher cat# N13195) and then directly put on ice. 2-NBDG uptake was assessed by flow cytometry. Cells that were not incubated with 2-NBDG served as negative (full minus one) control.

### In vitro cell treatments

#### Murine in vitro monocyte conversion assay

OP9 cells were grown in propagation media consisting of MEMalpha medium with 10% fetal bovine serum and 500 units of Penicillin Streptomycin. Cells were seeded at 2×10^4^ cells/well in 96-well format. Cell-sorted bone marrow Ly6C^+^ Treml4^+^ or Ly6C^low^ Treml4^+^ monocytes in RPMI medium (containing 10% fetal bovine serum, 50 U/mL Penicillin, 50 μg/mL Streptomycin) were co-cultured at ~5000 cells/well with the OP9 feeder cells for 48 hrs in the presence of 20 ng/ml CSF1 with or without 5 μg/ml AFS98 followed by flow cytometry analysis.

#### Human cells

Monocyte enrichment from human peripheral blood was performed using the autoMACS® Pro Separator (Miltenyi, France) and a negative selection method with an antibody cocktail targeting all mature cells except monocytes (Miltenyi, 130-096-537). Purified monocytes were grown in RPMI 1640 medium with glutamax-I (Life Technologies, 61870044) supplemented with 10% (vol/vol) fetal bovine serum (Life Technologies) and treated with Gilteritinib (1 μM), PLX5622 (5 nM), or a combination of both for 16 h.

### SCENITH analysis

The method was performed as described in ref. ^48^. SCENITH reagents kit (inhibitors, puromycin, and antibodies) were obtained from www.scenith.com/try-it and used according to the provided protocol for ex-vivo analysis of myeloid cells. Blood was collected in heparinized tubes by submandibular bleeding. Whole blood was incubated at 37 °C for 30 min with Puromycin (10 µg/mL) and either Control, 2-Deoxy-D-Glucose (100 mM), Oligomycin (1 µM), 2-Deoxy-D-Glucose + Oligomycin, or Harringtonine (2 µg/mL). Red blood cells were then lyzed using cold BD Pharm Lyse buffer. Cells were first stained with Live/Dead fixable viability dye, washed, and incubated with Fc Block (2.4G2, BioXcell) before surface staining. Cells were fixed and permeabilized using Foxp3 fixation/permeabilization buffer (Miltenyi) and then stained with anti-Puromycin (Alexa Fluor 647). Cells that were not incubated with Puromycin were used as a negative control for anti-Puromycin signal. Cells that received surface staining but no anti-Puromycin staining (full minus one) were used to measure subset-specific autofluorescence in the AF647 channel, and this background signal was subtracted. Glucose dependence, Mitochondrial dependence, and Glycolytic capacity were calculated as previously described^48^.

### Flow cytometry

Tissues were harvested after cervical dislocation and washed in PBS. Splenocytes were prepared by gently crushing the spleen on a 70μm strainer in flow buffer (PBS containing 1% BSA and 2 mM EDTA). Adipose depots were minced with scissors and incubated for 45 min in PBS containing 3 mg/mL collagenase A and 100µg/mL DNAse I at 37 °C. Digested tissues were then homogenized using a 1 mL syringe with a 20G needle and passed through a 70 μm strainer. Bone marrow cells were prepared by flushing femurs and tibias with flow buffer. Blood was drawn from the submandibular vein and collected in heparinized tubes. Leukocyte counts were quantified using a veterinary hematology analyzer (Exigo H400) or directly on the Cytek Aurora cytometer. Red blood cells were lysed from all single cell suspensions using BD Pharm Lyse lysing solution (BdBiosciences cat #555899). Cells were washed with PBS, washed in flow buffer and then stained. For intracellular staining, cells were fixed and permeabilized using Miltenyi Foxp3 staining buffer (cat #130-093-142). All antibodies were used 1/200. A list of all antibodies and reagents used is provided in Supplementary Table 2. Flow cytometry data were acquired using a BD FACS Canto II and a Cytek Aurora. All analyses, including unsupervised analyses, were performed using FlowJo software (Tree Star).

### Seahorse analysis

Oxygen consumption rate (OCR) and extracellular acidification rate (ECAR) of BMDM from Lyz2^ΔGfpt1^ and control littermates were measured with a XF24 Extracellular Flux Analyzer (Seahorse Bioscience; Agilent Technologies, Courtaboeuf, France). BMDM were differentiated from 10^5^ bone marrow cells seeded in the Seahorse 24-well plates and used for analysis after one week of culture with 50 ng/mL recombinant CSF1 (medium refreshed on day 3). Prior to starting the assay, the cells were glucose deprived for an hour in Seahorse XF RPMI assay medium containing 2 mM glutamine. During the assay, glucose (10 mM), oligomycin (1.2 μM), 2-Deoxyglucose (50 mM), and Rotenone and Antimycin-A (2 μM each) were injected consecutively. Data was normalized for genomic DNA measured by quantification of Hoechst 33342 staining (250 nM for 30 min at 37 °C) on a Biotek Synergy HT microplate reader.

### Western blot

Proteins were extracted from BMDM using a Qiagen AllPrep DNA/RNA/protein Mini kit and subsequently resolved by SDS-PAGE and transferred to PVDF membranes by wet transfer during 90 min at 100 V at 4 °C. Membranes were blocked in 5% (m/v) powdered non-fat milk in TBS and incubated with primary antibodies 1:1000 rabbit anti-GFPT1 monoclonal EPR4845 (Ab15069 Abcam) and 1:5000 mouse anti-βactin in blocking solution overnight at 4 °C. After washing, membranes were incubated with HRP-conjugated secondary antibodies for 1 h at room temperature. Proteins were visualized using a Fusion FX (Vilber Lourmat, France).

### Statistical analysis

All data are represented in mean ± SEM. Statistical analysis was performed with GraphPad Prism 10 as indicated in each figure legend. Presence of statistically significant differences between conditions are indicated as follows: ns *p* > 0.05; * *p* < 0.05; ** *p* < 0.01; *** *p* < 0.001; **** *p* < 0.0001.

### Reporting summary

Further information on research design is available in the Nature Portfolio Reporting Summary linked to this article.

## Supplementary information

Supplementary Information

Reporting Summary

Transparent Peer Review file

## Source data

Source Data

Source Data - Supplementary

## Data Availability

No new transcriptomic, proteomic or metabolomic datasets were generated during the study. All data supporting the findings of the study are presented in the main manuscript or in the supplementary information files. Source data are provided with this paper. Requests relative to the data presented herein should be directed to corresponding authors (Stoyan.Ivanov@univ-cotedazur.fr or Alexandre.Gallerand@univ-cotedazur.fr). Access to our data shall be granted permanently to researchers who provide written research aims and are affiliated with a recognized institution. We aim to answer and provide access to requested data within 30 days. Source data are provided with this paper.
