# Supplementary material for: CSF1R regulates monocyte subset differentiation and intracellular metabolism
Source: Nat Commun. 2026 Jul 4;17:8315. doi: 10.1038/s41467-026-75263-7 (PMC13470356; doi:10.1038/s41467-026-75263-7)

Supplementary information for:

CSF1R regulates monocyte subset differentiation and intracellular metabolism.

Author list

Alexandre Gallerand[1#*], Johanna Merlin[2#], Zakariya Caillot[1], Chloé Delaby[3], Elisa Bord[1], Jichang Han[4], Bastien Dolfi[1], Michael D. Chang[5,6], Guilhem R. Thierry[2], Alexia Castiglione[1], Sacha Grenet[1], Eloïse Goës[1], Maxime Franceschini[1], Gisèle Jarretou[1], Fairouz N. Zair[1], Evy Boré[1], Florian Tuffin[1], David Dombrowicz[7], Giulia Chinetti[1], Rodolphe R. Guinamard[1], Maxim N. Artyomov[8], Abdelilah Wakkach[1], Didier F. Pisani[1], Gwendalyn J. Randolph[4], Adeline Bertola[1], Patrick Auberger[3], Arnaud Jacquel[3], David A. Hume[9], Jesse W. Williams[5,6], Marc Bajénoff[2], Jaap G. Neels[1#] and Stoyan Ivanov[1#*]

Affiliations

[1] Université Côte d'Azur, CNRS, LP2M, France.
[2] Centre d'Immunologie Marseille-Luminy, Aix Marseille Univ UM 2, CNRS UMR7280, INSERM U1104, 13009 Marseille, France.
[3] Université Côte d'Azur, C3M, INSERM U1065, Nice, France.
[4] Department of Pathology and Immunology, Washington University School of Medicine, St. Louis, MO, USA.
[5] Center for Immunology, University of Minnesota, Minneapolis, Minnesota, United States.
[6] Department of Integrative Biology and Physiology, University of Minnesota, Minneapolis, Minnesota, United States.
[7] Université de Lille, Inserm, CHU Lille, Institut Pasteur de Lille, U1011-EGID, 59000 Lille, France.
[8] Division of Immunobiology, Department of Pathology and Immunology, Washington University, School of Medicine, St. Louis, MO, United States.
[9] Mater Research Institute-University of Queensland, Translational Research Institute, Brisbane, Queensland, Australia.

# Equally Contributing Authors
* Corresponding authors

Correspondence to:

stoyan.ivanov@univ-cotedazur.fr
alexandre.gallerand@univ-cotedazur.fr

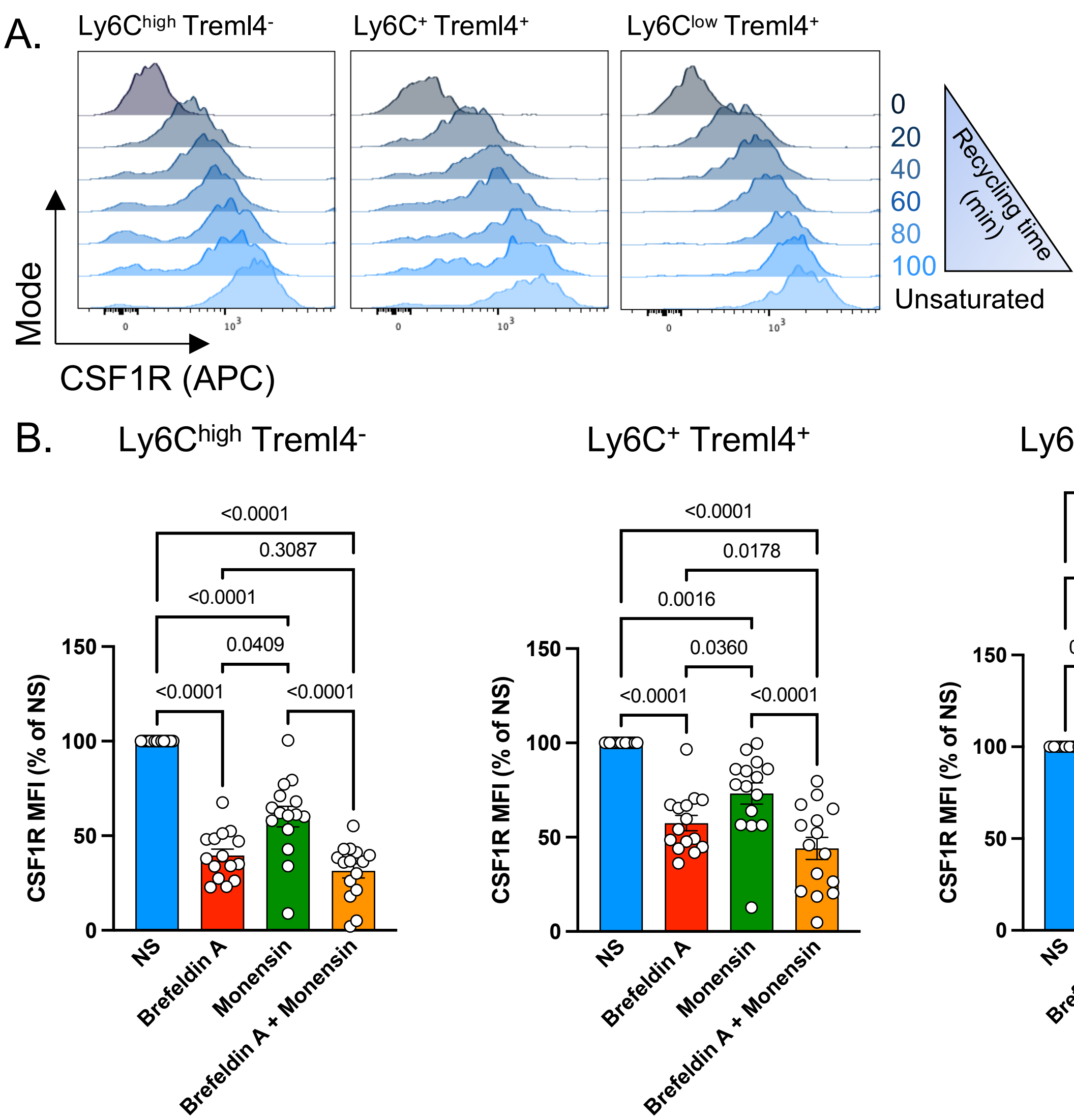

Supplementary Figure 1. Surface CSF1R expression relies on continuous protein tanslation. (**A**) Flow cytometry plots showing CSF1R expression over time in blood monocyte subsets. (**B**) Quantification of CSF1R expression across monocyte subsets from wild-type mice (n=15) after a 45-minute incubation at 37°C with brefeldin A, monensin or both inhibitors. Data pooled from four independent experiments. A repeated-measures one-way ANOVA with Tukey's multiple comparison test was used for statistical analysis in panel B. Data are presented as mean values +/- SEM. See also Figure S1. This figure is linked to Figure 1. Source data are provided as a Source Data file.

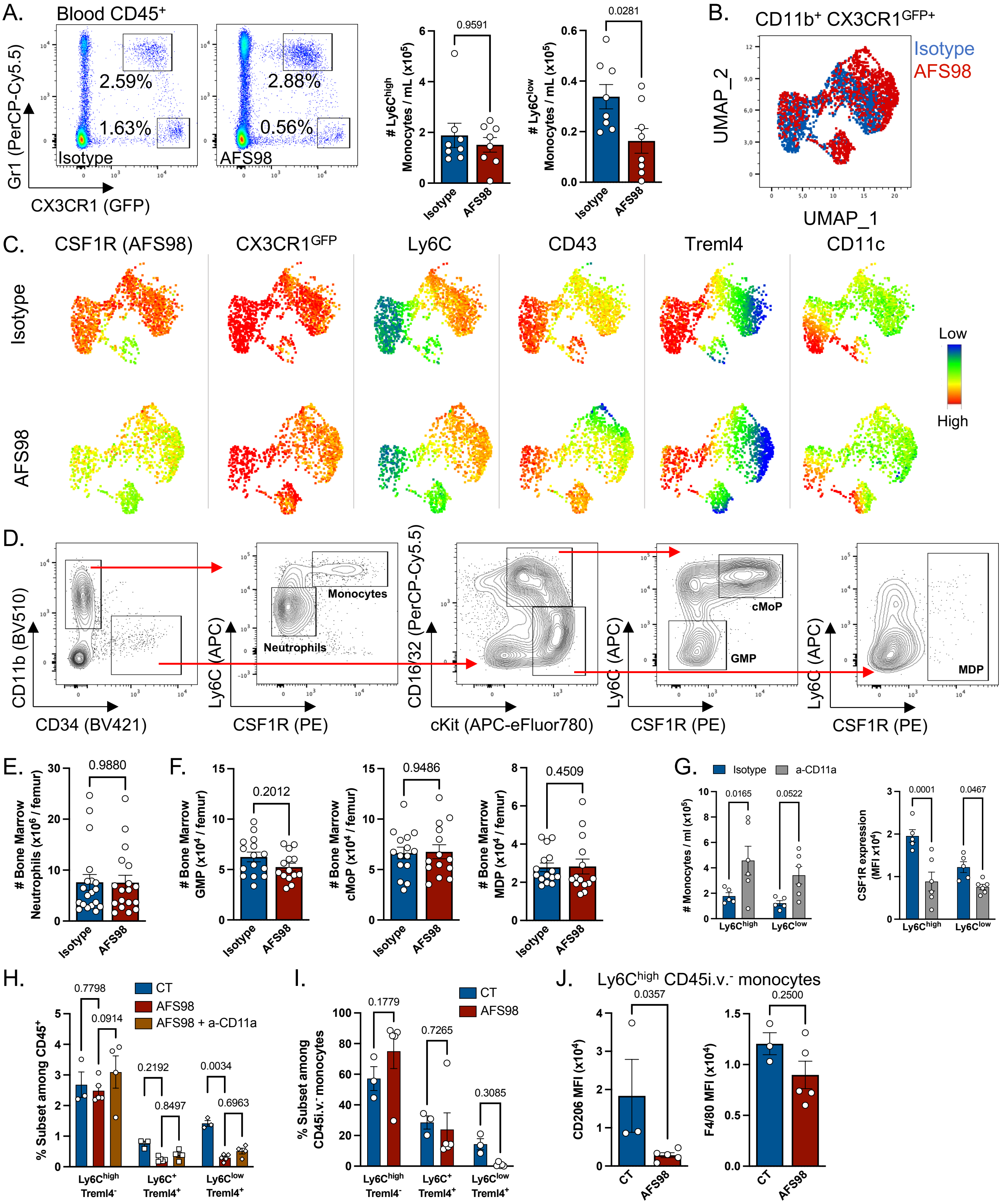

A.
Blood CD45+
Gr1 (PerCP-Cy5.5)
CX3CR1 (GFP)
2.59%
1.63%
Isotype
2.88%
0.56%
AFS98
Ly6Chigh Monocytes / mL (x10^5)
0.9591
Ly6Clow Monocytes / mL (x10^5)
0.0281
Isotype
AFS98
B.
CD11b+ CX3CR1GFP+
Isotype
AFS98
UMAP_2
UMAP_1
C.
CSF1R (AFS98)
CX3CR1GFP
Ly6C
CD43
Treml4
CD11c
Isotype
AFS98
Low
High
D.
CD11b (BV510)
CD34 (BV421)
Ly6C (APC)
CSF1R (PE)
Monocytes
Neutrophils
CD16/32 (PerCP-Cy5.5)
cKit (APC-eFluor780)
cMoP
GMP
MDP
E.
Bone Marrow Neutrophils (x10^6 / femur)
0.9880
F.
Bone Marrow GMP (x10^4 / femur)
0.2012
Bone Marrow cMoP (x10^4 / femur)
0.9486
Bone Marrow MDP (x10^4 / femur)
0.4509
G.
Isotype
a-CD11a
Monocytes / ml (x10^5)
0.0165
0.0522
CSF1R expression (MFI x10^4)
0.0001
0.0467
Ly6Chigh
Ly6Clow
H.
CT
AFS98
AFS98 + a-CD11a
% Subset among CD45+
0.7798
0.0914
0.2192
0.8497
0.0034
0.6963
Ly6Chigh Treml4-
Ly6C+ Treml4+
Ly6Clow Treml4+
I.
CT
AFS98
% Subset among CD45i.v.- monocytes
0.1779
0.7265
0.3085
J.
Ly6Chigh CD45i.v.- monocytes
CD206 MFI (x10^4)
0.0357
F4/80 MFI (x10^4)
0.2500
CT
AFS98

Supplementary Figure 2.

(**A-B**) Representative flow cytometry plots illustrating presence of CSF1R$^+$ Gr1^{high} CX3CR1$^+$ and CSF1R$^+$ Gr1^{low} CX3CR1^{high} blood cells and quantification of these cells (B) following treatment with AFS98 (n=8) or isotype control (n=8). (**B**) Uniform Manifold Approximation and Projection (UMAP) representation of CD11b$^+$ CX3CR1^{GFP+} cells concatenated from isotype-treated and AFS98-treated mice. (**C**) UMAP representation of CSF1R, Ly6C, CD43, Treml4 and CD11c expression by blood CD11b$^+$ CX3CR1^{GFP+} cells from isotype-treated and AFS98-treated mice. (**D**) Gating strategy used to analyze bone marrow cells. (**E**) Quantification of bone marrow neutrophils identified as CD11b$^+$ Ly6G$^+$ cells in mice that received isotype (n=19) or AFS98 (n=18) treatment. Data pooled from four independent experiments. Cells were identified as in Supplementary Figure 2D. (**F**) Quantification of bone marrow myeloid progenitors following isotype (n=15) or AFS98 (n=14) treatment. Data pooled from three independent experiments. Cells were identified as in Supplementary Figure 2D. (**G**) Quantification of blood Ly6C^{high} and Ly6C^{low} monocytes and their expression of CSF1R from mice injected intravenously with isotype control (n=5) or anti-CD11a (n=6) 5 minutes before blood sampling. (**H**) Proportions of monocyte subsets in blood from mice administered PBS (CT, n=3) or AFS98 and intravenously injected with PBS (n=5) or anti-CD11a (n=4) 5 minutes before culling. (**I-J**) Quantification of monocyte subsets (**I**) and their expression of maturation markers (J) among CD45i.v$^-$ monocytes in subcutaneous adipose tissue from mice administered PBS (CT, n=3) or AFS98 (n=5). Cells were gated as in Figure 2G. Two-way ANOVA with uncorrected Fisher's LSD (panel G), Dunnett's multiple comparison (panel H) or Tukey's multiple comparison (panel **I**) tests were used for statistical analysis in panels with more than two groups. Unpaired Mann-Whitney tests were used for statistical analysis in panels A, E, F and J. Data are presented as mean values +/- SEM. This figure is linked to Figure 2. Source data are provided as a Source Data file.

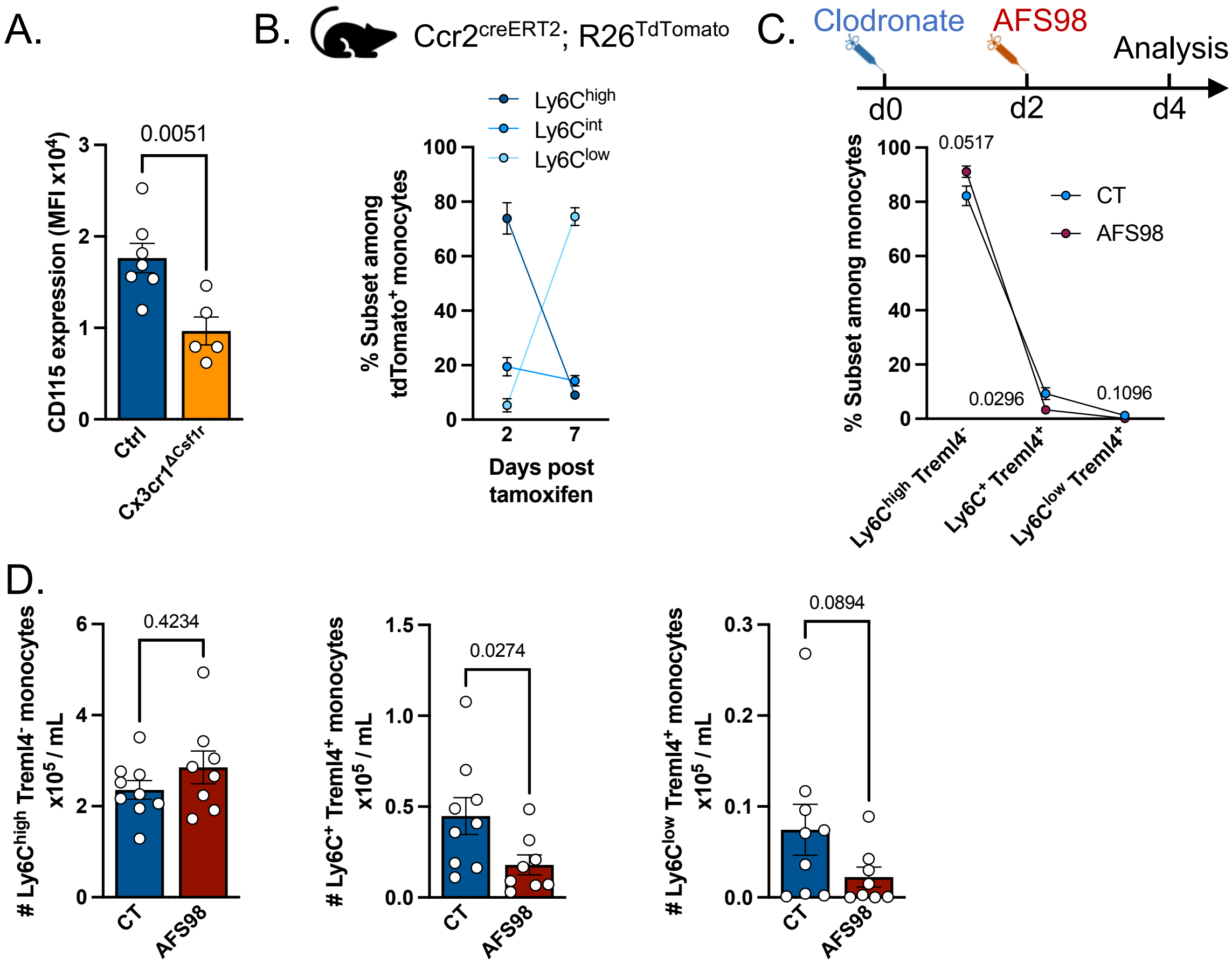

Supplementary Figure 3. CSF1R blockade prevents generation of Treml4^{+} cells from Ly6C^{high} monocytes.

(**A**) CSF1R expression in blood Ly6C^{high} monocytes from Cx3cr1$^{\Delta Csf1r}$ mice (n=5) and littermate controls (n=7). (**B**) Labeling pattern of blood monocytes from Ccr2^{CreERT2}; R26^{tdTomato} mice 2 and 7 days following a single oral administration of tamoxifen. (**C**) Experimental strategy used to deplete blood monocytes via intravenous clodronate injection and test the role of CSF1R blockade on newly-generated monocytes, and proportions of monocyte subsets in mice treated with clodronate + PBS (CT, n=9) or clodronate + AFS98 (n=8). Data pooled from two independent experiments. (**D**) Quantification of blood monocyte subsets in mice treated with clodronate + PBS (CT, n=9) or clodronate + AFS98 (n=8). Data pooled from two independent experiments. A two-way ANOVA with Tukey's multiple comparison test was used for statistical analysis in panel C. Unpaired Mann-Whitney tests were used for statistical analysis in panels A and D. Data are presented as mean values +/- SEM. This figure is related to Figure 3. Source data are provided as a Source Data file.

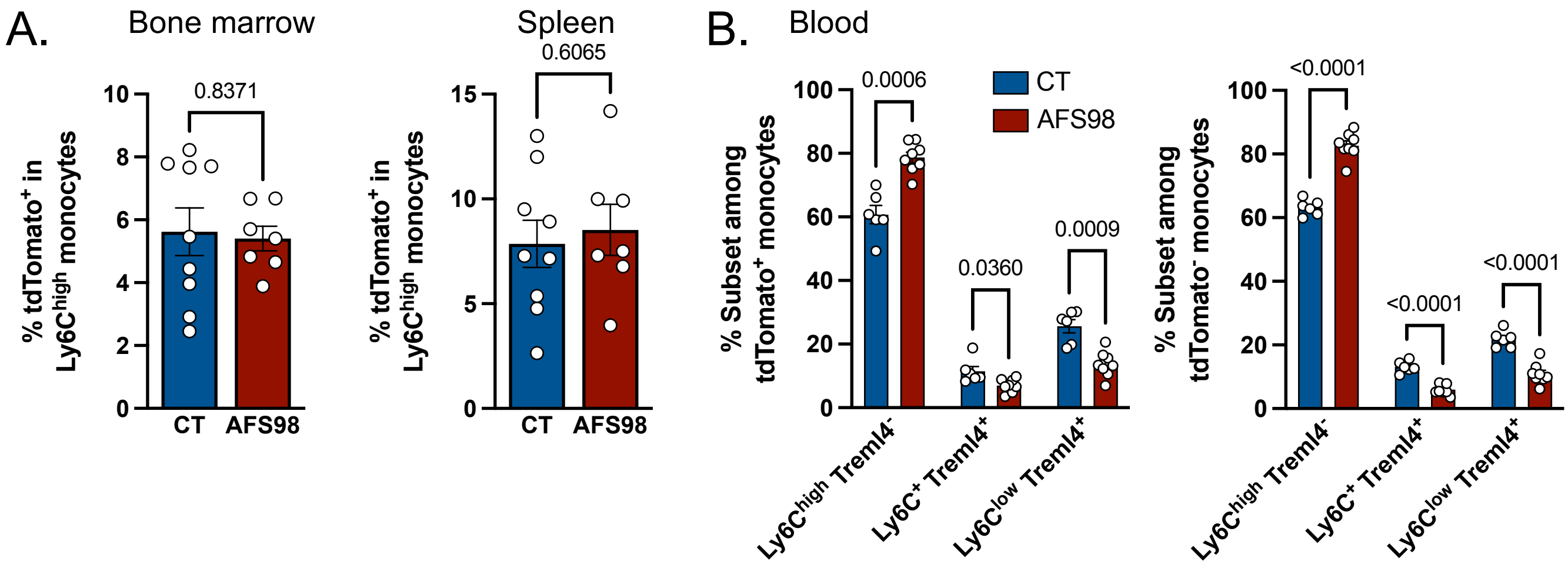

Supplementary Figure 4. Impact of CSF1R blockade on MDP-derived monocytes.
(**A**) Proportions of tdTomato^{+} cells among bone marrow (left) and spleen (right) Ly6C^{high} monocytes from Clec9a^{iCre}; R26^{tdTomato} mice treated with PBS (CT, n=9) or AFS98 (n=7). Data pooled from two independent experiments. (**B**) Proportions of monocyte subsets among tdTomato^{+} (left) and tdTomato^{-} (right) blood monocytes from Clec9a^{iCre}; R26^{tdTomato} mice treated with PBS (CT, n=6) or AFS98 (n=8). Data pooled from two independent experiments. A repeated-measures one-way ANOVA with Tukey's multiple comparison test was used for statistical analysis in panel F. Two-way ANOVA with Tukey's multiple comparison tests were used for statistical analysis in panel B. Unpaired Mann-Whitney tests were used for statistical analysis in panel A. Data are presented as mean values +/- SEM. This figure is related to Figure 5. Source data are provided as a Source Data file.

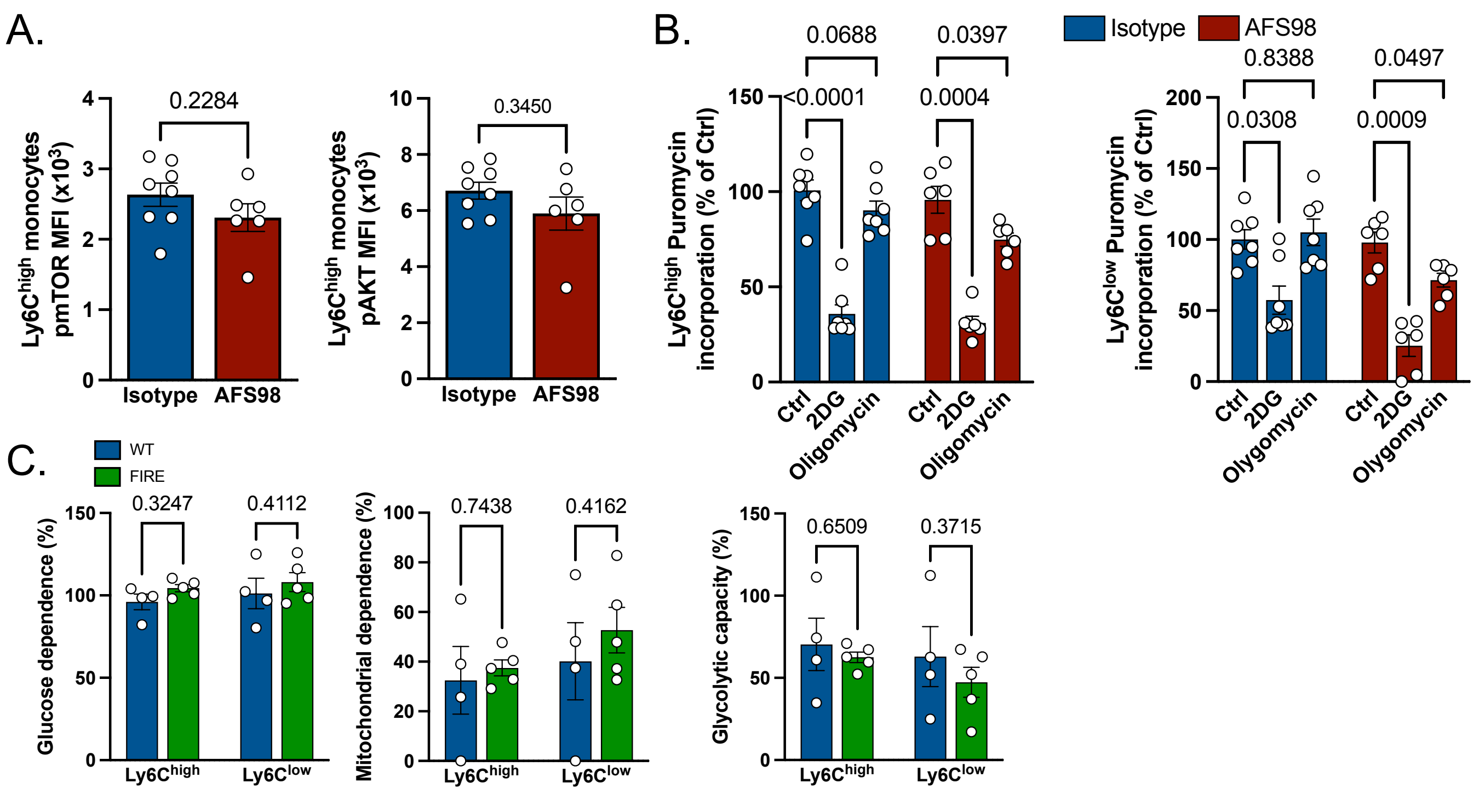

Supplementary Figure 5. Impact of CSF1R blockade on monocyte metabolism.
(**A**) Quantification of mTOR and AKT phosphorylation in blood Ly6C^{high} monocytes following CSF1R blockade (n=6) or isotype control (n=8) administration. (**B**) Puromycin incorporation in Ly6C^{high} (left) and Ly6C^{low} (right) blood monocytes in isotype control (n=7) and AFS98 (n=6) treated mice upon incubation with vehicle (Ctrl), 2-DG or oligomycin. (**C**) Metabolic profile of blood monocytes from Csf1r$^{\Delta FIRE/\Delta FIRE}$ (FIRE, n=5) mice or their littermate Csf1r$^{\Delta FIRE/+}$ controls (WT, n=4) measured using SCENITH. Two-way ANOVA with uncorrected Fisher's LSD (panel C) or Tukey's multiple comparison tests (panel B) were used for statistical analysis in panels with more than two groups. Unpaired Mann-Whitney tests were used for statistical analysis in panel A. Data are presented as mean values +/- SEM. This figure is linked to Figure 6. Source data are provided as a Source Data file.

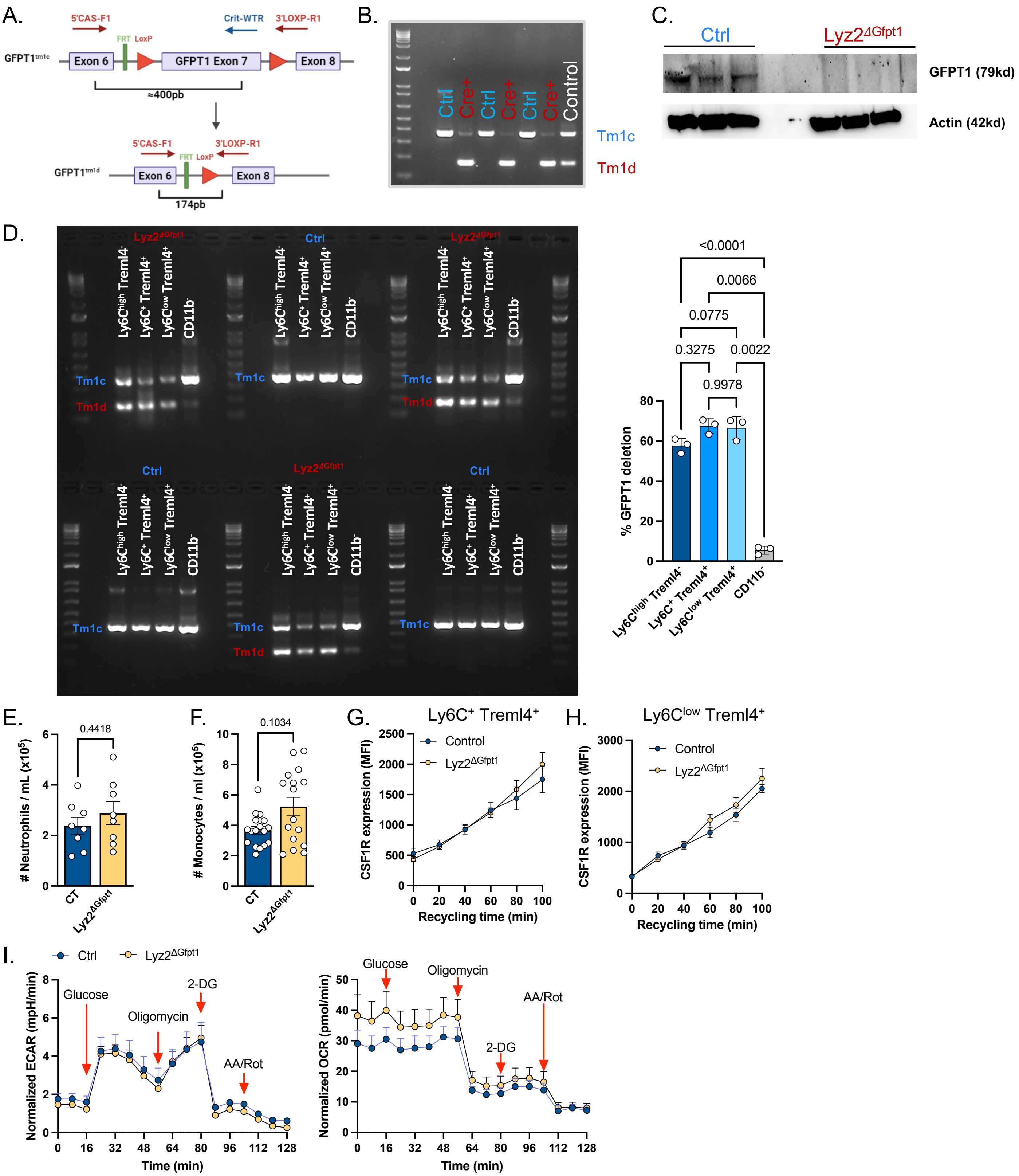

A.
5'CAS-F1
Crit-WTR
3'LOXP-R1
FRT
LoxP
GFPT1tm1c
Exon 6
GFPT1 Exon 7
Exon 8
≈400pb
GFPT1tm1d
174pb
B.
Ctrl
Cre+
Control
Tm1c
Tm1d
C.
Ctrl
Lyz2ΔGfpt1
GFPT1 (79kd)
Actin (42kd)
D.
Lyz2ΔGfpt1
Ctrl
Ly6Chigh Treml4-
Ly6C+ Treml4+
Ly6Clow Treml4+
CD11b-
Tm1c
Tm1d
% GFPT1 deletion
<0.0001
0.0066
0.0775
0.3275
0.0022
0.9978
E.
Neutrophils / mL (x10^5)
0.4418
CT
Lyz2ΔGfpt1
F.
Monocytes / ml (x10^5)
0.1034
G.
Ly6C+ Treml4+
Control
Lyz2ΔGfpt1
CSF1R expression (MFI)
Recycling time (min)
H.
Ly6Clow Treml4+
I.
Ctrl
Lyz2ΔGfpt1
Normalized ECAR (mpH/min)
Normalized OCR (pmol/min)
Glucose
Oligomycin
2-DG
AA/Rot
Time (min)

Supplementary Figure 6. Validation of Lyz2$^{\Delta Gfpt1}$ mice.

(**A**) Construction of myeloid cell-specific GFPT1-deficient mice (Lyz2$^{\Delta Gfpt1}$). Mice carrying GFPT1 Tm1c allele with exon 7 flanked by LoxP sites (GFPT1$^{fl/fl}$) are crossed with Lyz2^{Cre} mice, resulting in the GFPT1 Tm1d allele in which exon 7 has been excised. **(B)** PCR data on genomic DNA from bone marrow derived macrophages (BMDM) from control (GFPT1$^{fl/fl}$) mice or Lyz2$^{\Delta Gfpt1}$ mice, showing the presence of Tm1c and Tm1d alleles. As a control (Ctrl), PCR data was shown for an ear biopsy from a Lyz2$^{\Delta Gfpt1}$ mouse. **(C)** Western blot analysis of GFPT1 expression. Protein extracts were obtained from BMDMs from control and Lyz2$^{\Delta Gfpt1}$ mice. Beta Actin was used as a loading control. (**D**) PCR data on genomic DNA from cell-sorted splenic monocyte subsets from control (GFPT1$^{fl/fl}$) mice (n=3) or Lyz2$^{\Delta Gfpt1}$ mice (n=3), showing the presence of Tm1c and Tm1d alleles. Cell-sorted CD11b^{-} splenocytes were used as a negative control. (**E**) Neutrophil numbers in blood from Lyz2$^{\Delta Gfpt1}$ (n=8) and littermate control (n=8) mice. Data pooled from two independent experiments. (**F**) Monocyte numbers in blood from Lyz2$^{\Delta Gfpt1}$ (n=16) and littermate control (n=17) mice. Data pooled from four independent experiments. (**G-H**) Time-course analysis of surface CSF1R expression on blood Ly6C^{+} Treml4^{+} (G) and Ly6C^{low} Treml4^{+} (H) monocytes from control (n=11) and Lyz2$^{\Delta Gfpt1}$ (n=10) mice following incubation at 37°C to allow receptor recycling. Data pooled from three independent experiments. (**I**) Seahorse analysis of BMDM from control (n=4) and Lyz2$^{\Delta Gfpt1}$ (n=4) mice using the glucose stress test protocol. A repeated measures one-way ANOVA with Tukey's multiple comparison test was used for statistical analysis in panel D. Unpaired Mann-Whitney tests were used for statistical analysis in panels E and F. Data are presented as mean values +/- SEM. This figure is linked to Figure 6. Source data are provided as a Source Data file.

Supplementary Table 1. Genotyping primers

R26$^{LSL\text{-}tdTomato}$	5'AAGGGAGCTGCAGTGGAGTA3'	Wild-type forward primer
	5'CCGAAAATCTGTGGGAAGTC3'	Wild-type reverse primer
	5'CTGTTCCTGTACGGCATGG3'	Mutant forward primer
	5'GGCATTAAAGCAGCGTATCC3'	Mutant reverse primer
Cx3cr1^{GFP}	5'GTCTTCACGTTCGGTCTGGT3'	Wild-type forward primer
	5'CCCAGACACTCGTTGTCCTT3'	Common reverse primer
	5'CTCCCCCTGAACCTGAAAC3'	Mutant forward primer
Lyz2^{cre}	5'GTCACTCACTGCTCCCCTGT3'	Wild-type primer
	5'AAGGAGGGACTTGGAGGATG3'	Common primer
	5' ACCGGTAATGCAGGCAAAT3'	Mutant primer
Csf1r$^{\Delta FIRE}$	5′GCTGCCCTGTCACTGTGTA3'	Forward primer
	5'TCGTTTCCCATCCCAGGA3'	Reverse primer
Clec9a^{iCre}	5'CAGGTTTTGGTGCACAGTCA3'	Mutant reverse primer
	5'TGTTTCAGTATAAGCTTCAAAAGTTCC3'	Common primer
	5'GAATGTCCCACTGAAGAGAGG3'	Wild-type reverse primer
Cx3cr1^{CreERT2}	5'AGCTCACGACTGCCTTCTTC3'	Wild-type forward primer
	5'ACGCCCAGACTAATGGTGAC3'	Common primer
	5'GTTAATGACCTGCAGCCAAG3'	Mutant forward primer
Ccr2^{CreERT2}	5′CTCTACTTCATCGCATTCCTTGC3′	Forward primer
	5′GGTTGATGAAGGTTTTGCTGC3′	Reverse primer
Ms4a3^{FlpO}	5'AGAGAAATCATCAGGGCAGAAAT3'	Primer 1
	5'GAAAGGGGAACAAGCGAAGAT3'	Primer 2
	5'TTGGCGAGAGGGGAAAGAC3'	Primer 3
R26$^{FSF\text{-}tdTomato}$	5'ACGGGCAGTAGGGCTGAG3'	Primer 1
	5'AGCCTGCCCAGAAGACTCC3'	Primer 2
	5'GGTGTTGGGTCGTTTGTTCA3'	Primer 3
	5'TCTAGCTTGGGCTGCAGGT3'	Primer 4
Cx3cr1^{Cre}	5'ACGTGGACCTGCTTACTGCATG3'	Forward primer
	5'CGGCAAACGGACAGAAGCATT3'	Reverse primer
Csf1^{flox}	5'ACAGGCAGGCCTCTGATCTA3'	Forward primer
	5'CCCAGCCAAGAATTCTCCTT3'	Reverse primer
	5'GGGCACTCTCCATCTTACCA3'	Null primer
Cdh5^{CreERT2}	5'CCAGGCTAAGTGCCTTCTCTACA3'	Forward primer
	5'AATGCTTCTGTCCGTTTGCCGGT3'	Reverse primer
Gfpt1^{flox}	5'AAGGCGCATAACGATACCAC3'	5'CAS-F1
	5'GCTCCCGTTCCAATACTCAA3'	Crit-WTR
	5'ACTGATGGCGAGCTCAGACC3'	3'LOXP-R1

Supplementary Table 2. Key Resources Table

Reagent or resource	Source	Identifier
**Antibodies**		
α-mouse CSF1R PE (clone AFS98)	Biolegend	Cat# 135506
α-mouse CSF1R BV711 (clone AFS98)	Biolegend	Cat# 135515
α-mouse CSF1R APC (clone AFS98)	Biolegend	Cat# 135510
α-mouse/human CD11b Brilliant Violet 510 (clone M1/70)	Biolegend	Cat# 101263
α-mouse/human CD11b PE-Cy5 (clone M1/70)	Biolegend	Cat# 101210
α-mouse/human CD11b BV750 (clone M1/70)	Biolegend	Cat# 101267
α-mouse/human CD11b APC-Cy7 (clone M1/70)	Biolegend	Cat# 101226
α-mouse/human CD11b BUV737 (clone M1/70)	BD Biosciences	Cat# 612801
α-mouse Treml4 PE (clone 16E5)	Biolegend	Cat# 143304
α-mouse Treml4 BV421 (clone 16E5)	BD Biosciences	Cat# 569880
α-mouse CD43 AF700 (clone S11)	Biolegend	Cat# 143214
α-mouse Gr1 PerCP-Cy5.5 (clone RB6-8C5)	BD Biosciences	Cat# 552093
α-mouse Gr1 FITC (clone RB6-8C5)	Biolegend	Cat# 108406
α-mouse CD16/32 PerCP-Cy5.5 (clone 93)	Biolegend	Cat# 156624
α-mouse CD34 BV421 (clone SA376A4)	Biolegend	Cat# 152208
α-mouse Ly6C BV421 (clone HK1.4)	Biolegend	Cat# 128032
α-mouse Ly6C BV711 (clone HK1.4)	Biolegend	Cat# 128037
α-mouse Ly6C BV605 (clone HK1.4)	Biolegend	Cat# 128036
α-mouse Ly6C FITC (clone HK1.4)	Biolegend	Cat# 128006
α-mouse Ly6C AF700 (clone HK1.4)	Biolegend	Cat# 128024
α-mouse Ly6G BV785 (clone 1A8)	Biolegend	Cat# 127645
α-mouse F4/80 PE-Cy7 (clone BM8)	Biolegend	Cat# 123114
α-mouse F4/80 AF488 (clone BM8)	Biolegend	Cat# 123120
α-mouse F4/80 SparkNir685 (clone BM8)	Biolegend	Cat# 123168
α-mouse CD45 APC-Cy7 (clone 30-F11)	Biolegend	Cat# 103116
α-mouse CD45 FITC (clone 30-F11)	Biolegend	Cat# 103108
α-mouse CD45 BV570 (clone 30-F11)	Biolegend	Cat# 103136
α-mouse CD64 Brillant Violet 421 (clone X54-5/7.1)	Biolegend	Cat# 139309
α-mouse CD64 PE-Cy7 (clone X54-5/7.1)	Biolegend	Cat# 139314
α-mouse CD11c PE-Cy5 (clone N418)	Biolegend	Cat# 117316
α-mouse MHC-II (IA/IE) PB (clone M5/114.15.2)	Biolegend	Cat# 107620
α-mouse MHC-II (IA/IE) VioBlue (clone M5/114.15.2)	Miltenyi Biotec	Cat# 130-123-278
α-mouse MHC-II (IA/IE) PE-Fire810 (clone M5/114.15.2)	Biolegend	Cat# 107667
α-mouse Sca1 PB (clone D7)	Biolegend	Cat# 108120
α-mouse Sca1 PE-Cy7 (clone D7)	Biolegend	Cat# 108114
α-mouse c-Kit APC-Cy7 (clone ACK2)	eBioscience	Cat# 47-1172-82

α-mouse CD48 AF488 (clone HM48-1)	Biolegend	Cat# 103414
α-mouse CCR2 PE (clone REA538)	Miltenyi Biotec	Cat# 130-117-548
α-mouse CCR2 APC-Fire750 (clone SA203G11)	Biolegend	Cat# 150630
α-mouse CD206 APC (clone C068C2)	Biolegend	Cat# 141708
α-mouse CD206 AF700 (clone C068C2)	Biolegend	Cat# 141734
α-mouse CD135 PE-Cy5 (clone A2F10)	Biolegend	Cat# 135312
α-mouse CD177 AF647 (clone Y127)	BD Biosciences	Cat# 566599
α-mouse CD319 BV650 (clone 4G2)	BD Biosciences	Cat# 747995
α-mouse CD62L BUV805 (clone MEL-14)	BD Biosciences	Cat# 569201
α-mouse Phospho-mTor (Ser2448) PE-Cy7 (clone MRRBY)	Fischer Scientific	Cat# 15508676
α -mouse Phospho-AKT1 (Ser473) PE (clone SDRNR)	Fischer Scientific	Cat# 15597216
α-Puromycin AF647 (clone 12D10)	Merck	Cat# MABE343-AF647
InVivoMAb anti-mouse LFA-1α (CD11a) (clone M17/4)	BioXcell	Cat# BE0006
InVivoMAb anti-mouse CSF1R (Clone AFS98)	BioXcell	Cat# BE0213
InVivoMAb rat IgG2a isotype control, anti-trinitrophenol	BioXcell	Cat# BE0089
α-mouse GFPT1 (EPR4854)	Abcam	Cat# AB125069
α-mouse Fc Block (clone 2.4G2)	BioXcell	Cat# BE0307
α-human CD45 APC-Fire810 (clone HI30)	Biolegend	Cat# 304076
α-human CD14 FITC (clone M5E2)	Biolegend	Cat# 301804
α-human CD172a PE (clone 15-414)	Biolegend	Cat# 372104
α-human CD16 BV421 (clone 3G8)	Biolegend	Cat# 302038
**Reagents**		
DAPI	Sigma	Cat# D9542
PFA 4%	VWR International	Cat# 9713.1000
Bovine serum Albumin (BSA)	Sigma	Cat# A7030
Collagenase A	Sigma	Cat# 11088793001
DNAse I	Sigma	Cat# 10104159001
RBC lysing buffer	BD Biosciences	Cat# 555899
DreamTaq Green PCR Master Mix (2X)	Thermo Scientific	Cat# K1081
Brilliant Stain Buffer Plus	BD Biosciences	Cat# 566385
EDTA	Sigma	Cat # 324504-500ML
Live/Dead fixable viability dye	Thermofisher	Cat # L34955
Zombie NIR™ Fixable Viability Kit	Biolegend	Cat # 423106
2-NBDG	Thermofisher	Cat# N13195
Harringtonine	MedchemExpress	Cat# HY-N0862
Brefeldin A	Biolegend	Cat# 420601
Monensin	Biolegend	Cat# 420701
Puromycin	Merck	Cat# P7255
PLX5622	MedchemExpress	Cat# HY-114153
Gilteritinib	MedchemExpress	Cat# HY-12432

Antimycin A	Sigma	Cat# A8674-25MG
Rotenone	Sigma	Cat# 557368-1GM
2-DG	Sigma	Cat# D6134-5G
Oligomycin A	Sigma	Cat# 75351-5MG
D-(+)-glucose powder	Sigma	Cat# G7021-100G
Corn oil	Sigma	Cat# C8267
Tamoxifen	Sigma	Cat# T5648
Tamoxifen diet	Safe	Cat# E8220-V0366
Recombinant murine CSF1	Miltenyi Biotec	Cat# 130-101-706
MEMalpha medium	Thermofisher	Cat# 12561056
RPMI medium	Life Technologies	Cat# 21875091
Glutamax-I	Life Technologies	Cat# 61870044
Fetal bovine serum	Fisher Scientific	Cat# 12350273
Penicillin Streptomycin	Life Technologies	Cat# 15070063
Foxp3 fixation/permeabilization kit	Miltenyi Biotec	Cat#130-093-142
Ficoll Hypaque	Eurobio	Cat# CMSMSL0101
Clodronate liposomes	Liposoma	Cat# CP-005-005
Pan Monocyte Isolation Kit, human	Miltenyi Biotec	Cat# 130-096-537
**Accessories**		
StepOne	Applied Biosystem	N/A
Thermo Cycler SimpliAmp	Applied Biosystem	N/A
Nanodrop	OZYME	N/A
Aurora spectral cytometer	Cytek	N/A
BD FACS Canto II	BD Biosciences	N/A
BD FACS Aria IIu	BD Biosciences	N/A
Seahorse analyzer	Agilent Technologies	XFe24
autoMACS® Pro Separator	Miltenyi Biotec	N/A
**Softwares**		
Prism10	GraphPad	N/A
FlowJo	Tree Star	N/A
StepOne Software v.2.2.2	Applied Biosystem	N/A

Uncropped gels

Supplementary Figure 6B. Raw

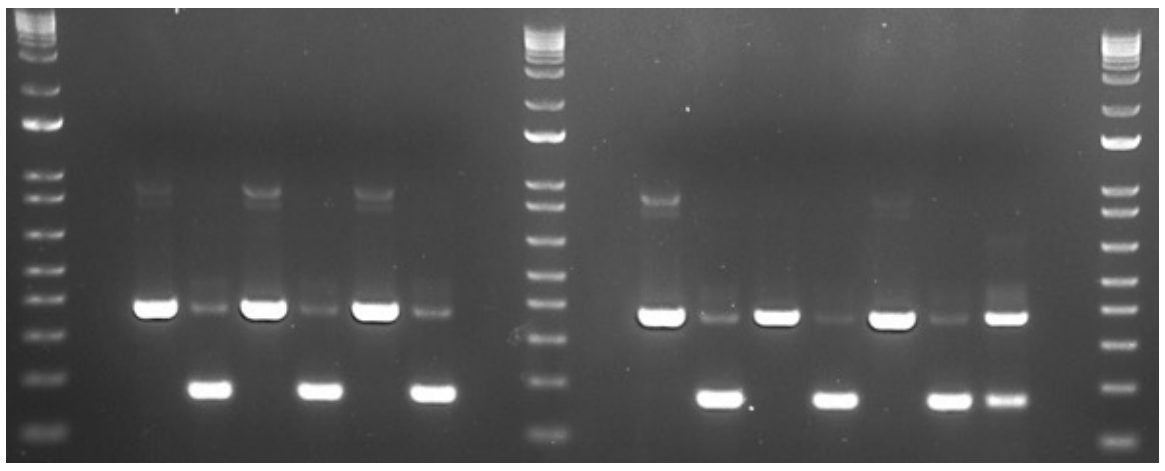

Supplementary Figure 6B. Annotated

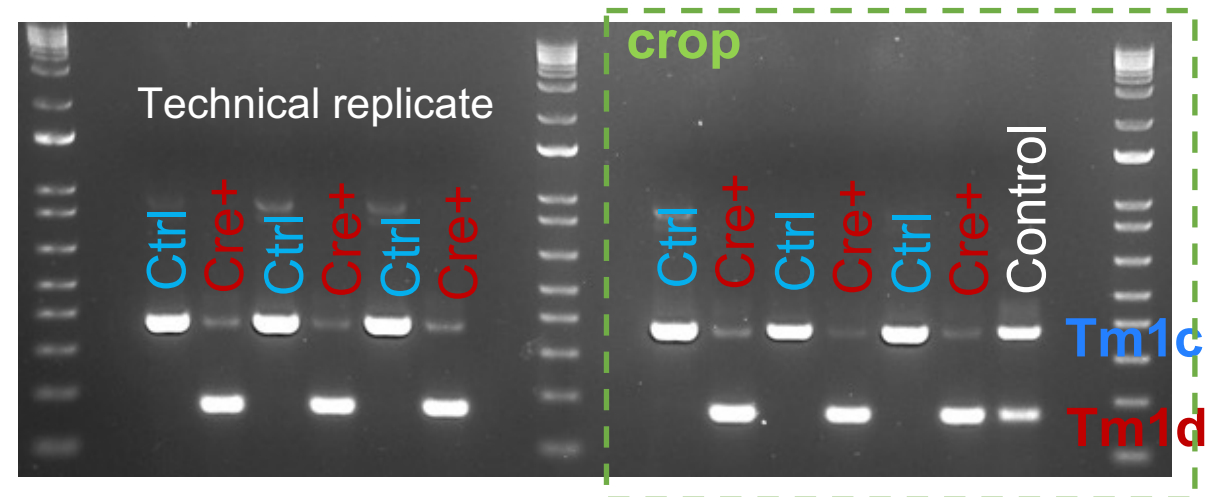

Supplementary Figure 6C. Raw images

Bright field

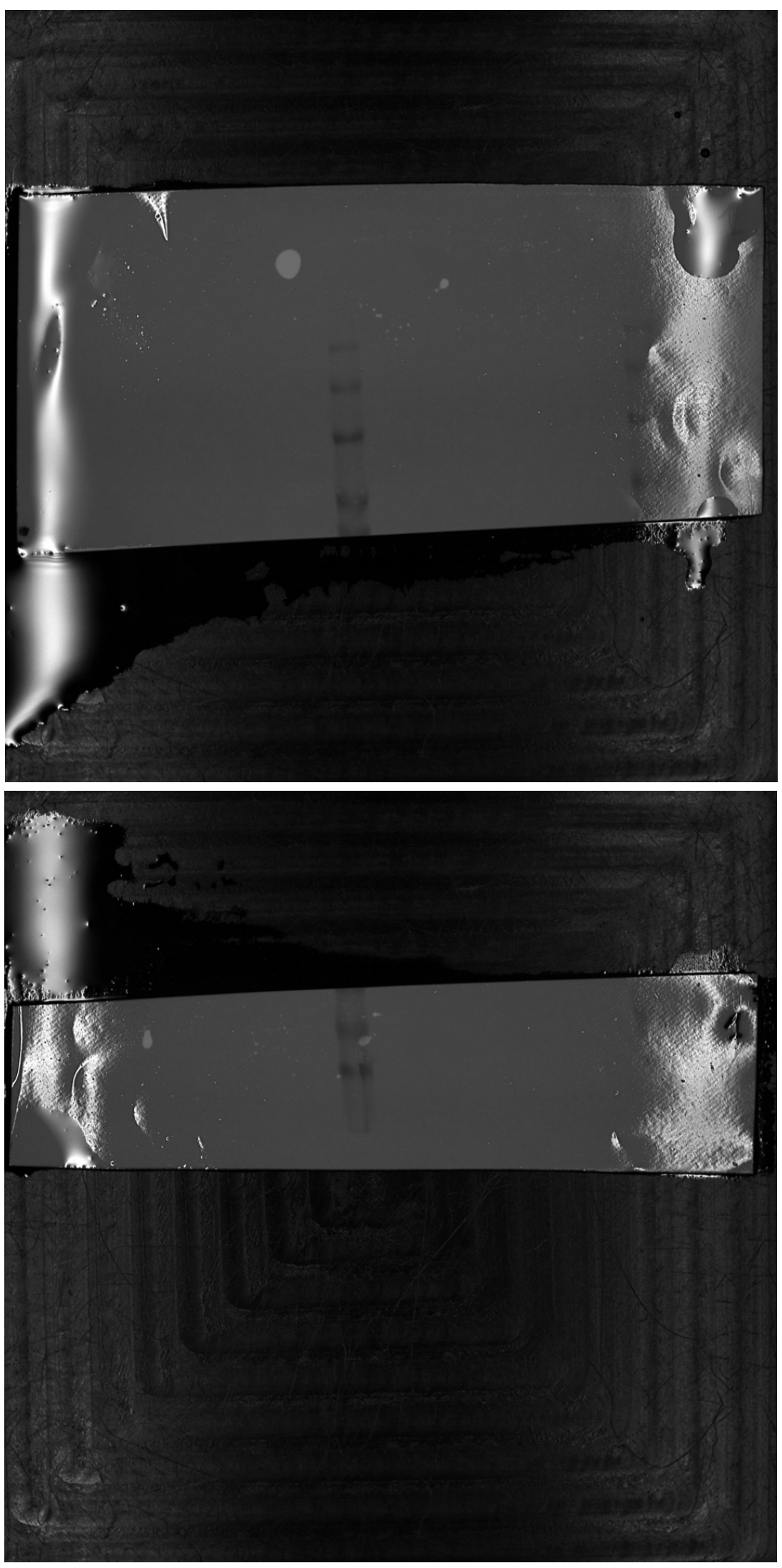

Chemiluminescence

anti-GFPT1

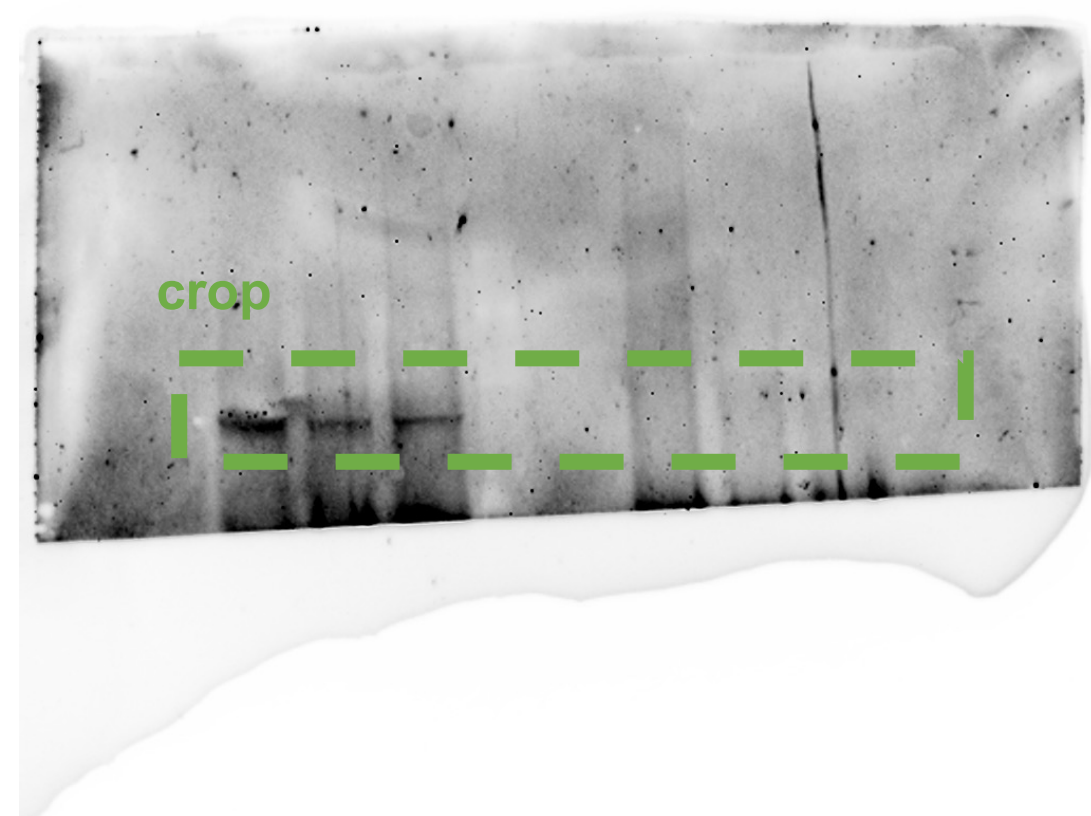

anti-β–Actin

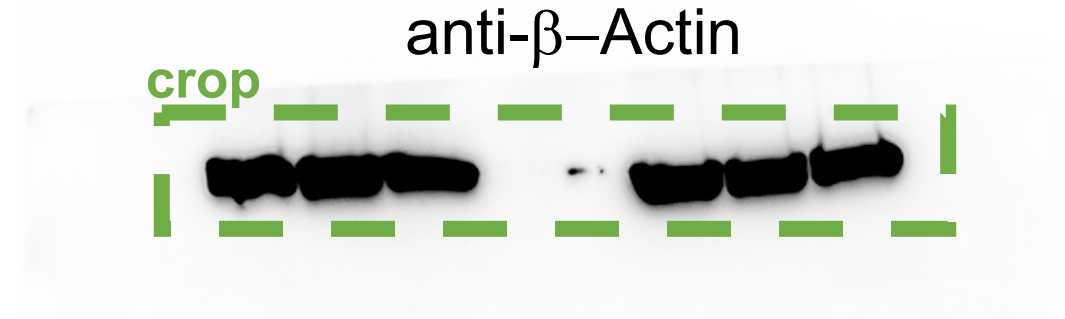

Supplementary Figure 6D. Raw

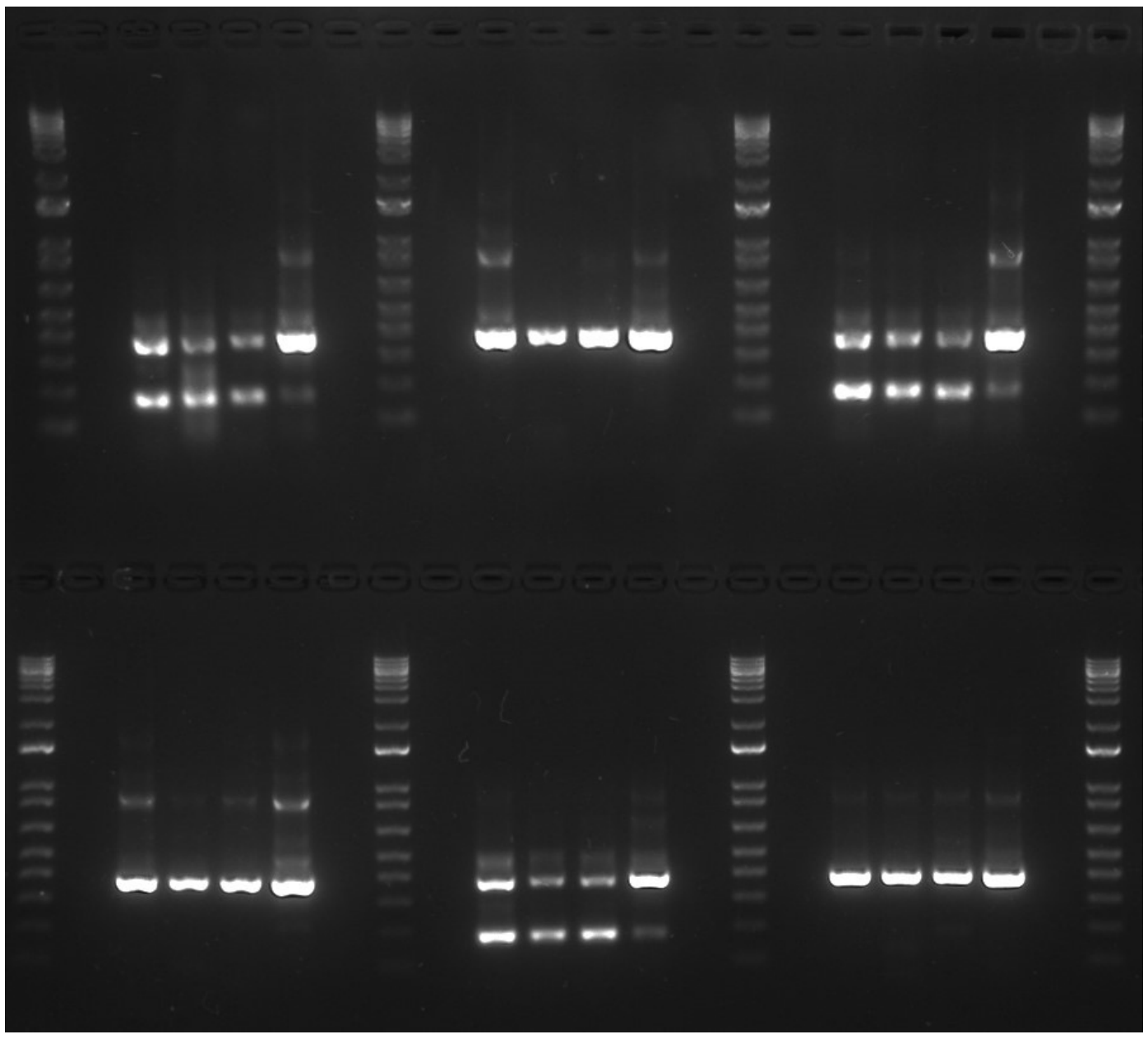

Supplementary Figure 6D. Annotated

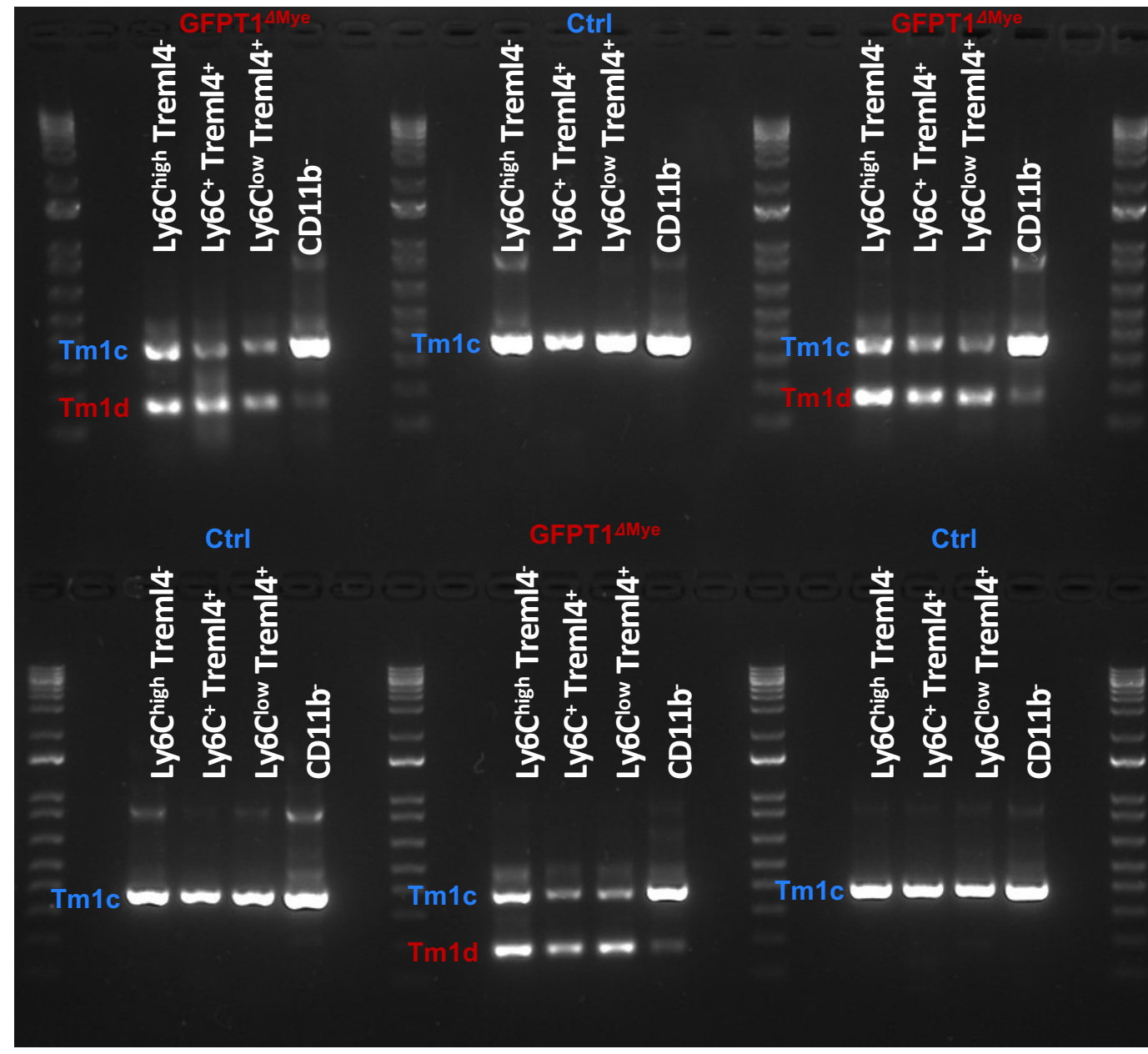

Supplement: Supplementary file 1 [file 41467_2026_75263_MOESM1_ESM.pdf]
