# Supplementary material for: CSF1R regulates monocyte subset differentiation and intracellular metabolism
Source: Nat Commun. 2026 Jul 4;17:8315. doi: 10.1038/s41467-026-75263-7 (PMC13470356; doi:10.1038/s41467-026-75263-7)
Supplement: Supplementary file 2 [file 41467_2026_75263_MOESM2_ESM.pdf]

nature portfolio

Corresponding author(s): Stoyan Ivanov, Alexandre Gallerand

Last updated by author(s): Jun 8, 2026

Reporting Summary

Nature Portfolio wishes to improve the reproducibility of the work that we publish. This form provides structure for consistency and transparency in reporting. For further information on Nature Portfolio policies, see our Editorial Policies and the Editorial Policy Checklist.

Statistics

For all statistical analyses, confirm that the following items are present in the figure legend, table legend, main text, or Methods section.

n/a	Confirmed	
☐	☒	The exact sample size (*n*) for each experimental group/condition, given as a discrete number and unit of measurement
☐	☒	A statement on whether measurements were taken from distinct samples or whether the same sample was measured repeatedly
☐	☒	The statistical test(s) used AND whether they are one- or two-sided<br>*Only common tests should be described solely by name; describe more complex techniques in the Methods section.*
☒	☐	A description of all covariates tested
☐	☒	A description of any assumptions or corrections, such as tests of normality and adjustment for multiple comparisons
☐	☒	A full description of the statistical parameters including central tendency (e.g. means) or other basic estimates (e.g. regression coefficient) AND variation (e.g. standard deviation) or associated estimates of uncertainty (e.g. confidence intervals)
☐	☒	For null hypothesis testing, the test statistic (e.g. *F*, *t*, *r*) with confidence intervals, effect sizes, degrees of freedom and *P* value noted<br>*Give P values as exact values whenever suitable.*
☒	☐	For Bayesian analysis, information on the choice of priors and Markov chain Monte Carlo settings
☒	☐	For hierarchical and complex designs, identification of the appropriate level for tests and full reporting of outcomes
☒	☐	Estimates of effect sizes (e.g. Cohen's *d*, Pearson's *r*), indicating how they were calculated

Our web collection on statistics for biologists contains articles on many of the points above.

Software and code

Policy information about availability of computer code

Data collection	Flow cytometry data were collected using a Cytek Aurora spectral flow cytometer with SpectroFlo software and a BD Canto II II with Diva software.
Data analysis	Microsoft Excel.<br>Flowjo V10.<br>Graphpad Prism 10.

For manuscripts utilizing custom algorithms or software that are central to the research but not yet described in published literature, software must be made available to editors and reviewers. We strongly encourage code deposition in a community repository (e.g. GitHub). See the Nature Portfolio guidelines for submitting code & software for further information.

Data

Policy information about availability of data

All manuscripts must include a data availability statement. This statement should provide the following information, where applicable:

- Accession codes, unique identifiers, or web links for publicly available datasets
- A description of any restrictions on data availability
- For clinical datasets or third party data, please ensure that the statement adheres to our policy

No new transcriptomic, proteomic or metabolomic datasets were generated during the study. All data supporting the findings of the study are presented in the

main manuscript or in the supplementary information files. Source data are provided with this paper. Requests relative to the data presented herein should be directed to corresponding authors (Stoyan.Ivanov@univ-cotedazur.fr or Alexandre.Gallerand@univ-cotedazur.fr). Access to our data shall be granted permanently to researchers who provide written research aims and are affiliated with a recognized institution. We aim to answer and provide access to requested data within 30 days.

Research involving human participants, their data, or biological material

Policy information about studies with human participants or human data. See also policy information about sex, gender (identity/presentation), and sexual orientation and race, ethnicity and racism.

Reporting on sex and gender	Biological sex and age were not considered in the study design as this information was not collected.
Reporting on race, ethnicity, or other socially relevant groupings	Human blood donors were recruited solely on their will to donate blood for reasearch purposes to the Etablissement Français du Sang, and not on other social factors.
Population characteristics	Blood donors were healthy without any known conditions or current treatments, according to criteria of the Etablissement Français du Sang.
Recruitment	Participants voluntered to donate blood to the Etablissement Français du Sang. All participants provided written informed consent.
Ethics oversight	Etablissement Français du Sang, convention numbers 13-PP-11 and NQNA04.

Note that full information on the approval of the study protocol must also be provided in the manuscript.

Field-specific reporting

Please select the one below that is the best fit for your research. If you are not sure, read the appropriate sections before making your selection.

☒ Life sciences ☐ Behavioural & social sciences ☐ Ecological, evolutionary & environmental sciences

For a reference copy of the document with all sections, see nature.com/documents/nr-reporting-summary-flat.pdf

Life sciences study design

All studies must disclose on these points even when the disclosure is negative.

Sample size	No sample size calculations were performed. To comply with the principles of the 3Rs (Replacement, Reduction and Refinement), we have used minimal sample size sufficient to detect biological differences. Numbers of samples are indicated in the figure legends for each panel.
Data exclusions	We pre-established data exclusion criteria in order to avoid variance increases by outliers that would prevent us from drawing statistically meaningful conclusions, and creating the need for further experimentation on animal subjects. This initiative is in line withe the principle of the 3Rs. We determined that (1) mouse agressive behaviour and/or presence of fight wounds and (2) positivity to Grubb's test would be independent criteria for exclusion. Statistical outliers were identified using Grubb's test on the GraphPad Prism software.
Replication	The number of biologically-independent samples is indicated in figure legends corresponding to each panel.
Randomization	"Control" and "test" mouse groups were age-matched and sex-matched for in vivo experiment. Mice were randomly attributed to each experimental group when possible, depending on their genotype. Furthermore, if possible, animals were co-housed. In ex vivo experiments samples were also matched for age and sex. For in vitro experiments all allocations were random.
Blinding	Experimentators were blinded to group allocation during mouse treatment, tissue collection and processing, and during acquisition of flow cytometry data. Experimentators were not blinded to group allocation during analysis of flow cytometry data.

Reporting for specific materials, systems and methods

We require information from authors about some types of materials, experimental systems and methods used in many studies. Here, indicate whether each material, system or method listed is relevant to your study. If you are not sure if a list item applies to your research, read the appropriate section before selecting a response.

Materials & experimental systems

n/a	Involved in the study
☐	☒ Antibodies
☐	☒ Eukaryotic cell lines
☒	☐ Palaeontology and archaeology
☐	☒ Animals and other organisms
☒	☐ Clinical data
☒	☐ Dual use research of concern
☒	☐ Plants

Methods

n/a	Involved in the study
☒	☐ ChIP-seq
☐	☒ Flow cytometry
☒	☐ MRI-based neuroimaging

Antibodies

Antibodies used

anti-mouse CSF1R PE (clone AFS98) Biolegend Cat# 135506
anti-mouse CSF1R BV711 (clone AFS98) Biolegend Cat# 135515
anti-mouse CSF1R APC (clone AFS98) Biolegend Cat# 135510
anti-mouse/human CD11b Brilliant Violet 510 (clone M1/70) Biolegend Cat# 101263
anti-mouse/human CD11b PE-Cy5 (clone M1/70) Biolegend Cat# 101210
anti-mouse/human CD11b BV750 (clone M1/70) Biolegend Cat# 101267
anti-mouse/human CD11b APC-Cy7 (clone M1/70) Biolegend Cat# 101226
anti-mouse/human CD11b BUV737 (clone M1/70) BD Biosciences Cat# 612801
anti-mouse Treml4 PE (clone 16E5) Biolegend Cat# 143304
anti-mouse Treml4 BV421 (clone 16E5) BD Biosciences Cat# 569880
anti-mouse CD43 AF700 (clone S11) Biolegend Cat# 143214
anti-mouse Gr1 PerCP-Cy5.5 (clone RB6-8C5) BD Biosciences Cat# 552093
anti-mouse Gr1 FITC (clone RB6-8C5) Biolegend Cat# 108406
anti-mouse CD16/32 PerCP-Cy5.5 (clone 93) Biolegend Cat# 156624
anti-mouse CD34 BV421 (clone SA376A4) Biolegend Cat# 152208
anti-mouse Ly6C BV421 (clone HK1.4) Biolegend Cat# 128032
anti-mouse Ly6C BV711 (clone HK1.4) Biolegend Cat# 128037
anti-mouse Ly6C BV605 (clone HK1.4) Biolegend Cat# 128036
anti-mouse Ly6C FITC (clone HK1.4) Biolegend Cat# 128006
anti-mouse Ly6C AF700 (clone HK1.4) Biolegend Cat# 128024
anti-mouse Ly6G BV785 (clone 1A8) Biolegend Cat# 127645
anti-mouse F4/80 PE-Cy7 (clone BM8) Biolegend Cat# 123114
anti-mouse F4/80 AF488 (clone BM8) Biolegend Cat# 123120
anti-mouse F4/80 SparkNir685 (clone BM8) Biolegend Cat# 123168
anti-mouse CD45 APC-Cy7 (clone 30-F11) Biolegend Cat# 103116
anti-mouse CD45 FITC (clone 30-F11) Biolegend Cat# 103108
anti-mouse CD45 BV570 (clone 30-F11) Biolegend Cat# 103136
anti-mouse CD64 Brillant Violet 421 (clone X54-5/7.1) Biolegend Cat# 139309
anti-mouse CD64 PE-Cy7 (clone X54-5/7.1) Biolegend Cat# 139314
anti-mouse CD11c PE-Cy5 (clone N418) Biolegend Cat# 117316
anti-mouse MHC-II (IA/IE) PB (clone M5/114.15.2) Biolegend Cat# 107620
anti-mouse MHC-II (IA/IE) VioBlue (clone M5/114.15.2) Miltenyi Biotec Cat# 130-123-278
anti-mouse MHC-II (IA/IE) PE-Fire810 (clone M5/114.15.2) Biolegend Cat# 107667
anti-mouse Sca1 PB (clone D7) Biolegend Cat# 108120
anti-mouse Sca1 PE-Cy7 (clone D7) Biolegend Cat# 108114
anti-mouse c-Kit APC-Cy7 (clone ACK2) eBioscience Cat# 47-1172-82
anti-mouse CD48 AF488 (clone HM48-1) Biolegend Cat# 103414
anti-mouse CCR2 PE (clone REA538) Miltenyi Biotec Cat# 130-117-548
anti-mouse CCR2 APC-Fire750 (clone SA203G11) Biolegend Cat# 150630
anti-mouse CD206 APC (clone C068C2) Biolegend Cat# 141708
anti-mouse CD206 AF700 (clone C068C2) Biolegend Cat# 141734
anti-mouse CD135 PE-Cy5 (clone A2F10) Biolegend Cat# 135312
anti-mouse CD177 AF647 (clone Y127) BD Biosciences Cat# 566599
anti-mouse CD319 BV650 (clone 4G2) BD Biosciences Cat# 747995
anti-mouse CD62L BUV805 (clone MEL-14) BD Biosciences Cat# 569201
anti-mouse Phospho-mTor (Ser2448) PE-Cy7 (clone MRRBY) Fischer Scientific Cat# 15508676
anti-mouse Phospho-AKT1 (Ser473) PE (clone SDRNR) Fischer Scientific Cat# 15597216
anti-Puromycin AF647 (clone 12D10) Merck Cat# MABE343-AF647
InVivoMAb anti-mouse LFA-1α (CD11a) (clone M17/4) BioXcell Cat# BE0006
InVivoMAb anti-mouse CSF1R (Clone AFS98) BioXcell Cat# BE0213
InVivoMAb rat IgG2a isotype control, anti-trinitrophenol BioXcell Cat# BE0089
anti-mouse GFPT1 (EPR4854) Abcam Cat# AB125069
anti-mouse Fc Block (clone 2.4G2) BioXcell Cat# BE0307
anti--human CD45 APC-Fire810 (clone HI30) Biolegend Cat# 304076
anti-human CD14 FITC (clone M5E2) Biolegend Cat# 301804
anti-human CD172a PE (clone 15-414) Biolegend Cat# 372104
anti-human CD16 BV421 (clone 3G8) Biolegend Cat# 302038

Validation	All antibodies were obtained from commercial sources and validation data as well as detailed information can be found on the manufacturer's website.

Eukaryotic cell lines

Policy information about cell lines and Sex and Gender in Research

Cell line source(s)	OP9 cells were shared by Dr. Els Verhoeyen (Université Côte d'Azur, INSERM U1065, C3M).
Authentication	None of the cells lines were authenticated.
Mycoplasma contamination	Cells lines were not tested for Mycoplasma contamination.
Commonly misidentified lines (See ICLAC register)	*Name any commonly misidentified cell lines used in the study and provide a rationale for their use.*

Animals and other research organisms

Policy information about studies involving animals; ARRIVE guidelines recommended for reporting animal research, and Sex and Gender in Research

Laboratory animals	Wild-type C57BL/6J mice were purchased from Charles River or Janvier labs. Cx3cr1GFP (B6.Cg-Ptprca Cx3cr1tm1Litt/LittJ, Jax #008451), Clec9aiCre (B6J.B6N(Cg)-Clec9atm2.1(icre)Crs/J, Jax #025523), Lyz2Cre (B6.129P2-Lyz2tm1(cre)Ifo/J, Jax#004781) and R26TdTomato (B6.Cg-Gt(ROSA)26Sortm9(CAG-tdTomato)Hze/J, Jax #007909) mice used in this study were maintained under C57BL/6J background and originally purchased from the Jackson Laboratory. Ccr2CreERT2 (C57BL/6NTac-Ccr2tm2982(T2A –Cre7ESR1-T2A-mKate2)) mice38 were kindly provided by Dr. Burkhard Becher. Cx3cr1CreERT2 mice were reconstituted from cryopreserved sperm provided by Steffen Jung34 to Gwendalyn J. Randolph. Csf1rΔFIRE 26, Csf1flox/flox 77 and Cdh5CreERT2 78 mice were provided by Dr. David Hume, Dr. Sherry Abboud Werner and Dr. Ralf Adams (respectively) to Dr. Marc Bajénoff. Gfpt1 floxed mice (57BL/6N-Gfpt1<tm1c(EUCOMM)Wtsi>/H) were obtained from Mary Lyon Center (MRC, Harwell UK). Ubow mice were previously described42. Cx3cr1Cre (Tg(Cx3cr1-cre)MW126Gsat/Mmucd) mice were obtained from the GENSAT transgenic projet. Ms4a3FlpO,45 and R26FSF-tdTomato (B6.Cg-Gt(ROSA)26Sortm65.2(CAG-tdTomato)Hze/J, Jax#032864) mice were kindly shared by Dr. Florent Ginhoux and Dr. Elvira Mass. Experimental and control animals were co-housed, and littermate controls were used as often as possible. Animals of mixed sex and similar age were used within each cohort. Animals were used between the ages of 8 and 16 weeks throughout the study. Since we did not observe any sex-specific phenotypes, we decided to group data from male and female mice together in experiments where mice of both sexes were used. Mice were bred and housed in the Mediterranean Center of Molecular Medicine facility (INSERM U1065, Université Côte d'Azur), the PEMED facility (Pasteur campus, Université Côte d'Azur), the Washington University in Saint Louis animal facility, the University of Minnesota Medical School Research Animal Resources facility, or the Centre d'Immunologie de Marseille Luminy facility. An ambient temperature of ~20-23°C was maintained, with a 12/12-hour light/dark cycle and food available ad libitum. Animals were euthanized by cervical dislocation. Animal protocols required for experimentation other than organ collection were authorized by the French Ministry of Higher Education and Research upon approval of the local ethical committee (CIEPAL Azur) at Université Côte d'Azur, and by the Institutional Animal Care and Use Committee (IACUC) at Washington University in Saint Louis and University of Minnesota Medical School.
Wild animals	No wild animals were used in the study.
Reporting on sex	Animals of mixed sex and similar age were used within each cohort. Since we did not observe any sex-specific phenotypes, we decided to group data from male and female mice together in experiments where mice of both sexes were used.
Field-collected samples	No field-collected samples were used in the study.
Ethics oversight	Animal protocols required for experimentation other than organ collection were authorized by the French Ministry of Higher Education and Research upon approval of the local ethical committee (at Université Côte d'Azur and CIML, and by the Institutional Animal Care and Use Committee (IACUC) at Washington University in Saint Louis and University of Minnesota Medical School.

Note that full information on the approval of the study protocol must also be provided in the manuscript.

Plants

Seed stocks	*Report on the source of all seed stocks or other plant material used. If applicable, state the seed stock centre and catalogue number. If plant specimens were collected from the field, describe the collection location, date and sampling procedures.*
Novel plant genotypes	*Describe the methods by which all novel plant genotypes were produced. This includes those generated by transgenic approaches, gene editing, chemical/radiation-based mutagenesis and hybridization. For transgenic lines, describe the transformation method, the number of independent lines analyzed and the generation upon which experiments were performed. For gene-edited lines, describe the editor used, the endogenous sequence targeted for editing, the targeting guide RNA sequence (if applicable) and how the editor was applied.*
Authentication	*Describe any authentication procedures for each seed stock used or novel genotype generated. Describe any experiments used to assess the effect of a mutation and, where applicable, how potential secondary effects (e.g. second site T-DNA insertions, mosiacism, off-target gene editing) were examined.*

Flow Cytometry

Plots

Confirm that:

☒ The axis labels state the marker and fluorochrome used (e.g. CD4-FITC).

☒ The axis scales are clearly visible. Include numbers along axes only for bottom left plot of group (a 'group' is an analysis of identical markers).

☒ All plots are contour plots with outliers or pseudocolor plots.

☒ A numerical value for number of cells or percentage (with statistics) is provided.

Methodology

Sample preparation	Tissues were harvested after cervical dislocation and washed in PBS. Subcutaneous adipose tissue was digested using collagena A (3mg/mL) and DNAse I (100ug/mL). Splenocytes were prepared by gently crushing the spleen on a 70�m strainer in flow buffer (PBS containing 1% BSA and 2mM EDTA). Bone marrow cells were prepared by flushing femurs and tibias with flow buffer. Blood was drawn from the submandibular vein and collected in heparinized tubes. Leukocyte counts were quantified using a veterinary hematology analyzer (Exigo H400) or directly on the Cytek Aurora cytometer. Red blood cells were lysed from all single cell suspensions using BD Pharm Lyse lysing solution (BdBiosciences cat #555899). Cells were washed with PBS, washed in flow buffer and then stained. For intracellular staining, cells were fixed and permeabilized using Miltenyi Foxp3 staining buffer (cat #130-093-142). All antibodies were used 1/200. A list of all antibodies used is provided in Supplementary Table 2. Flow cytometry data were acquired using a BD FACS Canto II and a Cytek Aurora. All analyses, including unsupervised analyses, were performed using FlowJo software (Tree Star).
Instrument	BD CANTO II, Cytek Aurora, BD Aria II
Software	FACS DIVA<br>SpectroFlo<br>Flowjo
Cell population abundance	Purity of the sorted cell populations was not assessed immediatly after cell-sorting. Based on the phenotype of cells after 48 hours of culture, it is estimated that the purity was above 80%.
Gating strategy	Gating strategies are indicated in main or supplementary figures throughout the manuscript.

☒ Tick this box to confirm that a figure exemplifying the gating strategy is provided in the Supplementary Information.
