# Supplementary material for: CSF1R regulates monocyte subset differentiation and intracellular metabolism
Source: Nat Commun. 2026 Jul 4;17:8315. doi: 10.1038/s41467-026-75263-7 (PMC13470356; doi:10.1038/s41467-026-75263-7)
Supplement: Supplementary file 3 [file 41467_2026_75263_MOESM3_ESM.pdf]

nature portfolio

Peer Review File

CSF1R regulates monocyte subset differentiation and intracellular metabolism

Corresponding Author: Dr Stoyan Ivanov

This file contains all reviewer reports in order by version, followed by all author rebuttals in order by version.

Version 0:

Reviewer comments:

Reviewer #1

(Remarks to the Author)
1) You are giving an anti-CSF1R antibody and seeing monocytes disappear that are being examined as CSF1R+CD11b+. How do we know that AFS98 is not simply blocking binding to CSF1R or internalizing CSFR1.
2) How do we know that adding the anti-CSF1R antibody does not cause more firm adhesion of Ly6Clow monocytes to the vessel walls suggesting reduced numbers.
3) In the results the authors state that "We previously demonstrated that glucose metabolism plays a central role in controlling myelopoiesis and monocyte numbers 25. We demonstrated that preventing glucose entry, by administrating a Glut1-inhibitor, dramatically decreased monocyte CSF1R expression and their numbers in peripheral blood ref 25."

Its not clear why preventing glucose entry into monocytes would reduce CSFR1 and monocyte numbers if blocking CSF1R does not reduce monocytes.

4) The Lyz2 promoter would also delete GFPT in neutrophils which could impact on monocytes. There are more precise promoters than Lyz2

5) This reviewer would like to see that blood monocyte counts actually reflect total numbers of monocytes in the body. Redistribution from blood to tissue or better adhesion to the vessel wall could be responsible for a lot of this data.

6) Can the authors induce Ly6Chi monocytes to become Ly6Clo monocytes in vitro and can this be disrupted using CSF1R1 antibody

Reviewer #2

(Remarks to the Author)
Csf1r expression is intimately associated with the development and maintenance of the mononuclear phagocyte system and given the central role of monocytes and macrophages in physiology and pathophysiology, the study of the Csf1/ IL34 - Csf1r axis could hence yield important insights incl. potential novel intervention strategies. The study of Gallerand and colleagues that addresses Csf1r biology in monocytes is hence highly topical.
The authors start out by confirming earlier reports that monopoiesis is CSF1R-independent, whereas monocyte differentiation into the Ly6Clow subset is affected by CSF1R blockade. In this section, the authors furthermore provide some evidence that Csf1 binding to the Csf1r can confound detection with staining reagents and that monocyte surface CSF1R expression requires continuous protein translation and actin-dependent delivery to the cell membrane, rather than receptor endocytosis and recycling.
Using a Clec9a-Cre fate mapping model the authors then reveal that MDP-derived NCM are slightly less affected by CSF1R depletion, as compared to GMP-derived NCM.
The authors then study genetic models of Csf1r manipulation, including FIRE mice, confirming earlier findings and revealing that residual Csf1r expression in FIRE NCM is functional.
Finally the authors investigate a potential link of Csf1r signalling and metabolism. Specifically, they analyse intracellular monocyte metabolism used SCENITH and use a genetic approach to manipulate the hexosamine biosynthetic pathway (HBP). Their analysis of Lyz2cre x GFPT1fl/fl monocytes suggests that this metabolic alteration affects Csf1r expression.

Taken together, this study addresses an important topic, and the experiments seem generally well performed. However, the novelty and significance of the specific findings remain unclear. As such they also do not justify the title. Rather, the manuscripts read like a collection of anecdotal observations of which none has been pursued in sufficient depth to draw definitive conclusions.
While in the below we make some suggestions for improvement, the manuscript needs major restructuring and focus on the main message.

General comment

It is probably fair to assume that interference with Csf1r signalling will have an impact on the fate and survival of cells expressing the cytokine receptor, even if the latter cannot be revealed due to cellular dynamics and potential compensatory mechanisms. As such, the analysis of mice treated with the anti-Csf1r regimen can arguably be challenging, as also shown by the authors. It is hard to draw solid conclusions.

Specific comments

• Fig1E/ sup Fig1B. What is the CSF1R MFI in each of the monocyte subpopulations at baseline and after CSF1 incubation? Since differences in baseline expression or in downregulation after CSF1 exposure could affect the apparent recovery kinetics. If one population starts with a higher baseline MFI, it may take longer to return to that level, which could affect the interpretation of recycling rates.

• Fig 1F/G. The authors suggest that CSF1R is constantly translated to replace internalized receptors. However, if this is indeed the case, I would expect brefeldin A to have the same effect as Monensin because the newly translated protein will have to move through the ER. How do they explain that this is not the case? Statistics for the CSF1R MFI would be needed to strengthen the point. From the histograms, it is impossible to capture subtle differences of the treatments.
• A potential novelty of the study is the suggestion that the NCM population is affected due to the failed differentiation of CM in the absence of CSF1R. However, the NCM half-life is not examined. It therefore cannot be ruled out that the shift in CM\NCM balance is due to shorter survival of NCM together with faster exit of CM from the BM to the blood, which maintains the high level of CM. Likewise, maybe CM half-life is reduced, preventing them from differentiation.
• Supp Fig2B/C. The depletion of NCM in general and the related canonical markers is not clear from the UMAP; statistical representation should help. Do the remaining NCM cells express lower levels of Treml4 and CD11c, or is the reduction due to a lower number of NCM cells?
• What gating strategies were used to identify monocyte populations in the spleen and BM? Does the trend toward reduced monocytes in the BM primarily reflect a decrease in Ly6Chi monocytes?
• Figure 3. What is the gating strategy used for the monocyte populations?
• Is the turnover of tdTom+ MDP-derived versus tdTom- GMP-derived CM and NCM comparable? Analyzing all monocyte populations together may be misleading, as Fig. 1E indicates that different subsets exhibit distinct CSF1R turnover rates. A side-by-side analysis of GMP- and MDP-Mo, using Clec9a-Cre: and Ms4a3Cre:TdTom mice would here be warranted.
• If the lack of CSF1R on Ly6Chi Fire mice does not prevent the transition of Ly6Chi to Ly6Clo monocytes, doesn't this suggest that the reduction of Ly6Clo cells observed in the other models results from impaired survival of this population in the absence of CSF1R signaling, rather than from defective differentiation, as proposed?
• Fig5A. mTOR phosphorylation is also unchanged in Ly6Clo according to the statistics.
• According to the findings up to Figure 5, CSF1R blockade primarily impacts the NCM population by reducing their numbers. However, the group's previous work (ref.# 25) demonstrated that glucose metabolism is critical for the survival and CSF1R regulation of CM, while playing only a limited role in NCM. In light of the current findings showing that CSF1R inhibition or deletion increases the relative proportion of CM and decreases NCM, what was the rationale for focusing on glucose metabolism? Moreover, how do you reconcile the observation that NCM, rather than CM, are preferentially affected in this genetic model?
• The authors assess rearrangement efficiency in BMDM cultures and use this as a proxy for a monocyte analysis. However, this is unacceptable. A genomic analysis of Lyz2cre x GFPT1fl/fl monocytes is required. Lyz2cre is known to have variable penetrance in monocytes (PMID24077561). Moreover, in the given GFPT1fl/fl configuration, the authors probably also have GFPT1 heterozygote cells, which will confound the conclusion.
• Finally, since Lyz2cre also highly affects neutrophils, were the general blood cell populations of Lyz2cre x GFPT1fl/fl mice examined?
• When mentioning the monocyte trajectories originating from GMP and MDP, please also cite the study by Yanez et al. (PMC5726802), which was arguably the first to reveal the dichotomy.

Minor comments

• Please check for textual inconsistencies: See, for instance, page 5, end of 2nd paragraph: 'CSF1R reappearance at the cell surface was comparable between GMP and MDP-derived monocytes (Figure 3E). Therefore, MDP-derived monocytes, while displaying higher CSF1R expression, appeared less dependent on CSF1R signaling.'

Reviewer #3

(Remarks to the Author)
This manuscript, by Gallerand et al, explore the role of CSFR1 in regulating monocyte subset homeostasis. The authors also

explore the metabolism of monocytes, particularly the hexosamine pathway.
Overall, the study is well-designed and thorough. The metabolic piece of the paper is a minor part of the paper, although likely the most novel, as the metabolism of monocytes is not well-studied.

A few questions remain:
Figure 1c: this is a loss of all monocytes, correct? What is the percentage of monocytes coming back at 72h? Are these mostly classical monocytes? Is there a difference in their phenotype between those of isotype-treated (i.e., more immature, perhaps)?

Figure 2H: the authors suggest that the loss of intermediate (Ly6C+TREML4+) monocytes with AFS98 is due to diminished commitment of these intermediate cells. An alternative hypothesis is simply that these cells are also depleted by the AFS98. How do the other antibodies (W19330E and W19330C) impact this intermediate population?

Figure 5: LysM-cre is often not very efficient in reducing gene expression in monocytes. What percentage of expression of GFPT1 was lost in classical, intermediate and nonclassical monocytes in these mice at baseline?

Figure 5: The most novel aspect of this work is the metabolic work in Figure 5, although the loss of GFPT1 doesnt have a huge impact it seems. An analysis of Ly6C low and Ly6Chi monocyte metabolism, with and without the AFS98 Rx, using Seahorse, would be an important addition to this study.

Overall: since CSFR1 inhibitory antibodies have been approved for use in humans, it is very important to measure the glucose metabolic pathways in human classical and nonclassical monocytes with CSFR1 inhibition. If not possible to obtain the antibody, then at least baseline glucose and mitochondrial measures should be shown.

Version 1:

Reviewer comments:

Reviewer #2

(Remarks to the Author)
The authors have included new data and revised the manuscript in accordance with our suggestions.
We still find it somewhat difficult to follow the central story and identify the take-home messages. Emphasizing these, at the end of the intro or start of the discussion could help.
The authors also might want to consider to move some of the more technical details to the supplement. In fact they could move the FIRE mice to there to streamline the main text. As previously noted, this manuscript reveals a number of interesting fact, although not all are entirely new. It has f.i. been reported that both GMP- and MDP-Mo give rise to NCM (see ref# 10); This should be acknowledged in line # 339. While I am not a metabolome expert, I felt this part somewhat underdeveloped, and the title is hence maybe not entirely appropriate.

As asked by the editor, I also went over the author's response to the critique of reviewer 1. To my mind, the authors also answered here well and sufficiently. Point 1 was a misunderstanding, and the other points are addressed by new data.
I think this study is ready to be published.

Reviewer #3

(Remarks to the Author)
The authors performed a significant number of innovative experiments to address my concerns. My questions and comments have been fully addressed.

Open Access This Peer Review File is licensed under a Creative Commons Attribution 4.0 International License, which permits use, sharing, adaptation, distribution and reproduction in any medium or format, as long as you give appropriate credit to the original author(s) and the source, provide a link to the Creative Commons license, and indicate if changes were made.
In cases where reviewers are anonymous, credit should be given to 'Anonymous Referee' and the source.
The images or other third party material in this Peer Review File are included in the article's Creative Commons license, unless indicated otherwise in a credit line to the material. If material is not included in the article's Creative Commons license and your intended use is not permitted by statutory regulation or exceeds the permitted use, you will need to obtain permission directly from the copyright holder.
To view a copy of this license, visit https://creativecommons.org/licenses/by/4.0/

We thank the reviewers for their careful reading of our manuscript and insightful comments. We agree that the manuscript would benefit from a major restructuring, and therefore we reorganized the revised version to focus on our major original observations as follows:

1. We reduced the technical part of the manuscript focusing on epitope masking.
2. We integrated newly generated data demonstrating that CSF1R plays a central role in monocyte differentiation and the generation of the $Ly6C^{low}$ subset.
3. We included additional data investigating differential CSF1R regulation in GMP- and MDP-derived blood monocytes including its synthesis and recycling rate.
4. We strengthened the metabolic section of the manuscript that provides new and important insights about the role of HBP in monocyte biology via CSF1R regulation.
5. We included data on the regulation of human monocyte metabolism by receptor tyrosine kinase signaling to translate our observations obtained in pre-clinical models.

We addressed all reviewer comments below and the changes in the revised manuscript are indicated in blue.

Reviewer #1 (Remarks to the Author):

1) You are giving an anti-CSF1R antibody and seeing monocytes disappear that are being examined as CSF1R+CD11b+. How do we know that AFS98 is not simply blocking binding to CSF1R or internalizing CSFR1.

We believe the reviewer is referring to our results shown in Fig. 1B in the original manuscript showing a dramatic reduction in blood monocytes after AFS98 injection, examined as $CSF1R^{+}CD11b^{+}$ cells. The reviewer seems to have missed the point that we indeed subsequently investigated whether this monocyte disappearance was not simply a consequence of epitope masking or receptor internalization. The experiments shown in Fig. 1C-G and Supplementary Figure 1 of the original manuscript in fact demonstrate that epitope masking and receptor recycling is occurring and we therefore developed an alternative gating strategy that does not depend on CSF1R detection (new **Figure 2A-B**). Using this alternative gating strategy, we performed the CSF1R blocking experiments again and observed that CSF1R blocking leads to a specific reduction in $Ly6C^{low}$ monocytes (new **Figure 2C**). Furthermore, using an antibody whose epitope is not masked by AFS98 (W19330C), we confirmed these results (Figure 2D-E of the original manuscript). While this part on epitope masking is important for data interpretation and seems able to explain previously published contradictory results, it also seems to be confusing and to distract from more important messages that we would like to convey. We therefore restructured the manuscript and reduced this part, as is also suggested by Reviewer 2, and focus more on the metabolic part of our study, as suggested by Reviewer 3.

2) How do we know that adding the anti-CSF1R antibody does not cause more firm adhesion of Ly6Clow monocytes to the vessel walls suggesting reduced numbers.

We would like to thank the reviewer for this interesting question. Adhesion to endothelium promotes CSF1 binding and CSF1R signaling in $Ly6C^{low}$ monocytes[1]. In line with this finding, we detected lower CSF1R expression on monocytes that were detached from endothelial cells by intravenous injection of anti-CD11a blocking antibodies, due to higher CSF1 binding (new **Supplementary Figure 2F**). We also performed CD11a blockade in control and anti-CSF1R-treated mice to determine whether anti-CSF1R affected adhesion of $Ly6C^{low}$ monocytes to blood vessels (new **Supplementary Figure 2G**). Of note, CD11a blockade in AFS98-treated mice did not restore the levels of blood $Ly6C^{+}$ $Treml4^{+}$ and $Ly6C^{low}$ $Treml4^{+}$ monocytes in comparison to control animals (new **Supplementary Figure 2G**). These newly generated data are included in the revised version of the manuscript.

3) In the results the authors state that "We previously demonstrated that glucose metabolism plays a central role in controlling myelopoiesis and monocyte numbers 25. We demonstrated that preventing glucose entry, by administrating a Glut1-inhibitor, dramatically decreased monocyte CSF1R expression and their numbers in peripheral blood ref 25."

Its not clear why preventing glucose entry into monocytes would reduce CSFR1 and monocyte numbers if blocking CSF1R does not reduce monocytes.
Prolonged CSF1R blockade reduces Ly6C^{low} numbers. Our previous work demonstrated that preventing Glut1-dependent glucose entry in monocytes was associated with decreased monocyte numbers and membrane CSF1R expression. However, in this previous work we did not provide experimental data demonstrating a causal link between CSF1R regulation by glucose metabolism and monocyte subsets survival.

4) The Lyz2 promoter would also delete GFPT in neutrophils which could impact on monocytes. There are more precise promoters than Lyz2
Lyz2 mediated deletion of GFPT1 did not impact blood neutrophil numbers (**Supplementary Figure 6E**). Thus, the regulation of CSF1R and the decreased frequency of blood Ly6C^{low} monocytes unlikely results from GFPT1-deletion in neutrophils.

5) This reviewer would like to see that blood monocyte counts actually reflect total numbers of monocytes in the body. Redistribution from blood to tissue or better adhesion to the vessel wall could be responsible for a lot of this data.
We agree with the reviewer's comment that this is an important question, which we have now addressed. We performed a detailed analysis of blood monocyte content in mice treated with anti-CSF1R and anti-CD11a Abs (see response to Reviewer#1 Question#2). We also tested the presence of Ly6C^{low} monocytes in the tissue vasculature by injecting control and anti-CSF1R treated mice intravenously with FITC-conjugated anti-CD45 to distinguish monocytes in the tissue from those that had not extravasated (**new Figures 2G-H**). Taking subcutaneous adipose tissue as an example, we found that AFS98 treatment depleted all CD45i.v.$^{+}$ Ly6C^{low} monocytes (**new Figure 2G-H**), indicating that adhesion of Ly6C^{low} monocytes to blood vessels in tissues was not the cause for their disappearance from blood.

6) Can the authors induce Ly6Chi monocytes to become Ly6Clo monocytes in vitro and can this be disrupted using CSF1R1 antibody

We appreciate this crucial question. We performed several experiments to test whether CSF1R could be involved in monocyte differentiation. Using Ccr2^{CreERT2}; R26^{tdTomato} mice, allowing to efficiently label Ly6C^{high} monocytes with tdTomato, we were able to track their differentiation into Ly6C^{low} cells in blood over time (**new Figures 3E-G**). The numbers of both Ly6C^{+} Treml4^{+} tdTomato^{+} and Ly6C^{low} Treml4^{+} tdTomato^{+} monocytes, but not Ly6C^{+} Treml4^{-} tdTomato^{+} cells, were diminished after AFS98 administration demonstrating a central role of CSF1R signaling in monocyte conversion (**new Figures 3F-G**). Similar observations were obtained in mice treated initially with clodronate (to deplete all blood monocytes) and subsequently with AFS98 (to investigate the role of CSF1R signaling on monocyte conversion) (**new Supplementary Figures 3C-D**). In a highly complementary *in vivo* approach, we took advantage of Ubow$^{tg/+}$ mice[2]. To target monocytes, we used Cx3cr1^{Cre}; Ubow$^{tg/+}$ mice. Bone marrow of these mice was extracted and transferred into lethally irradiated Cdh5^{CreERT2}; Csf1$^{fl/fl}$ (allowing to delete CSF1 on endothelial cells[1]) and control mice (**new Figures 3J-L**). We observed that the frequency of cells expressing YFP or CFP among Ly6C^{int} and Ly6C^{low} monocytes was lower in chimeric mice deficient for CSF1 on endothelial cells compared to control animals (**new Figures 3J-L**). Finally, we also developed an *in vitro* approach to explore

the differentiation of Ly6C^{+} Treml4^{+} monocytes into Ly6C^{low} Treml4^{+} cells as suggested by the reviewer. We cell sorted both subsets and cultured them on OP9 feeder cells in the presence of recombinant CSF1 or a combination of CSF1 and AFS98 (**new Figure 3H**). We noticed that AFS98 addition to cell culture affected the viability of both Ly6C^{+} Treml4^{+} and Ly6C^{low} Treml4^{+} monocytes and also decreased the conversion of Ly6C^{+} Treml4^{+} into Ly6C^{low} Treml4^{+} monocytes (**new Figure 3I**). This culminated in a decreased Ly6C^{low}:Ly6C^{+} monocyte ratio after AFS98 addition (**new Figure 3I)**.

Reviewer #2 (Remarks to the Author):

Csf1r expression is intimately associated with the development and maintenance of the mononuclear phagocyte system and given the central role of monocytes and macrophages in physiology and pathophysiology, the study of the Csf1/ IL34 - Csf1r axis could hence yield important insights incl. potential novel intervention strategies. The study of Gallerand and colleagues that addresses Csf1r biology in monocytes is hence highly topical.
The authors start out by confirming earlier reports that monopoiesis is CSF1R-independent, whereas monocyte differentiation into the Ly6Clow subset is affected by CSF1R blockade. In this section, the authors furthermore provide some evidence that Csf1 binding to the Csf1r can confound detection with staining reagents and that monocyte surface CSF1R expression requires continuous protein translation and actin-dependent delivery to the cell membrane, rather than receptor endocytosis and recycling.
Using a Clec9a-Cre fate mapping model the authors then reveal that MDP-derived NCM are slightly less affected by CSF1R depletion, as compared to GMP-derived NCM.
The authors then study genetic models of Csf1r manipulation, including FIRE mice, confirming earlier findings and revealing that residual Csf1r expression in FIRE NCM is functional. Finally the authors investigate a potential link of Csf1r signalling and metabolism. Specifically, they analyse intracellular monocyte metabolism used SCENITH and use a genetic approach to manipulate the hexosamine biosynthetic pathway (HBP). Their analysis of Lyz2cre x GFPT1fl/fl monocytes suggests that this metabolic alteration affects Csf1r expression.

Taken together, this study addresses an important topic, and the experiments seem generally well performed. However, the novelty and significance of the specific findings remain unclear. As such they also do not justify the title. Rather, the manuscripts read like a collection of anecdotal observations of which none has been pursued in sufficient depth to draw definitive conclusions.
While in the below we make some suggestions for improvement, the manuscript needs major restructuring and focus on the main message.
We would like to thank the reviewer for the careful reading and examination of our manuscript and for the important questions that she/he brought up. We agree with the reviewer and therefore re-structured the manuscript to tone down technical aspects and instead focus on novel points to develop them further.

General comment

It is probably fair to assume that interference with Csf1r signalling will have an impact on the fate and survival of cells expressing the cytokine receptor, even if the latter cannot be revealed due to cellular dynamics and potential compensatory mechanisms. As such, the analysis of mice treated with the anti-Csf1r regimen can arguably be challenging, as also shown by the authors. It is hard to draw solid conclusions.
Since monocytes and macrophages are key players involved in multiple infections and inflammatory diseases, a better understanding of the molecular mechanisms regulating their numbers and functions might open novel therapeutic windows or fine-tune current therapies.

CSF1R blocking Ab (Axatilimab) was recently approved to treat patients with chronic graft-versus-host disease[3]. Therefore, a better understating of how CSF1R is regulated on blood monocytes depending on their origin from GMPs or MDPs appears important. Furthermore, to apprehend how precisely glucose metabolism controls monocyte CSF1R expression will provide crucial fundamental knowledge. Thus, we believe that the observations reported in our manuscript will be important for the field.

Specific comments

• Fig1E/ sup Fig1B. What is the CSF1R MFI in each of the monocyte subpopulations at baseline and after CSF1 incubation? Since differences in baseline expression or in downregulation after CSF1 exposure could affect the apparent recovery kinetics. If one population starts with a higher baseline MFI, it may take longer to return to that level, which could affect the interpretation of recycling rates.

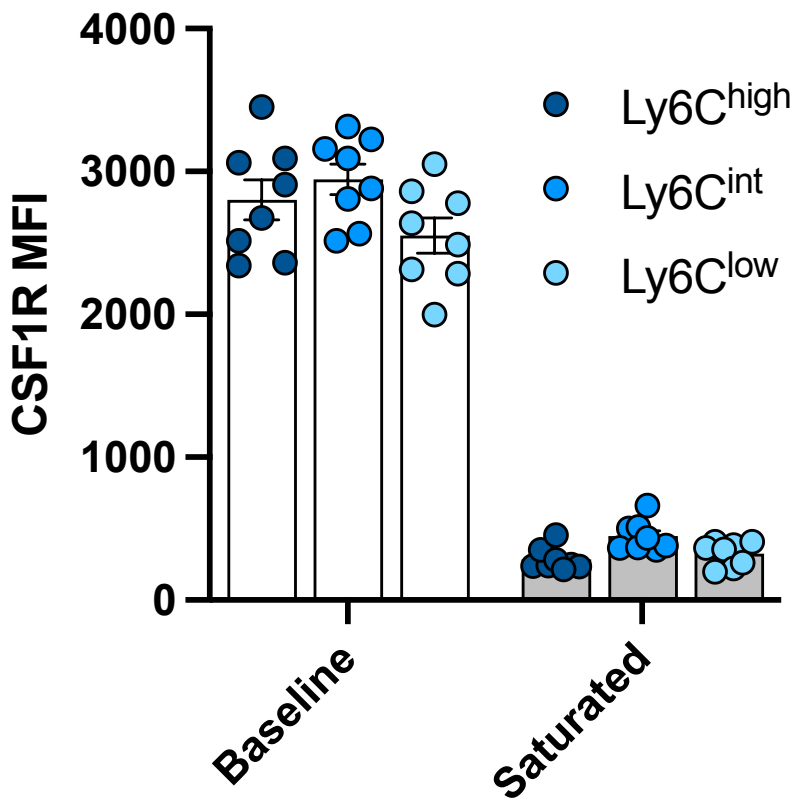

Response Figure 1. CSF1R expression by monocyte subsets at baseline and after incubation with CSF1 (saturated).

CSF1R MFI of different monocyte subsets at baseline and after saturation are shown in **Response Figure 1**. We agree with the reviewer that differences in MFI should be taken into account in the recycling assay, and the reviewer is right to point out that Ly6C^{low} monocytes might recover a higher percentage of their baseline expression since their baseline is inferior to the other subsets. For this reason, we changed the representation of our data back to raw MFI to avoid this bias (**new Figure 1B**). Our conclusions remain unchanged nonetheless, as CSF1R MFI increased faster in Ly6C^{low} monocytes compared to the Ly6C^{high} subset (**new Figure 1B**).

• Fig 1F/G. The authors suggest that CSF1R is constantly translated to replace internalized receptors. However, if this is indeed the case, I would expect brefeldin A to have the same effect as Monensin because the newly translated protein will have to move through the ER. How do they explain that this is not the case? Statistics for the CSF1R MFI would be needed to strengthen the point. From the histograms, it is impossible to capture subtle differences of the treatments.

Brefeldin A affects protein transport at an earlier stage than monensin, which could explain this discrepancy over the short incubation period used in our experiments. We discussed the specificity of each approach in the revised manuscript, p.3 l.119-121. We agree with the reviewer and therefore added proper statistical analysis to this figure to improve the reader's interpretation. In fact, we repeated this experiment several times and upon detailed analysis we noticed that monocyte subsets showed a different sensitivity to treatment with brefeldin A, monensin and harringtonin. We found that while inhibition of protein trafficking or synthesis for 45minutes inhibited up to 75% of CSF1R expression on Ly6C^{high} Treml4^{-} monocytes, only up to 50% inhibition was observed on Ly6C^{low} Treml4^{+} monocytes (**new Figures 1 C-E**). This finding supports the idea that different CSF1R regulation across monocyte subsets is tied to different reliance upon this growth factor receptor.

• A potential novelty of the study is the suggestion that the NCM population is affected due to the failed differentiation of CM in the absence of CSF1R. However, the NCM half-life is not examined. It therefore cannot be ruled out that the shift in CM\NCM balance is due to shorter

survival of NCM together with faster exit of CM from the BM to the blood, which maintains the high level of CM. Likewise, maybe CM half-life is reduced, preventing them from differentiation.
We appreciate this crucial question. We performed several experiments to evaluate the role of CSF1R on monocyte differentiation and lifespan. Using Ccr2^{CreERT2}; R26^{tdTomato} mice, allowing to efficiently label Ly6C^{high} monocytes with tdTomato, we were able to track their differentiation to Ly6C^{low} cells in blood over time (**new Figures 3E-G**). The numbers of both Ly6C^{+} Treml4^{+} tdTomato^{+} and Ly6C^{low} Treml4^{+} tdTomato^{+} monocytes, but not Ly6C^{+} Treml4^{-} tdTomato^{+} cells, were diminished after AFS98 administration demonstrating a central role of CSF1R signaling in monocyte differentiation (**new Figures 3F-G**). Similar observations were obtained in mice treated initially with clodronate (to deplete all blood monocytes) and subsequently with AFS98 (to investigate the role of CSF1R signaling on monocyte conversion) (**new Supplementary Figures 3C-D**). In a highly complementary *in vivo* approach, we took advantage of Ubow$^{tg/+}$ mice[2]. To target monocytes, we used Cx3cr1^{Cre}; Ubow$^{tg/+}$ mice. Bone marrow of these mice was extracted and transferred into lethally irradiated Cdh5^{CreERT2}; Csf1$^{fl/fl}$ (allowing to ablate CSF1 on endothelial cells[1]) and control mice (**new Figures 3J-L**). We observed that the frequency of cells expressing YFP or CFP among Ly6C^{int} and Ly6C^{low} monocytes was lower in chimeric mice deficient for CSF1 on endothelial cells compared to control animals (**new Figures 3J-L**). Finally, we also developed an *in vitro* approach to explore the conversion of Ly6C^{+} Treml4^{+} monocytes into Ly6C^{low} Treml4^{+} cells as suggested by the reviewer. We cell sorted both subsets and cultured them on OP9 feeder cells in the presence of recombinant CSF1 or a combination of CSF1 and AFS98 (**new Figure 3H**). We noticed that AFS98 addition to cell culture affected the viability of both Ly6C^{+} Treml4^{+} and Ly6C^{low} Treml4^{+} monocytes and also decreased the differentiation of Ly6C^{+} Treml4^{+} into Ly6C^{low} Treml4^{+} monocytes (**new Figure 3I**). This culminated in a decreased Ly6C^{low}:Ly6C^{+} monocyte ratio after AFS98 addition (**new Figure 3I)**.

• Supp Fig2B/C. The depletion of NCM in general and the related canonical markers is not clear from the UMAP; statistical representation should help. Do the remaining NCM cells express lower levels of Treml4 and CD11c, or is the reduction due to a lower number of NCM cells?
We agree with the reviewer that quality of the previous UMAP representation was poor. We have performed a new analysis and updated the panels, on which expression of Treml4 and CD11c are easier to see (**new Supplementary Figures 2B-C**). Based on this experiment and all others performed throughout the manuscript, we conclude that the loss of NCM is due to lower numbers of this population and not a shift in expression of surface markers.

• What gating strategies were used to identify monocyte populations in the spleen and BM? Does the trend toward reduced monocytes in the BM primarily reflect a decrease in Ly6Chi monocytes?
Indeed, the trend towards reduced monocytes in BM reflects a decrease of the Ly6C^{high} subset as monocytes were gated as CD11b^{+} Ly6G^{-} Ly6C^{high} cells in BM. Spleen was analyzed in Cx3cr1$^{GFP/+}$ mice, and spleen Ly6C^{high} monocytes were identified as CD45^{+} CD11b^{+} Ly6C^{high} CX3CR1^{GFP+} cells. We clarified this point in the manuscript and included the gating strategies in the corresponding figure legends.

• Figure 3. What is the gating strategy used for the monocyte populations?
Total monocytes were identified as in Figure 2A or using a similar gating strategy that doesn't include CSF1R as an identification marker. We have adequate information relative to the gating strategy used in the relevant figure legends.

• Is the turnover of tdTom+ MDP-derived versus tdTom- GMP-derived CM and NCM

comparable? Analyzing all monocyte populations together may be misleading, as Fig. 1E indicates that different subsets exhibit distinct CSF1R turnover rates. A side-by-side analysis of GMP- and MDP-Mo, using Clec9a-Cre: and Ms4a3Cre:TdTom mice would here be warranted.

We agree with the reviewer's comment and improved Figure 5 accordingly. First, we used unsupervised analyses of tdTomato-labeled monocytes from Clec9a^{tdTomato} and Ms4a3^{tdTomato} mice to demonstrate that while the latter efficiently tracks GMP-derived monocytes[4], Clec9a^{tdTomato} mice allow to track MDP-derived monocytes identified by recently-described surface markers[5] (**new Figures 5A-E**). Based on these results, we used Clec9a^{tdTomato} mice to simultaneously track MDP-derived tdTomato^{+} and GMP-derived tdTomato^{-} monocytes (**new Figure 5G**). While tdTomato^{+} and tdTomato^{-} monocytes showed different sensitivity to CSF1R blockade, Ly6C^{low} Treml4^{+} monocytes from both lineages were depleted by AFS98 (**new Figures 5G-I**). Also using Clec9a^{tdTomato} mice, we compared CSF1R turnover rates between tdTomato^{+} and tdTomato^{-} monocytes of each subset and did not observe any lineage-specific differences (**new Figure 5J**). Finally, as requested by the reviewer, we performed a side-by-side analysis of this readout using both Clec9a^{tdTomato} and Ms4a3^{tdTomato} mice and also did not observe significant differences between tdTomato^{+} monocytes from each model (**Response Figure 2**). While these results were not included in the revised manuscript due to low number of experimental replicates, they support our initial approach and conclusions.

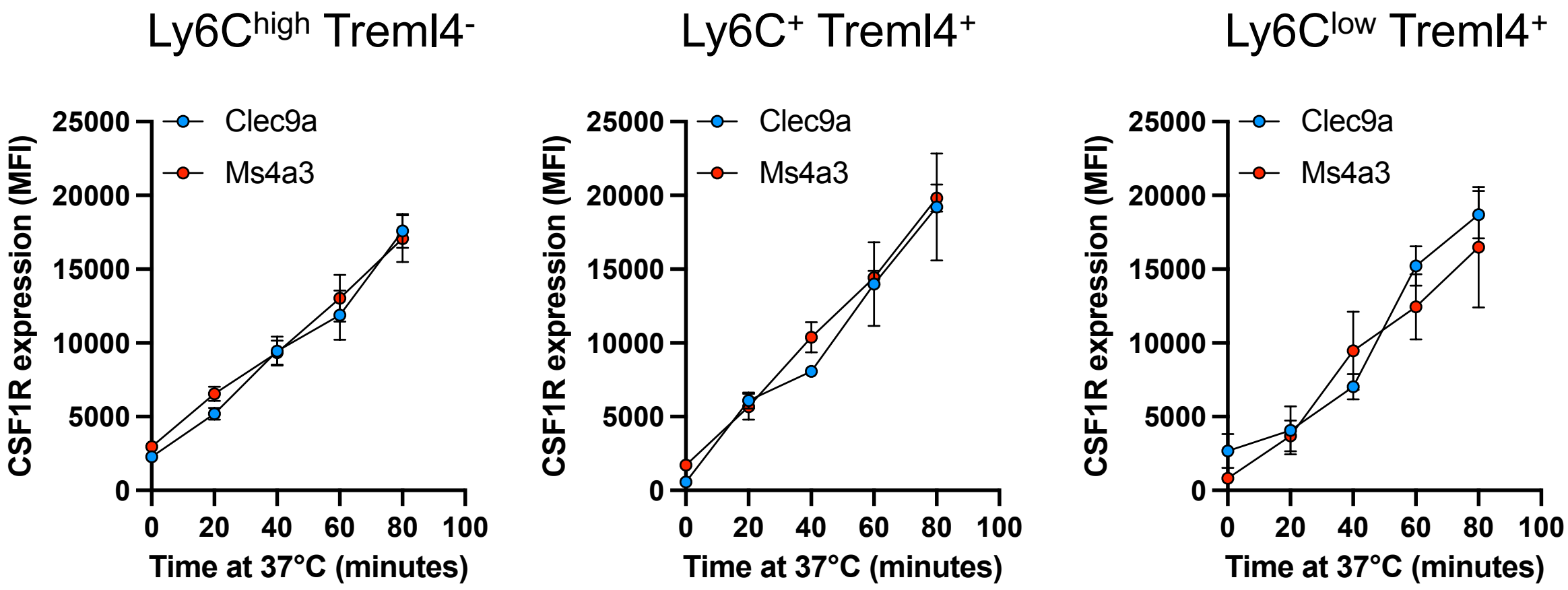

Response Figure 2. CSF1R turnover was analyzed in each tdTomato-labeled monocyte subset from Clec9a and Ms4a3 fate-mapper mice.

• If the lack of CSF1R on Ly6Chi Fire mice does not prevent the transition of Ly6Chi to Ly6Clo monocytes, doesn't this suggest that the reduction of Ly6Clo cells observed in the other models results from impaired survival of this population in the absence of CSF1R signaling, rather than from defective differentiation, as proposed?

While CSF1R$^{FIRE/FIRE}$ mice provide important insights about monocyte and macrophage biology, this model remains challenging to fully explain. One hypothesis is that CSF1R expression is maintained on monocytes from CSF1R$^{FIRE/FIRE}$ mice but its detection is hindered by CSF1-driven epitope masking. In this case, CSF1R could remain partially detectable on Ly6C^{low} Treml4^{+} but not Ly6C^{high} Treml4^{-} monocytes due to their faster CSF1R turnover. A recent study used a novel CSF1R kinase dead mouse model, displaying a point mutation in CSF1R (E631K)[6]. These mice have a compromised myeloid cell compartment and reduced blood monocyte numbers in comparison to control animals. Yet their blood monocytes (both Ly6C^{high} et Ly6C^{low} subsets) are present with a conserved ratio. These data suggest that monocyte conversion could occur in the absence of CSF1R signaling. In dendric cells it was demonstrated that CSF1R signaling compensates for FLT3 loss [7]. The opposite might be occurring in monocytes and could explain why Ly6C^{low} monocytes are present in CSF1R$^{FIRE/FIRE}$ mice. Therefore, and as indicated by the reviewer, the precise function of

CSF1R in monocyte biology is an important topic that requires the development of novel models and further investigation. Our study, while not providing a definitive and complete answer of the role of CSF1R in monocyte biology, brings up important and novel information that will be essential for the field. Since CSF1R blocking Ab is currently approved and used in patients and several clinical trials are currently ongoing, this work shall further provide knowledge about the impact of CSF1R signaling and glucose metabolism on monocyte biology.

• Fig5A. mTOR phosphorylation is also unchanged in Ly6Clo according to the statistics.
We changed our phrasing to properly depict this phenotype (p.8 l.366). The corresponding panel now also depicts exact p-value.

• According to the findings up to Figure 5, CSF1R blockade primarily impacts the NCM population by reducing their numbers. However, the group's previous work (ref.# 25) demonstrated that glucose metabolism is critical for the survival and CSF1R regulation of CM, while playing only a limited role in NCM. In light of the current findings showing that CSF1R inhibition or deletion increases the relative proportion of CM and decreases NCM, what was the rationale for focusing on glucose metabolism? Moreover, how do you reconcile the observation that NCM, rather than CM, are preferentially affected in this genetic model?
In our previous work[8] we showed that Glut-1 inhibition rapidly reduced CSF1R expression on monocytes, then triggered a loss of CM and this was followed by a loss of NCM over a longer period of treatment. This sequence of events is compatible with our interpretation that CSF1R expression on CM regulates their differentiation to NCM, and its expression on NCM regulates their own survival. Moreover, we quantified all monocyte subsets in Lyz2$^{\Delta GFPT1}$ mice (only proportions were shown previously) and noticed that numbers of the Ly6C^{high} Treml4^{-} subset were augmented in this model (**new Figure 6F**). Numbers of the Ly6C^{low} Treml4^{+} subset were unaffected (**new Figure 6F**), indicating that our previous observation that their proportions augmented actually reflected accumulation of the Ly6C^{high} Treml4^{-} subset. While basal CSF1R expression was decreased in all GFPT1-deficient monocyte subsets (**new Figure 6G**), their CSF1R turnover was accelerated (**new Figure 6H and new Supplementary Figures 6G-H**), potentially as a compensation mechanism. The fact that NCM are less affected in Lyz2$^{\Delta GFPT1}$ mice is likely due to their longer lifespan.

• The authors assess rearrangement efficiency in BMDM cultures and use this as a proxy for a monocyte analysis. However, this is unacceptable. A genomic analysis of Lyz2cre x GFPT1fl/fl monocytes is required. Lyz2cre is known to have variable penetrance in monocytes (PMID24077561). Moreover, in the given GFPT1fl/fl configuration, the authors probably also have GFPT1 heterozygote cells, which will confound the conclusion.

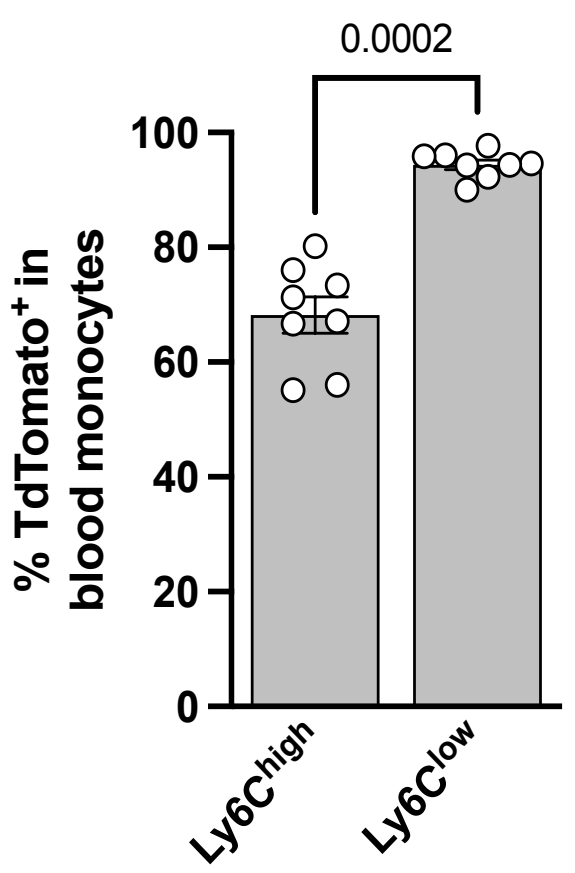

Response Figure 3. Lyz2^{Cre} expression in blood monocytes, measured using the Ai9 reporter.

We agree with the reviewer's comment and aimed to validate *Gfpt1* invalidation in monocyte subsets. We also found using Lyz2^{cre} x R26^{tdTomato}-reporter mice that Lyz2^{cre} expression is variable in Ly6C^{high} monocytes (**Response Figure 3**). However, Lyz2^{cre} is expressed in around 95% of Ly6C^{low} monocytes. Still, recombination efficiency is very high in the R26^{tdTomato} reporter transgene, and this might not reflect recombination efficiency of *Gfpt1*. Therefore, we performed intracellular flow cytometry analysis of GFPT1 expression in control and Lyz2^{cre} x GFPT1$^{fl/fl}$ mice, but those attempts remained unfruitful. Thus, we cell-sorted splenic Ly6C^{high} Treml4^{-}, Ly6C^{+} Treml4^{+} and Ly6C^{low} Treml4^{+} monocytes and performed a PCR analysis (**new Supplementary Figure 6D**). Our data revealed that Lyz2^{cre} efficiently deleted GFPT1 expression in all monocyte subsets (**new**

Supplementary Figure 6D). The deletion efficiency varied from nearly 60% in Ly6C^{high} Treml4^{-} monocytes to 70% in Ly6C^{+} Treml4^{+} and Ly6C^{low} Treml4^{+} cells (**new Supplementary Figure 6D**).

• Finally, since Lyz2cre also highly affects neutrophils, were the general blood cell populations of Lyz2cre x GFPT1fl/fl mice examined?
Blood neutrophil numbers were not affected by GFPT1 deletion (**new Supplementary Figure 6E**).

• When mentioning the monocyte trajectories originating from GMP and MDP, please also cite the study by Yanez et al. (PMC5726802), which was arguably the first to reveal the dichotomy.
We included the suggested reference in the revised version of the manuscript (p.2 l.66).

Minor comments

• Please check for textual inconsistencies: See, for instance, page 5, end of 2nd paragraph: 'CSF1R reappearance at the cell surface was comparable between GMP and MDP-derived monocytes (Figure 3E). Therefore, MDP-derived monocytes, while displaying higher CSF1R expression, appeared less dependent on CSF1R signaling.
We sincerely apologize for the inconsistencies that was corrected in the revised version of the manuscript.

Reviewer #3 (Remarks to the Author):

This manuscript, by Gallerand et al, explore the role of CSFR1 in regulating monocyte subset homeostasis. The authors also explore the metabolism of monocytes, particularly the hexosamine pathway.
Overall, the study is well-designed and thorough. The metabolic piece of the paper is a minor part of the paper, although likely the most novel, as the metabolism of monocytes is not well-studied.
We would like to thank the reviewer for the positive suggestion. As suggested by the reviewer we performed Seahorse analyses to better define the impact of GFPT1 deletion on monocyte and macrophage intracellular metabolism (**new Supplementary Figure 6I**).

A few questions remain:
Figure 1c: this is a loss of all monocytes, correct? What is the percentage of monocytes coming back at 72h? Are these mostly classical monocytes? Is there a difference in their phenotype between those of isotype-treated (i.e., more immature, perhaps)?
Figure 1C (first version of the manuscript) reflected mostly CSF1R epitope masking, but also in part the disappearance of Ly6C^{low} monocytes (as shown in the rest of the manuscript). As several reviewers pointed out, this part of the manuscript confuses our main message, and we decided to remove technical aspects from Figure 1 to make it easier to follow.

Figure 2H: the authors suggest that the loss of intermediate (Ly6C+TREML4+) monocytes with AFS98 is due to diminished commitment of these intermediate cells. An alternative hypothesis is simply that these cells are also depleted by the AFS98. How do the other antibodies (W19330E and W19330C) impact this intermediate population?
These clones are only sold as 25 or 100 µg formats containing sodium azide (Biolegend) and are therefore regrettably not suitable for *in vivo* experiments. To improve the clarity of the manuscript, this figure was removed in the revised version. As described above (please see our reply to Reviewers 1 and 2), we used several complementary models to evaluate the

involvement of CSF1R in the generation of Ly6C^{low} Treml4^{+} monocytes from Ly6C^{high} monocytes.

Figure 5: LysM-cre is often not very efficient in reducing gene expression in monocytes. What percentage of expression of GFPT1 was lost in classical, intermediate and nonclassical monocytes in these mice at baseline?

As described above, we cell-sorted splenic Ly6C^{high} Treml4^{-}, Ly6C^{+} Treml4^{+} and Ly6C^{low} Treml4^{+} monocytes and performed a PCR (**new Supplementary Figure 6D**). Our data revealed that Lyz2^{cre} efficiently ablated GFPT1 expression in all monocyte subsets (**new Supplementary Figure 6D**). The deletion efficiency varied from nearly 60% in Ly6C^{high} Treml4^{-} monocytes to 70% in Ly6C^{+} Treml4^{+} and Ly6C^{low} Treml4^{+} cells (**new Supplementary Figure 6D**).

Figure 5: The most novel aspect of this work is the metabolic work in Figure 5, although the loss of GFPT1 doesnt have a huge impact it seems. An analysis of Ly6C low and Ly6Chi monocyte metabolism, with and without the AFS98 Rx, using Seahorse, would be an important addition to this study.

We would like to thank the reviewer for this suggestion. As suggested by the reviewer we performed Seahorse analyses to better define the impact of GFPT1 deletion on intracellular metabolism. Since Seahorse requires many cells and we could only sort little quantities of monocytes (and the sorting procedure negatively affects their viability), we performed Seahorse of bone marrow-derived macrophages. These newly generated data were included in the revised version of the manuscript (**new Supplementary Figure 6I**). To explore monocyte metabolism in control and Lyz2$^{\Delta GFPT1}$ monocytes, we performed a glucose uptake assay and quantified their puromycin incorporation, reflecting ribosomal activity (**new Figures 6I-J**).

Overall: since CSFR1 inhibitory antibodies have been approved for use in humans, it is very important to measure the glucose metabolic pathways in human classical and nonclassical monocytes with CSFR1 inhibition. If not possible to obtain the antibody, then at least baseline glucose and mitochondrial measures should be shown.

We included newly generated data analyzing human monocyte metabolism from healthy donors at steady state and following overnight culture with receptor tyrosine kinase inhibitors (**new Figure 7**). First, we observed that, like in mice (**new Figures 6B and 6I**), glucose uptake was higher in human classical compared to non-classical monocytes (**new Figure 7A**). SCENITH analyses suggested that human classical monocytes are highly glycolytic while mitochondrial metabolism is also active in non-classical monocytes[9]. We previously reported a similar role of glucose intracellular metabolism on CSF1R expression in murine and human monocytes[8]. Together, these results suggest that our observations in mice could be translated to human monocytes. Our data indicated that blocking the activity of CSF1R and FLT3 reprograms classical monocyte metabolic configuration from glucose to mitochondrial dependence (**new Figure 7C-F**). Yet FLT3 involvement appears higher in human monocytes, further suggesting that despite many similarities, monocytes from different species rely to distinctive degree on RTKs for their differentiation and survival.

Bibliography

1 Thierry, G. R. *et al.* Non-classical monocytes scavenge the growth factor CSF1 from endothelial cells in the peripheral vascular tree to ensure survival and homeostasis. *Immunity* **57**, 2108–2121 e2106 (2024). https://doi.org/10.1016/j.immuni.2024.07.005

2 Ghigo, C. *et al.* Multicolor fate mapping of Langerhans cell homeostasis. *J Exp Med* **210**, 1657–1664 (2013). https://doi.org/10.1084/jem.20130403

3 Wolff, D. *et al.* Axatilimab in Recurrent or Refractory Chronic Graft-versus-Host Disease. *N Engl J Med* **391**, 1002–1014 (2024). https://doi.org/10.1056/NEJMoa2401537

4 Liu, Z. *et al.* Fate Mapping via Ms4a3-Expression History Traces Monocyte-Derived Cells. *Cell* **178**, 1509–1525 e1519 (2019). https://doi.org/10.1016/j.cell.2019.08.009

5 Trzebanski, S. *et al.* Classical monocyte ontogeny dictates their functions and fates as tissue macrophages. *Immunity* **57**, 1225–1242 e1226 (2024). https://doi.org/10.1016/j.immuni.2024.04.019

6 Jacquelin, S. *et al.* Homozygous kinase-dead Csf1r mutation in outbred mice reveals essential and redundant functions of tissue resident macrophages. *bioRxiv*, 2025.2007.2008.663807 (2025). https://doi.org/10.1101/2025.07.08.663807

7 Durai, V. *et al.* Altered compensatory cytokine signaling underlies the discrepancy between Flt3(-/-) and Flt3l(-/-) mice. *J Exp Med* **215**, 1417–1435 (2018). https://doi.org/10.1084/jem.20171784

8 Gallerand, A. *et al.* Glucose metabolism controls monocyte homeostasis and migration but has no impact on atherosclerosis development in mice. *Nat Commun* **15**, 9027 (2024). https://doi.org/10.1038/s41467-024-53267-5

9 Arguello, R. J. *et al.* SCENITH: A Flow Cytometry-Based Method to Functionally Profile Energy Metabolism with Single-Cell Resolution. *Cell Metab* **32**, 1063–1075 e1067 (2020). https://doi.org/10.1016/j.cmet.2020.11.007

REVIEWERS' COMMENTS

Reviewer #2 (Remarks to the Author):

The authors have included new data and revised the manuscript in accordance with our suggestions.
We thank the reviewer for the careful evaluation of our manuscript.

We still find it somewhat difficult to follow the central story and identify the take-home messages. Emphasizing these, at the end of the intro or start of the discussion could help.
We updated the introduction to clarify the take-home messages.

The authors also might the main text want to consider to move some of the more technical details to the supplement. In fact they could move the FIRE mice to there to streamline.
Although we understand the reviewer's point of view, we chose to keep the data on FIRE mice in the main figures, as the experiments address a discrepancy that is important to the field.

As previously noted, this manuscript reveals a number of interesting fact, although not all are entirely new. It has f.i. been reported that both GMP- and MDP-Mo give rise to NCM (see ref# 10); This should be acknowledged in line # 339.
We acknowledged that this has been reported before. However, this was done using surface markers or adoptive transfer experiments that do not allow to determine whether the ability of GMP- and MDP-derived cells to generate NCM is equivalent, or whether one lineage is favored in unmanipulated mice. Our data solve this question.

While I am not a metabolome expert, I felt this part somewhat underdeveloped, and the title is hence maybe not entirely appropriate.
Findings linked to metabolism are now better presented in the introduction.

As asked by the editor, I also went over the author's response to the critique of reviewer 1. To my mind, the authors also answered here well and sufficiently. Point 1 was a misunderstanding, and the other points are addressed by new data.
I think this study is ready to be published.
We thank the reviewer for going over our responses to reviewer 1's comments.

Reviewer #3 (Remarks to the Author):

The authors performed a significant number of innovative experiments to address my concerns. My questions and comments have been fully addressed.
We thank the reviewer for the careful evaluation of our manuscript.
